# Dryinones: Structure Elucidation of Red Colorants from Submerged Cultures of *Pleurotus dryinus*

**DOI:** 10.1021/acs.jnatprod.5c00926

**Published:** 2025-11-03

**Authors:** Niklas Broel, Johanna V. Stein, Franziska V. Wengner, Marvin H. J. Domanski, Tatyana Zhuk, Martin Gand

**Affiliations:** † Institute of Food Chemistry and Food Biotechnology, 9175 Justus Liebig University Giessen, Heinrich-Buff-Ring 17, 35392 Giessen, Germany; ‡ Institute of Organic Chemistry, Justus Liebig University Giessen, Heinrich-Buff-Ring 17, 35392 Giessen, Germany; § Faculty of Chemical Technology, Igor Sikorsky Kyiv Polytechnic Institute, Beresteiskyi Avenue 37, 03056 Kyiv, Ukraine

## Abstract

Culture supernatants of the veiled oyster mushroom *Pleurotus dryinus* from the phylum Basidiomycota develop a deep burgundy-red coloration when supplemented with phenylalanine. This marks the first time that color formation for *P. dryinus*, a fungus that is ordinarily colorless, has been reported. The two main coloring secondary metabolites, named dryinones, were isolated, and their structures were elucidated by HR-ESI­(+)–MS/MS, UV/vis, and multidimensional NMR spectroscopy. The colorants were structurally characterized as highly oxygenated meroterpenoids with an aminoquinone moiety as the main chromophore. The absolute configuration of the isolated dryinone A (**1**) was determined through NOESY correlations, ECD experiments, and DFT computations. These findings not only expand the chemical diversity of colorants within the phylum Basidiomycota but also demonstrate the biosynthetic versatility of *P. dryinus* under controlled culture conditions. The discovery of these aminoquinone-containing meroterpenoids provides new insights into fungal secondary metabolism and highlights the potential of edible mushrooms as underexplored sources of structurally unique natural products.

## Graphical abstract

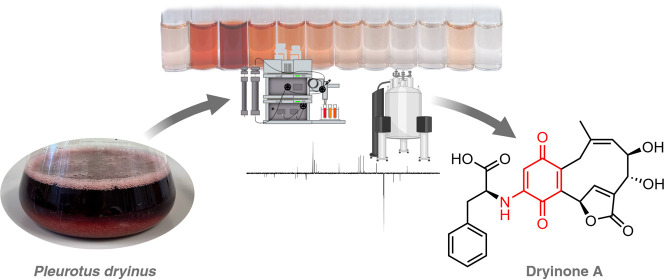

The Pleurotaceae family comprises a number of well-known and highly valued mushrooms, including the oyster mushroom (*Pleurotus ostreatus*), the king trumpet mushroom (*Pleurotus eryngii*), and the golden oyster mushroom (*Pleurotus citrinopileatus*).^1^ Another member of this family is the edible veiled oyster mushroom *Pleurotus dryinus*. Due to its relatively firm texture, *P. dryinus* is not as widespread as its well-known relatives, but the young fruit bodies in particular are suitable for consumption. While many members of this family exhibit no coloration of their fruiting bodies, there are species with distinct coloration, such as the aforementioned yellow *P. citrinopileatus* ^1^ or the pink oyster mushroom *Pleurotus salmoneostramineus*.^2,3^ These conspicuously colored varieties have already aroused interest in identifying the colorants responsible for the coloring of the fruiting bodies.

But, to the best of our knowledge, there is no literature available on colorant production by the veiled oyster mushroom *P. dryinus*. Fungi from the phylum Basidiomycota are known to produce various quinones as secondary metabolites, which may be used as pharmaceuticals or colorants, as they often exhibit useful bioactivity or visually appealing properties.^4^ The majority of them are 1,4-quinones, which are biosynthesized by polyketide synthases. Further interesting fungal colorants are aminoquinones due to their special UV/vis absorption. The red aminobenzoquinone lilacinone is formed by *Lactarius lilacinus*, a fungus of the phylum Basidiomycota, in which it is responsible for the lilac coloration of its fruiting body.^5^ Compared to unsubstituted benzoquinones, aminobenzoquinones generally show a bathochromic shift in their absorption spectrum due to the electron rich nitrogen substituent.^6^ This phenomenon is observed within the clavilactone class of compounds, where the addition of an amino group to the quinone core of the yellow clavilactone B gives the red clavilactone D.^7,8^ Clavilactones belong to meroterpenoids,^9^ a hybrid class, partially derived from terpenoid pathways,^10^ containing numerous compounds produced by animals,^11,12^ plants,^13^ bacteria^14^ and fungi.^10,15,16^ The class in general is known for a variety of significant biological activities, including antibacterial,^17^ antitumor^18–20^ and anti-inflammatory^21,22^ activities. Arnone et al. were the first to isolate the clavilactones A–C from the mycelium of *Clitocybe clavipes*,^7^ a fungus from the phylum Basidiomycota. The structures are characterized by a conformationally rigid ten-membered carbocycle with an annealed *R*,β-epoxy-γ-lactone connected to a hydroquinone or benzoquinone.^23^ In 2000, Merlini et al. isolated the clavilactones D and E from the same fungus.^18^

Several groups have made great efforts in semi and total synthesis of different clavilactones.^23–28^ Takao et al. revised the structure of clavilactone D in 2017 and confirmed that the correct structure has an amino group at C-3 instead of a hydroxy group at C-2 in the originally proposed structure.^8^ Clavilactone F was described by Sun et al. in 2019 as a constitutional isomer of clavilactone D with the amino group at C-2.^29^ Followed by clavilactones G–I, with clavilactone H being the first structure where the epoxy-function of clavilactones *R*,β-epoxy-γ-lactone moiety is substituted by a double bond.^30^ Clavilactones J and K were identified by Hou et al. in 2022, with the yellow clavilactone K depicting the first with an attached peptide bond.^31^ In 2023, Sun et al. claimed to first report on aminoglycoside meroterpenoids in nature, identifying clavilactones M–P.^9^ So far, various clavilactones have been identified, most from *C. clavipes*. Furthermore, Sun et al. identified two kinds of novel nitrogen-containing meroterpenoids, clavipyrrine A^32^ and the purple clavipines A–C^29^ from *C. clavipes*.

However, nothing is known about clavilactone-like structures from *Pleurotus* sp. This study reports the isolation and characterization of two novel, highly oxygenated, meroterpenoid colorants, dryinone A and dryinone B, from phenylalanine-induced cultures of *P. dryinus*. This finding not only expands our understanding of the diverse metabolic capabilities of edible mushrooms but also underscores their potential as a rich source of structurally unique natural products.

## Results and Discussion

### Cultivation and Isolation

The cultivation of *P. dryinus* in standard nutrient solution, supplemented with phenylalanine, leads to the formation of red colorants, resulting in a deep burgundy-red coloration of the culture supernatant. *P. dryinus* was identified via phylogenetic analysis, comparing the internal transcribed spacer (ITS) sequences of the fungus with different Pleurotaceae and related genera using similar references as Menolli et al.^33^ (Figure S1). The coloring compounds were extracted from freeze-dried culture supernatant, yielding a crude extract in which two major contributors to the overall color were identified via reversed-phase high-performance liquid chromatography with diode array detection (RP-HPLC–DAD) (Figure S2). The elution with increasing MeOH concentrations gave rise to eight fractions, of which fraction 6 (50% MeOH) exhibited the most intense red color. RP-HPLC–DAD analysis of these eluates revealed that fraction 6 contained two main compounds **1** and **2**. Both compounds showed similar absorption spectra with maxima at approximately 280 and 480 nm (Figure S3). In order to reach sufficient purity for NMR and HR-ESI­(+)–MS experiments, fraction 6 was further purified in a two-step preparative HPLC separation.

The first preparative separation of fraction 6 resulted not only in the isolation of compounds **1** and **2**, but also revealed that **2** exists in equilibrium with an yet unknown isomer, exhibiting identical UV/vis spectra (Figures S4 and S5). Analytical HPLC using a phenyl column achieved baseline separation of these isomers (*t*_R_ = 11.51 and 11.87 min; Figure S5). Therefore, and for further purification of the colorants, a phenyl-hexyl column was used for a second step of preparative purification. Subsequent investigations revealed a time-dependent conversion of the two isomers of **2** into each other, resulting in the establishment of an equilibrium favoring the compound at a *t*_R_ of 11.51 min with a ratio (*A*_11.51 min_/*A*_11.87 min_) of 1.6 (Figures S5 and S6). In further structure elucidation experiments for **2**, the dominant peak at a *t*_R_ of 11.51 min was investigated. The purity of **1** was likewise improved via phenyl-hexyl preparative HPLC while confirming the isolation of only one isomer (Figure S7). Finally, the isolation process yielded 7.7 mg of **1** and 12.5 mg of **2**, with a purity greater than 95% based on RP-HPLC–DAD analysis (Figure 1).

**Figure 1:**
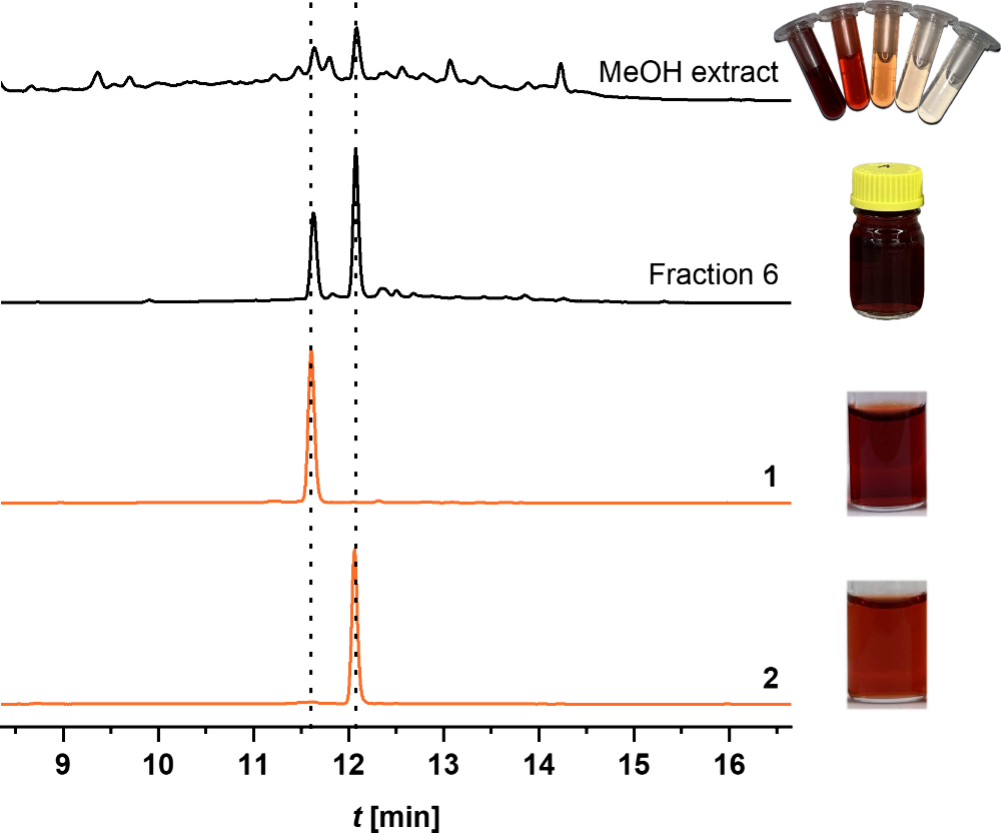
RP-HPLC–DAD chromatograms at 254 nm of the methanolic crude extract, the fraction 6 of the column chromatography as well as **1** and **2** after the two-step preparative HPLC separation. Analysis was performed on a C-18 column and the dashed lines mark the *t*_R_ of the red colorants.

### Structure Elucidation

The HR-ESI­(+)–MS spectrum of **1** showed a base peak at *m*/*z* 488.1309, which was identified as [M + Na]^+^, yielding a monoisotopic mass (*M*_monoisotopic_) for **1** of 465.1417 Da (Figure S8). From this, the molecular formula C_25_H_23_NO_8_ (calc. *M*_monoisotopic_ = 465.1424 Da) was calculated with a deviation of 1.43 ppm, resulting in 15 degrees of unsaturation. HR-ESI­(+)–MS/MS experiments with **1** demonstrated neutral losses of CO_2_ and H_2_O for the fragmentation of the [M + Na]^+^ signal (Figure S9). This finding could indicate the presence of a carboxyl and a hydroxy group in the target molecule. Furthermore, the presence of a tropylium cation [C_7_H_7_]^+^ was detected at *m*/*z* 91.0540, which could originate from a benzyl moiety in **1**. The HR-ESI­(+)–MS experiments for **2** resulted in an almost identical spectrum with an [M + Na]^+^ signal at 488.1306 Da, which, together with the identical UV/vis spectra, argues in favor of **2** being an isomer of compound **1** (Figure S10). Notably, the mass spectra of the two compounds observed during the purification of **2** are identical.

The 1D and 2D NMR experiments carried out on **1** and **2** showed a very high degree of similarity (Table 1; **1**, Figures S11–S20; **2**, Figures S21–S27). First, the structure of **1** was elucidated based on the obtained data. Combination of ^1^H, ^13^C, *J*-modulated ^13^C and HSQC NMR experiments led to the identification of two carbonyls C-1 and C-4 (δ_C_: 185.3 and 183.0), which most likely belong to a 1,4-quinone,^34^ one carboxylic carbon C-17 (δ_C_: 173.4), one ester carbonyl C-15 (δ_C_: 174.0), three oxymethines C-6, C-9, and C-10 (δ_C_: 77.3, 71.8, and 75.9), seven sp^2^-hybridized carbons C-3, C-5, C-7, C-8, C-11, C-12, and C-14 (δ_C_: 147.1, 149.8, 147.0, 137.5, 125.9, 138.5, and 137.4), besides a monosubstituted benzene C-20–C-25 (δ_C_: 137.6, 130.3, 129.6, 128.1, 129.6, and 130.3) as well as two methylenes C-13 and C-19 (δ_C_: 30.4 and 37.8), one methine C-18 (δ_C_: 57.49), and one methyl carbon C-16 (δ_C_: 22.2).

**Table 1:** ^1^H NMR (CD_3_OD, 700 MHz) and ^13^C NMR (CD_3_OD, 176 MHz) Data and ^1^H–^13^C and ^1^H–^15^N HMBC Correlations Observed for Compounds **1** and **2**

	**1**					**2**				
				HMBC^a^					HMBC^a^	
number	δ_C_	C type	δ_H_ (*J* in Hz)	^1^H–^13^C	^1^H–^15^N	δ_C_	C type	δ_H_ (*J* in Hz)	^1^H–^13^C	^1^H–^15^N
1	185.3	C				184.9	C			
2	100.0	CH	5.59, s	1, 3, 4, 5, 13, 14	N(1)	147.2	C			
3	147.1	C				99.5	CH	5.57, s	1, 2, 5, 6	N(1)
4	183.0	C				184.2	C			
5	149.8	C				141.7	C			
6	77.3	CH	6.39, s	4, 5, 7, 10, 14, 15		77.2	CH	6.52, s	4, 5, 7, 10, 14, 15	
7	147.0	CH	7.79, s	6, 8, 9, 10, 13, 15		147.6	CH	7.82, s	6, 8, 9, 10, 13, 15	
8	137.5	C				137.3	C			
9	71.8	CH	4.33, d (8.1)	7, 8, 10, 15		71.8	CH	4.33, d (8.1)	7, 8, 10, 15	
10	75.9	CH	4.40, t (8.3)	9, 11, 12		75.8	CH	4.41, t (8.4)	9, 11, 12	
11	125.9	CH	5.02, d (8.6)	13, 16		126.2	CH	5.05, d (8.6)	13, 16	
12	138.5	C				138.0	C			
13	30.4	CH_2_	3.76, d (14.4), a	1, 5, 11, 12, 14		30.2	CH_2_	3.57, d (14.8), a	1, 5, 11, 12, 14	
			3.17, d (13.9), b^b^	1, 5, 11, 12, 14, 16				3.23, d (14.9), b	1, 5, 11, 12, 14, 16	
14	137.4	C				137.4	C			
15	174.0	C				174.0	C			
16	22.2	CH_3_	1.35, s	9, 11, 12, 13		22.1	CH_3_	1.35, s	9, 11, 12, 13	
17	173.4	C				173.4	C			
18	57.4	CH	4.37, dd (7.7, 5.0)^c^	3, 17, 19, 20	N(1)	57.1	CH	4.38, t (6.2)^c^	2, 17, 19, 20	N(1)
19	37.8	CH_2_	3.33, d (5.1), a^d^	17, 18, 20, 21, 25	N(1)	37.7	CH_2_	3.33, d (5.2), a^d^	17, 18, 20, 21, 25	N(1)
			3.16, dd (14.2, 4.5), b^b^	17, 18, 20, 21, 25	N(1)			3.14, dd (14.0, 7.2), b	17, 18, 20, 21, 25	N(1)
20	137.6	C				137.5	C			
21	130.3	CH_(Ar)_	7.20–7.26, m	19, 20		130.4	CH_(Ar)_	7.19–7.28, m	19, 20	
22	129.6	CH_(Ar)_	7.20–7.26, m	19, 20		129.5	CH_(Ar)_	7.19–7.28, m	19, 20	
23	128.1	CH_(Ar)_	7.20–7.26, m	19, 20		128.2	CH_(Ar)_	7.19–7.28, m	19, 20	
24	129.6	CH_(Ar)_	7.20–7.26, m	19, 20		129.5	CH_(Ar)_	7.19–7.28, m	19, 20	
25	130.3	CH_(Ar)_	7.20–7.26, m	19, 20		130.4	CH_(Ar)_	7.19–7.28, m	19, 20	

a HMBC correlations.

b Overlapping.

c Not fully resolved.

d Obscured by solvent signal (CD_3_OD).

Furthermore, noteworthy are the diastereotopic proton pairs at C-19 (δ_H_: 3.33 and 3.16) and C-13 (δ_H_: 3.76 and 3.17), identified via HSQC and large coupling constants in proton NMR (e.g., H-13a *J* = 14.4 Hz; H-13b *J* = 13.9 Hz) representing geminal couplings. The identification of an ABX system comprising H-18 and the diastereotopic protons H-19a and H-19b, in conjunction with the HMBC and ^1^H–^1^H COSY data, resulted in the identification of a phenylalanine moiety as the first building block of **1** (Figure 2). For C-2 with a carbon shift of 100.0 ppm and a corresponding proton shift of H-2 of 5.59 ppm, a direct assignment of this carbon was not possible since its signal appears at a higher field than usual sp^2^-hybridized methine groups.^34^ Considering the calculated molecular formula as well as the ^1^H–^15^N HMBC data, the shift of C-2 could be explained by the presence of an aminoquinone system, in which a sp^2^-hybridized carbon (C-2; δ_C_: 100.0) next to the nitrogen-bound carbon (C-3; δ_C_: 147.1) tends to show a significantly upfield-shifted signal as observed for C-2.^8,28,29^ The combination of these data with the HMBC correlations led to the assignment of a phenylalanine moiety bound to an aminoquinone core, additionally comprising C-1 to C-5 and C-14 (δ_C_: 185.3, 100.0, 147.1, 183.0, 149.8, and 137.4). Starting from the quaternary carbons C-5 and C-14, the analysis of 2D NMR experiments led to the assembly of a ten-membered carbocycle. In this structure, two vinylic carbons (C-11 and C-12; δ_C_: 125.9 and 138.5) along with the methyl carbon C-16 (δ_C_: 22.2) and the methylene carbon C-13 (δ_C_: 30.4) form a strong spin system clearly observed in the ^1^H–^1^H COSY spectrum. Moreover, the ten-membered carbocycle provides an explanation for the diastereotopic protons H-13a and H-13b (δ_H_: 3.76 and 3.17) as it restricts the free rotation of the bonds at C-13. Besides the incorporation of the two oxymethines C-10 and C-9 (δ_C_: 75.9 and 71.8), the ring is closed back to C-5 of the aminoquinone core via C-6, C-7 and C-8 (δ_C_: 77.3, 147.0, and 137.5). Notably, H-6 and H-7 exhibit unusual downfield shifts at 6.39 and 7.79 ppm, respectively.

**Figure 2:**
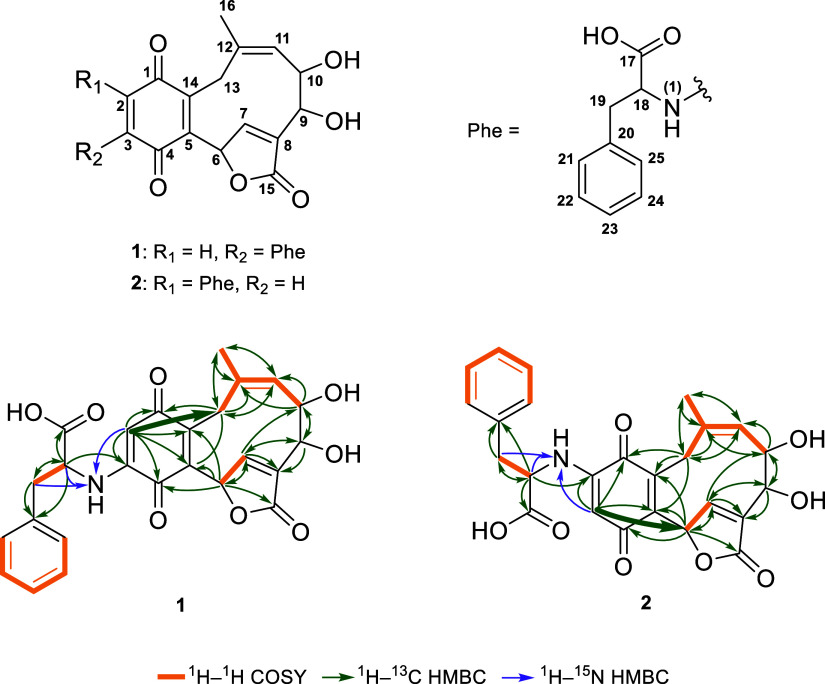
Structures of the compounds **1** and **2** as well as key ^1^H–^1^H COSY, ^1^H–^13^C HMBC and ^1^H–^15^N HMBC correlations. Bold arrows mark the key ^1^H–^13^C HMBC correlation for differentiation between **1** and **2**.

To verify the position of the C–N bond linking the quinone core and the phenylalanine fragment, the 1D and HMBC spectra were further utilized. The investigation of the long-range couplings of the quinone proton revealed significant differences between the spectral sets of **1** and **2**. Regarding **1**, the quinone proton H-2 exhibited a cross peak with C-13 (^4^ *J*_CH_), which clearly indicates the position of the quinone proton at C-2, implying that the position of the C–N bond must be at C-3. In contrast, the quinone proton of **2** exhibited such a long-range coupling with C-6 (^4^ *J*_CH_), indicating that the C–N bond must be at C-2 in this instance. These observations lead to the identification of **1** and **2** as regioisomers differing in the linkage between the meroterpenoid core and the bound amino acid. Furthermore, in both **1** and **2**, the presence of C-8, classified as a quaternary vinylic carbon, along with C-6, identified as an oxymethine, and the observed ^1^H–^13^C HMBC correlations between H-6 and C-15, collectively contribute to the identification of an annealed α,β-unsaturated-γ-lactone ring. The structures identified in this study, named dryinones, are previously undescribed meroterpenoid aminoquinone colorants, which exhibit structural similarity to the clavilactones first isolated from *C. clavipes*. Identified by Sun et al. in 2019,^29^ the clavipines, clavilactone derivatives containing an aminoquinone moiety, play a special role in this context, as their structures are related to the dryinones and their spectroscopic data correspond well with the data reported in this work. Sun et al. placed a particular focus on the identification of the nitrogen position within the aminoquinone, as it was previously done by Lv et al.,^28^ Takao et al.^8^ and others for different clavilactone derivatives. Sun et al.^29^ concluded that when the C–N bond is located at C-3, C-5 of the aminoquinone core exhibited a considerably downfield-shifted signal at around 150 ppm, while the C-2 regioisomer exhibited a highfield-shifted signal for C-5 at around 145 ppm. The chemical shifts observed for C-5 (149.8 ppm for **1** and 141.7 ppm for **2**) in the ^13^C NMR spectrum, along with consistent trends in the proton NMR (of H-6, H-13a, and H-13b) further support the proposed structures of **1** and **2**. It is imperative to note that three structural features of the dryinones described herein are of particular importance. First, the structures characterized in this study are clavilactone derivatives with an α,β-unsaturated-γ-lactone ring instead of an α,β-epoxy-γ-lactone, which is typical for numerous clavilactones. It needs to be mentioned that these α,β-unsaturated-γ-lactone derivatives have been previously described in the form of clavilactone H^30^ and clavipine C^29^ isolated from *C. clavipes* and arnebiol A^35^ isolated from the plant *Arnebia euchroma*. A secondary distinctive feature of the structure outlined is the diol structure at positions C-9 and C-10. To date, only clavilactone G has been described as a derivative bearing a hydroxy group at C-9.^30^ The key structural feature responsible for the red color of the dryinones is the phenylalanine attached to the quinone through its amino group. This attachment forms an aminoquinone chromophore system, causing a bathochromic shift compared to the absorption spectra of a typical 1,4-benzoquinone.

### Absolute Configuration

Subsequently, the absolute configuration of the isolated compounds **1** and **2** was analyzed using NOESY and ECD experiments. While reliable spectra could not be recorded for **2,** since this compound is subject to constant isomerization, only the absolute configuration of **1** was assessed. Initially, a series of theoretical molecules was determined using density functional theory (DFT) computations, assuming an 18*S* configuration due to the addition of l-phenylalanine. For these theoretical isomers of **1**, both UV/vis and ECD spectra were calculated (Figures S28–S36) and compared with the spectra determined experimentally. Furthermore, one exemplary isomer of **2** was additionally calculated (Figure S37) to prove that no significant effects on the UV/vis spectra are present and the experimental UV/vis spectrum is in line with the structure of **2**. The calculated UV/vis spectra show no significant differences between the different isomers or between the structures of **1** and **2**. Moreover, these theoretical spectra fit well with the experimental spectra for **1**, exhibiting three distinct maxima at 216.3 nm (**1** exp.: 207.3 nm), 272.8 nm (**1** exp.: 279.9 nm) and 484.4 nm (**1** exp.: 475.7 nm). The calculated ECD spectra provided a decisive indication of the absolute configuration of the C-6 carbon. All calculated spectra for (6*S*) isomers showed a negative band at a wavelength of approximately 220 nm, while all (6*R*) isomers showed a positive band at this wavelength. Considering the theoretical ECD spectra of all eight possible isomers of **1**, only the theoretical spectrum for the (6*R*,9*R*,10*R*,18*S*) isomer matched the experimentally determined ECD spectrum for **1,** especially considering the positive Cotton effect with a crossing point at approximately 210 nm (Figure 3).

**Figure 3:**
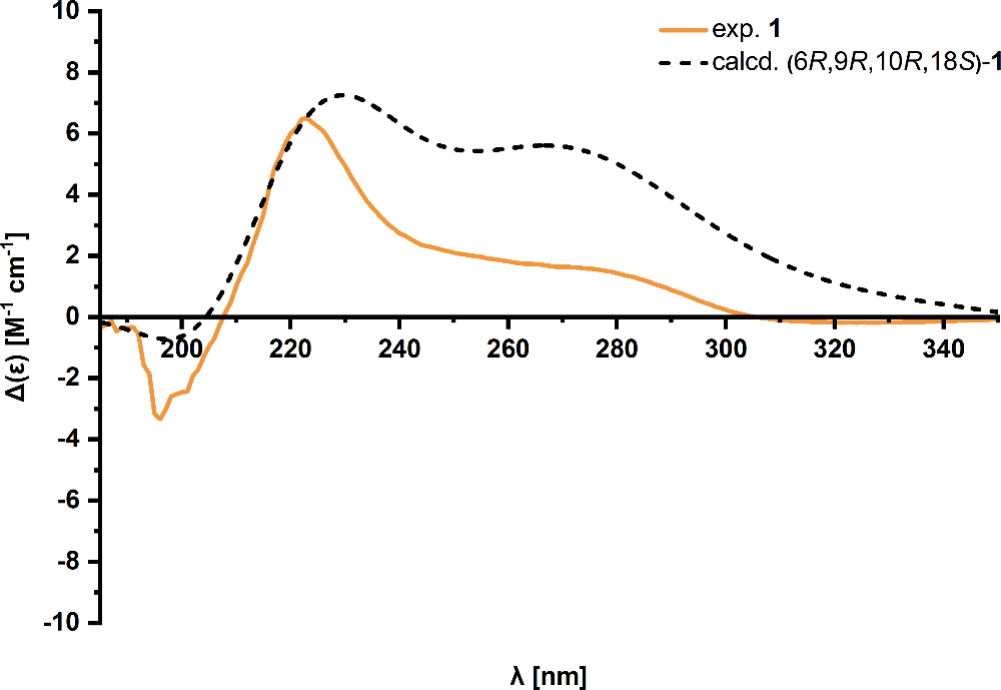
Experimental ECD spectrum of **1** and the time-dependent DFT spectrum computed at the CAM-B3LYP/def2-TZVP/CPCM­(MeOH) level of theory using *n*_roots_ = 20 for the (6*R*,9*R*,10*R*,18*S*) isomer of **1**.

The assigned configurations can be confirmed through analysis of the NOESY spectrum of **1**, giving information about the relative conformations inside the carbocycle (Figure 4). The observed correlations between H-7 (7.79 ppm) and H-6 (6.39 ppm), H-13b (3.17 ppm), and H-10 (4.40 ppm) and between H-13b and H-10, were found to be of significance. This is due to the fact that these correlations prove the transannular interactions of the involved protons, which are only possible if these are oriented toward the same side of the ten-membered carbocycle, which further strengthens the assignment as 6*R*,9*R*,10*R*. Besides that, the (*Z*) orientation of the substituents at double bond Δ^11^ could be verified via the correlation between H_3_-16 (1.35 ppm) and H-11 (5.02 ppm), proving that they face in the same direction, which is visible in the NOESY spectrum. The combination of these data resulted in the elucidation of the absolute configuration of the red colorant dryinone A (**1**).

**Figure 4:**
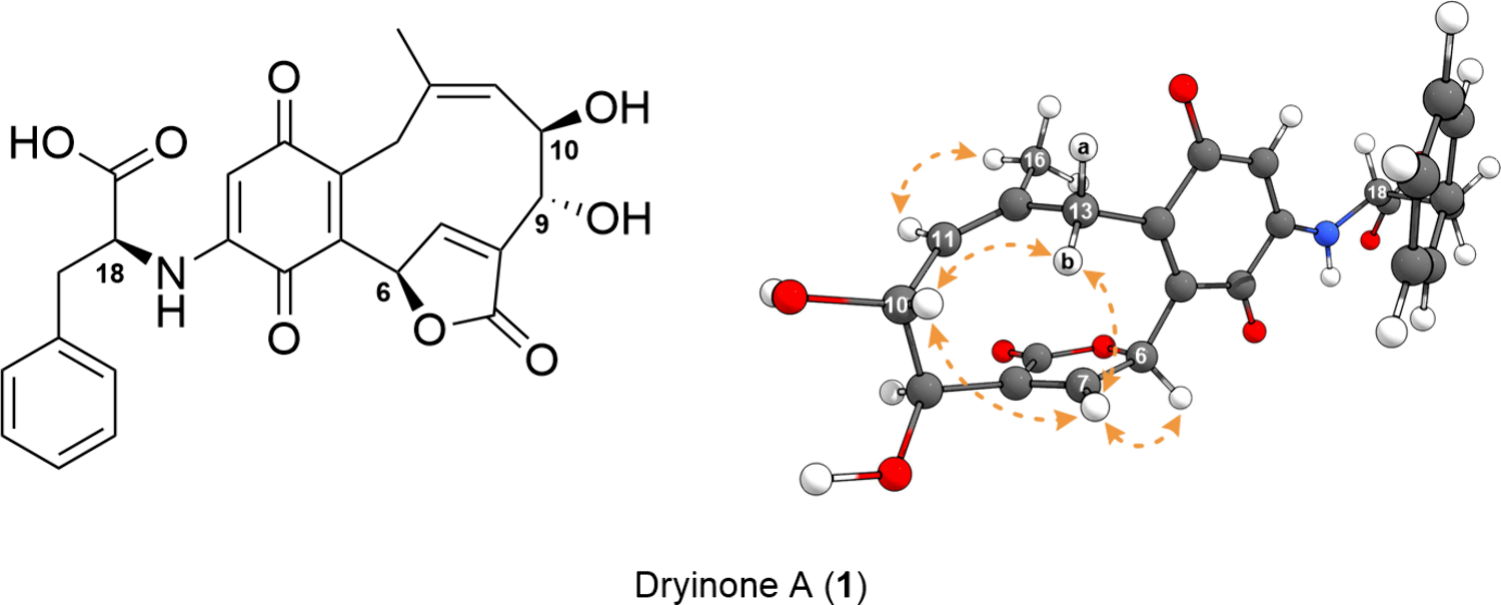
Absolute 6*R*,9*R*,10*R*,18*S* configuration of **1** and key NOESY correlations (orange arrows), which are visualized in the corresponding computationally generated isomer.

Herein, colorant formation by the veiled oyster mushroom could be described when it is cultivated in phenylalanine supplemented medium. The key coloring compounds were isolated from the culture supernatant and the structures of two novel red meroterpenoid colorants, dryinone A (**1**) and dryinone B (**2**), comprising a rare aminoquinone chromophore, were elucidated by NMR and MS experiments. For dryinone A, the absolute configuration was established by ECD experiments as well as by NOESY NMR. Besides the identification of these novel aminoquinone colorants, this study presents the first evidence for the formation of colorants for *P. dryinus* and the first identification of clavilactone derivatives produced by a fungus belonging to the Pleurotaceae family. Moreover, a deeper investigation of the biosynthesis of these highly complex and unusual colorants could yield some interesting enzymes and gain crucial knowledge on the biosynthetic capabilities of *P. dryinus* and fungi in general.

## Experimental Section

### General Experimental Procedures

ECD spectra (185 – 350 nm) were recorded in MeOH using a Jasco J-715 spectropolarimeter (JASCO, Tokyo, Japan). UV/vis spectra (190 – 800 nm) were recorded in MeOH utilizing a SI Analytics UviLine SI 7000 photometer (SI Analytics, Mainz, Germany). FTIR spectra were obtained using a Bruker ALPHA FTIR spectrometer (Bruker, Billerica, U.S.A.). ^1^H, ^13^C, *J*-modulated ^13^C, ^1^H–^13^C HSQC, ^1^H–^13^C HMBC, ^1^H–^15^N HMBC, ^1^H–^1^H COSY, and ^1^H–^1^H NOESY NMR spectra were recorded on a Bruker Avance NEO 700 MHz spectrometer (Bruker) equipped with a 5 mm TCI H&F-C/N-D Prodigy CryoProbe. 1D spectra were referenced to the residual solvent signals of CD_3_OD (δ_H_ 3.31 ppm, δ_C_ 49.0 ppm). HR-ESI­(+)–MS and MS/MS experiments were performed with a Bruker Impact II quadrupole-time-of-flight instrument. Purity of the isolates was continuously evaluated by means of analytical RP-HPLC–DAD (Shimadzu Prominence LC-20AD equipped with a SPD M20DAD; Shimadzu, Kyoto, Japan). If not mentioned otherwise, the analysis was conducted utilizing a Hypersil Gold C18 column (150 × 4.6 mm, 5 μm, Thermo Fisher, Waltham, U.S.A.) at 25 °C, using gradient elution. The mobile phase consisted of (A) 0.1% aqueous trifluoroacetic acid (TFA) and (B) MeOH. The following gradient program with a flow rate of 1 mL min^–1^ was used: 5% for 2 min, increasing the concentration of B to 90% in 10 min, 90% B for 2 min followed by a backflush to 5% B in 2 min and a final 3 min at 5% B. The DAD was configured to operate within a recording range of 230 to 800 nm. A further analytical HPLC method was developed using a phenyl phase in order to separate the isomers of **2**. A Zorbax Eclipse XDB-Phenyl column (150 × 4.6 mm, 3.5 μm, Agilent, Santa Clara, USA) was used in a gradient elution with (A) 0.1% TFA and (B) acetonitrile (ACN) with the following program: 5% B for 2 min, increasing the concentration of B to 100% in 18 min, 100% B for 3 min followed by a backflush to 5% B in 2 min and a final 2 min at 5% B. The flow rate was set to 1 mL min^–1^ and the column temperature to 25 °C.

### Fungal Material and Identification

The fungus *P. dryinus* was obtained from the German Collection of Microorganisms and Cell Cultures (DSMZ, Braunschweig, Germany; strain number DSM5178). The identity of the organism was confirmed by alignment of the internal transcribed spacer (ITS) sequences of 31 references sequences of related species obtained from GenBank (https://www.ncbi.nlm.nih.gov/genbank/). For the alignments, Geneious v.9.1 (Biomatters, Auckland, New Zealand) was used with the alignment type set as global with gaps open penalty of 13 and gap extension penalty of 3 using the cost matrix 65%. The phylogenetic tree was inferring the maximum likelihood^36^ using the PhyML plugin and the JC69 substitution model^37^ with 1000 bootstraps, all remaining parameters were set as default. Therefore, the DNA from *P. dryinus* cultures was isolated with the CTAB method as described previously.^38^ The internal transcribed spacer region was amplified and sequenced with primers ITS1_fw: TCCGT­AGGTG­AACCT­GCGG and ITS4_rv: TCCTC­CGCTT­ATTGA­TATGC according to White et al.^39^

### Fermentation and Extraction

The *P. dryinus* strain was kept on malt extract peptone agar (MEPA; 30 g L^–1^ malt extract, 3 g L^–1^ soy peptone, 15 g L^–1^ agar). The fungus was cultivated in a pre- and main culture cycle for colorant production. Initially, a 0.7 × 0.7 cm piece of overgrown agar was transferred to Erlenmeyer flasks with 40% filling volume of malt extract medium (ME; 20 g L^–1^ malt extract) to inoculate the preculture. The preculture was then subjected to homogenization for a period of 30 s with the use of an Ultra-Turrax (Ika, Staufen, Germany). Subsequently, the precultures were cultivated for 7 days on an orbital shaker at 150 rpm, 24 °C and in darkness. Color production was carried out in the main culture using standard nutrient solution according to Fraatz et al.^40^ containing 30 g L^–1^ d-glucose monohydrate (autoclaved separately), 4.5 g L^–1^ l-asparagine monohydrate, 1.5 g L^–1^ potassium dihydrogen phosphate, 0.5 g L^–1^ magnesium sulfate monohydrate, 3 g L^–1^ yeast extract and 1 mL L^–1^ trace element solution (EDTA 400 mg L^–1^, 90 mg L^–1^ ZnSO_4_·5H_2_O, 80 mg L^–1^ FeCl_3_·6H_2_O, 30 mg L^–1^ MnSO_4_·H_2_O, 5 mg L^–1^ CuSO_4_·5H_2_O) the pH was adjusted to 6.0 and the medium was further supplemented with 0.03 M l-phenylalanine (4.96 g L^–1^ l-phenylalanine). Therefore, after 7 days, the precultures were homogenized again for 30 s using an Ultra-Turrax. 10% of the homogenized preculture was then used to inoculate the main cultures, which were kept under the same conditions as the precultures. After 14 days, the main cultures were harvested by separation of mycelia from the colored culture supernatant via vacuum filtration. Thereafter, approximately 10 L of culture medium was subjected to freeze-drying (Christ Alpha 1-4 LSCplus, Martin Christ Gefriertrocknungsanlagen, Osterode am Harz, Germany) and exhaustive extraction with MeOH was carried out, yielding the crude colorant extract.

### Isolation and Structure Elucidation

The purity of the isolates was assessed via RP-HPLC–DAD analysis. As a first purification step, the crude extract was subjected to column chromatography using reversed-phase material (phenyl-modified silica, Macherey–Nagel, Düren, Germany). Elution was performed by a stepwise increasing MeOH content in a mixture with aqueous 0.1% TFA, starting from 5% MeOH to 100% MeOH. The fractions were afterward used for analytical HPLC. Fraction 6 was further subjected to preparative HPLC purification using a Knauer Azura Lab Prep LC50 system equipped with a single wavelength detector UVD 2.1S. For the first HPLC purification, a VP Nucleodur C18 ec column (250 × 16 mm, 5 μm, Macherey–Nagel) was used. The gradient program, consisting of (A) 0.1% TFA and (B) MeOH with a flow rate of 10 mL min^–1^ was as follows: 40% B for 2 min, increasing the concentration of B to 100% in 38 min and a final 10 min at 100% B. The wavelength for the detection was set at 254 nm. Fractions containing **1** were collected between 17 and 18 min and fractions containing **2** between 19 and 20.5 min. Fractions containing **1** and fractions containing **2** were separately subjected to a second preparative HPLC separation step using a Nucleodur Phenyl-Hexyl column (250 × 21 mm, 5 μm, Macherey–Nagel). The eluents were set as (A) 0.1% TFA and (B) ACN utilizing the following gradient program at a flow rate of 10 mL min^–1^: 2 min at 20% B, B increasing to 70% in 36 min, followed by a second steeper increase of B to 100% in 2 min and a final 10 min at 100% B. All fractions containing **1** or **2** were pooled and solvents removed under N_2_ flow. Dried isolates were subjected to HR-ESI­(+)–MS, UV/vis, ECD, and NMR experiments.

### Computational Methods

CREST^41,42^ version 3.0.1 was used to identify the most stable conformer for each dryinone A (**1**) isomer. The most stable molecular geometry of each isomer was reoptimized at the ωB97X^43^-D4 ^44^/def2-TZVPP^45^/SMD^46^­(MeOH)//r^2^SCAN-3c^47^/SMD­(MeOH) level of theory. All structures were characterized with analytical Hessian calculations to ensure real minima (*N*_imag_ = 0) using the ORCA 6.0.1 software package.^48,49^ To match the experimental conditions, the ECD and UV/vis spectra were computed with MeOH as the implicit solvent with the Conductor-like Polarizable Continuum Model^50^ (CPCM) at the CAM-B3LYP^51^/def2-TZVP/CPCM­(MeOH) level of theory using *n*_roots_ = 20. The resulting spectrum for each isomer was generated with Multiwfn.^52^

#### Dryinone A (**1**):

Red amorphous solid; UV/vis (MeOH) λ_max_ (log ε) 207.3 (4.55), 279.9 (3.83), 475.7 (3.30) nm; ECD (*c* = 5 mM, MeOH) λ_max_ (Δε) 196 (−3.3), 223 (+6.5) nm; FTIR (ATR, neat) ν_max_ 3351, 2919, 1737, 1668, 1630, 1588, 1510, 1439, 1295, 1198, 1165, 1097, 1033 cm^–1^; ^1^H NMR (CD_3_OD, 700 MHz) and ^13^C NMR (CD_3_OD, 176 MHz) (see Table 1); HR-ESI­(+)–MS *m*/*z* 466.1490 [M + H]^+^ (calcd. for C_25_H_23_NO_8_, 466.1497) and *m*/*z* 488.1309 [M + Na]^+^ (calcd. for C_25_H_23_NO_8_Na, 488.1316).

#### Dryinone B (**2**):

Red amorphous solid; UV/vis (MeOH) λ_max_ (log ε) 206.6 (4.52), 280.0 (3.78), 475.9 (3.29) nm; ECD (*c* = 6 mM, MeOH) λ_max_ (Δε) 202 (+2.8), 224 (+5.9) nm; FTIR (ATR, neat) ν_max_ 3359, 2937, 1732, 1668, 1634, 1590, 1509, 1441, 1294, 1194, 1167, 1097, 1044 cm^–1^; ^1^H NMR (CD_3_OD, 700 MHz) and ^13^C NMR (CD_3_OD, 176 MHz) (see Table 1); HR-ESI­(+)–MS *m*/*z* 466.1488 [M + H]^+^ (calcd. for C_25_H_23_NO_8_, 466.1497) and *m*/*z* 488.1306 [M + Na]^+^ (calcd. for C_25_H_23_NO_8_Na, 488.1316).

## Supplementary Material







## Data Availability

The ITS sequence of *P. dryinus* has been uploaded to the GenBank under the identifier PV895100. NMR data for dryinones A and B (**1** and **2**) have been deposited at the Natural Products Magnetic Resonance Database (NP-MRD; https://np-mrd.org/), NP-MRD ID NP0351208 for dryinone A (**1**) and NP0351194 for dryinone B (**2**).

## Notes

Raw NMR data for **1** (ZIP)
Raw NMR data for **2** (ZIP)
Phylogenetic tree, HPLC chromatograms of the crude colorant extract from standard nutrient solution supplemented with l-phenylalanine, UV/vis, IR, and HR-ESI­(+)–MS­(/MS) spectra of compounds **1** and **2** as well as the ECD spectrum of **2**, preparative HPLC chromatograms, analytical HPLC chromatogram of isomers of **2**, 1D and 2D NMR spectra of **1** and **2**, and details of the DFT computations and calcd. UV/vis and ECD spectra (PDF)
