# Supplementary material for: Dryinones: Structure Elucidation of Red Colorants from Submerged Cultures of Pleurotus dryinus
Source: J Nat Prod. 2025 Nov 3;88(11):2602–9. doi: 10.1021/acs.jnatprod.5c00926 (PMC12670701; doi:10.1021/acs.jnatprod.5c00926)
Supplement: Supplementary file 1 [file np5c00926_si_001.zip › NMR Data Dryinone A (1)/13C/pdata/1/email_Sep24-2024_700_NB_86_1.pdf]

Broel PDR2 6a

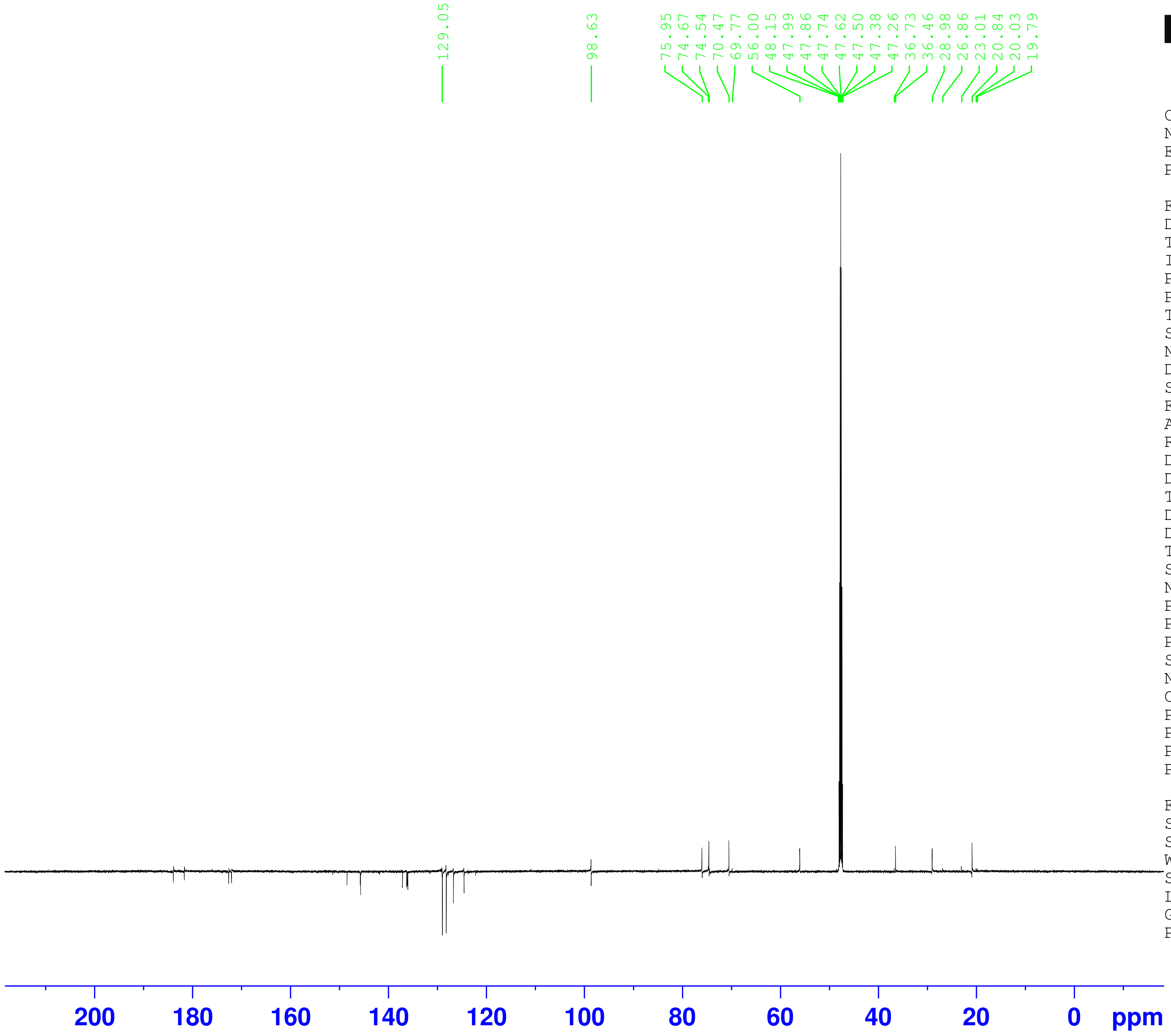

Current Data Parameters

NAME	Sep24-2024_700_NB
EXPNO	86
PROCNO	1

F2 - Acquisition Parameters

Date_	20240924
Time	17.23 h
INSTRUM	Avance Neo
PROBHD	Z168794_0004 (
PULPROG	zgpg30
TD	65536
SOLVENT	MeOD
NS	1024
DS	4
SWH	41666.668 Hz
FIDRES	1.271566 Hz
AQ	0.7864320 sec
RG	101
DW	12.000 usec
DE	18.00 usec
TE	303.0 K
D1	2.00000000 sec
D11	0.03000000 sec
TD0	1
SFO1	176.1031546 MHz
NUC1	13C
P0	4.00 usec
P1	12.00 usec
PLW1	110.76999664 W
SFO2	700.2828011 MHz
NUC2	1H
CPDPRG[2	waltz65
PCPD2	80.00 usec
PLW2	13.90999985 W
PLW12	0.13841000 W
PLW13	0.06950700 W

F2 - Processing parameters

SI	32768
SF	176.0855461 MHz
WDW	EM
SSB	0
LB	1.00 Hz
GB	0
PC	1.40
