# Supplementary material for: Dryinones: Structure Elucidation of Red Colorants from Submerged Cultures of Pleurotus dryinus
Source: J Nat Prod. 2025 Nov 3;88(11):2602–9. doi: 10.1021/acs.jnatprod.5c00926 (PMC12670701; doi:10.1021/acs.jnatprod.5c00926)
Supplement: Supplementary file 1 [file np5c00926_si_001.zip › NMR Data Dryinone A (1)/1H/pdata/1/email_Sep24-2024_700_NB_80_1.pdf]

Broel PDR2 6a

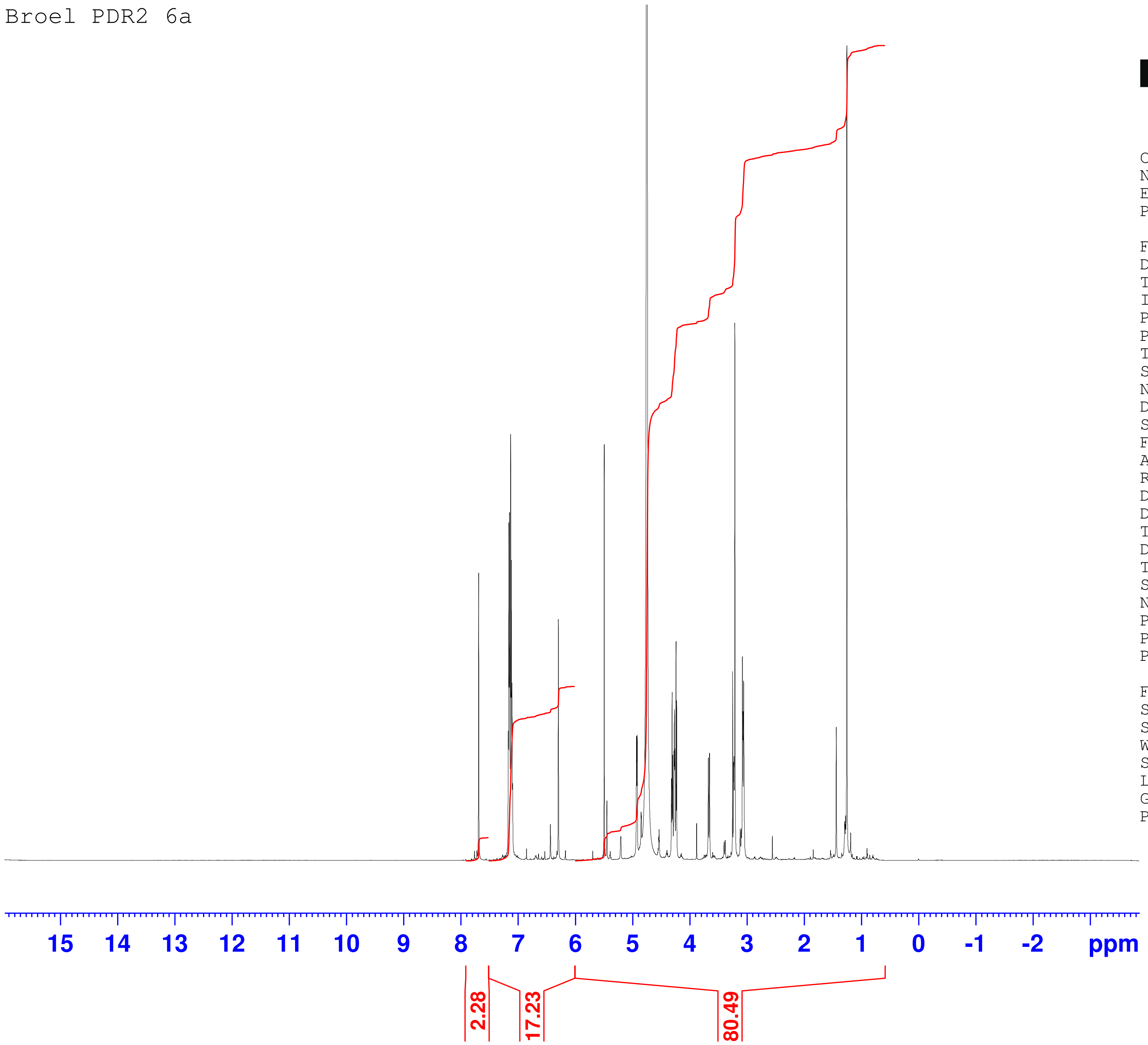

Current Data Parameters

NAME	Sep24-2024_700_NB
EXPNO	80
PROCNO	1

F2 - Acquisition Parameters

Date_	20240924
Time	12.49 h
INSTRUM	Avance Neo
PROBHD	Z168794_0004 (
PULPROG	zg30
TD	65536
SOLVENT	MeOD
NS	128
DS	2
SWH	13888.889 Hz
FIDRES	0.423855 Hz
AQ	2.3592961 sec
RG	74.5725
DW	36.000 usec
DE	12.14 usec
TE	303.0 K
D1	1.00000000 sec
TD0	1
SFO1	700.2843242 MHz
NUC1	1H
P0	2.66 usec
P1	7.98 usec
PLW1	13.90999985 W

F2 - Processing parameters

SI	65536
SF	700.2800837 MHz
WDW	EM
SSB	0
LB	0.30 Hz
GB	0
PC	1.00
