# Supplementary material for: Dryinones: Structure Elucidation of Red Colorants from Submerged Cultures of Pleurotus dryinus
Source: J Nat Prod. 2025 Nov 3;88(11):2602–9. doi: 10.1021/acs.jnatprod.5c00926 (PMC12670701; doi:10.1021/acs.jnatprod.5c00926)
Supplement: Supplementary file 1 [file np5c00926_si_001.zip › NMR Data Dryinone A (1)/1H 13C HMBC/pdata/1/email_Sep24-2024_700_NB_84_1.pdf]

Broel PDR2 6a

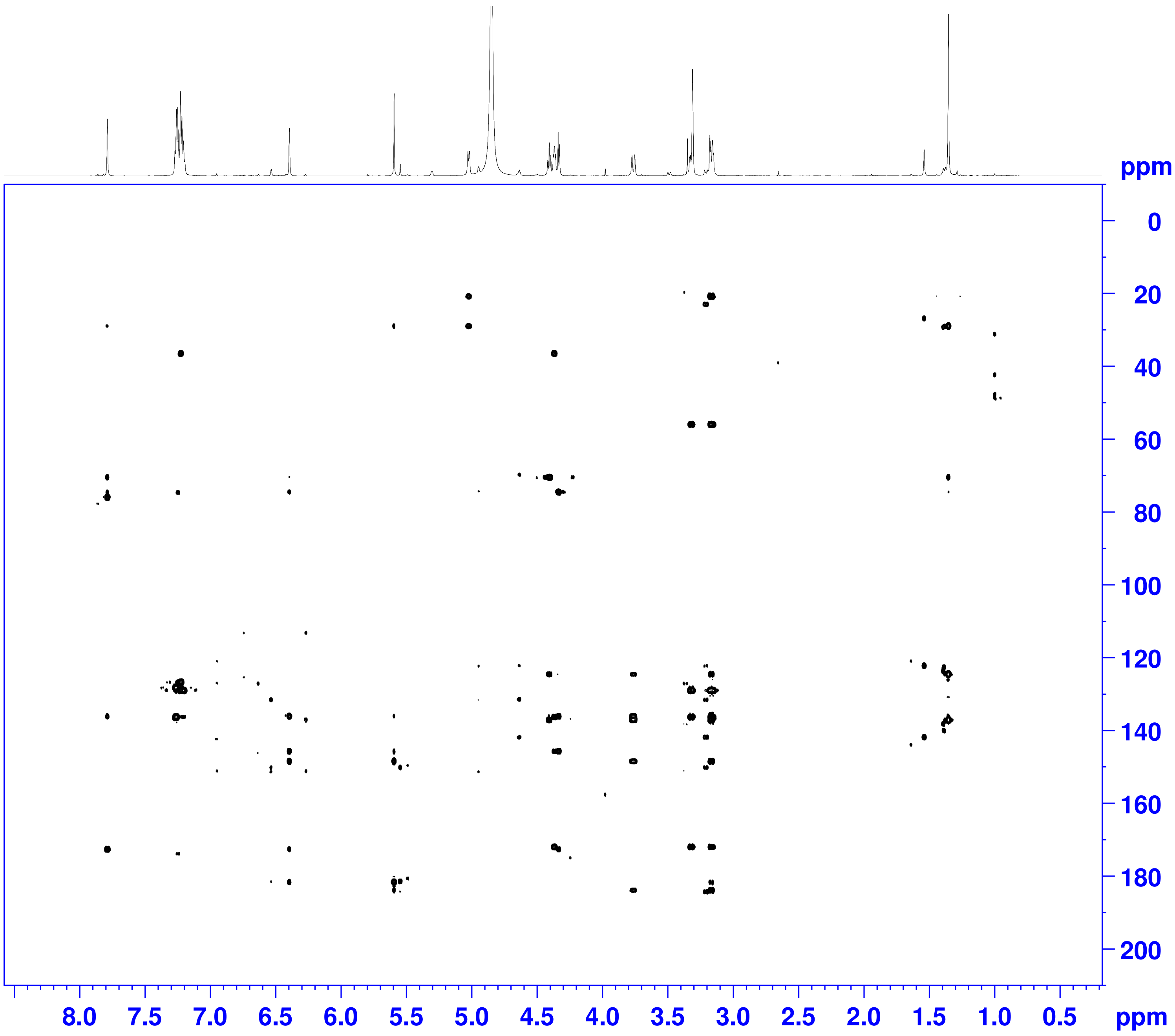


```
Current Data Parameters
NAME        Sep24-2024_700_NB
EXPNO                      84
PROCNO                      1

F2 - Acquisition Parameters
Date_                20240924
Time                  15.18 h
INSTRUM            Avance Neo
PROBHD       Z168794_0004 (
PULPROG         hmbcetgp13nd
TD                       4096
SOLVENT                  MeOD
NS                          4
DS                         16
SWH                  5882.353 Hz
FIDRES               2.872243 Hz
AQ                  0.3481600 sec
RG                        101
DW                     85.000 usec
DE                      10.00 usec
TE                      303.0 K
CNST6             120.0000000
CNST7             170.0000000
CNST13              8.0000000
CNST30              0.5981152
D0                 0.00000300 sec
D1                 2.00000000 sec
D6                 0.06250000 sec
D16                0.00020000 sec
IN0                0.00001291 sec
TDav                        1
SFO1              700.2830792 MHz
NUC1                       1H
P1                       7.98 usec
P2                      15.96 usec
PLW1              13.90999985 W
SFO2              176.1031546 MHz
NUC2                      13C
P3                      12.00 usec
P24                   2000.00 usec
PLW2             110.76999664 W
SPNAM[7]          Crp60comp.4
SPOAL7                  0.500
SPOFFS7                     0 Hz
SPW7              24.37100029 W
GPNAM[1]           SMSQ10.100
GPZ1                    80.00 %
GPNAM[3]           SMSQ10.100
GPZ3                    14.00 %
GPNAM[4]           SMSQ10.100
GPZ4                    -8.00 %
GPNAM[5]           SMSQ10.100
GPZ5                    -4.00 %
GPNAM[6]           SMSQ10.100
GPZ6                    -2.00 %
P16                   1000.00 usec

======== F1 INDIRECT DIMENSION ========
td1         256
sw_F1       220.000000

F1 - Acquisition parameters
TD                        256
SFO1                 176.1032 MHz
FIDRES             302.677307 Hz
SW                    220.000 ppm
FnMODE           Echo-Antiecho

F2 - Processing parameters
SI                       4096
SF                700.2800138 MHz
WDW                     QSINE
SSB                         4
LB                          0 Hz
GB                          0
PC                       1.40

F1 - Processing parameters
SI                       1024
MC2               echo-antiecho
SF                176.0855461 MHz
WDW                     QSINE
SSB                         2
LB                          0 Hz
GB                          0
```
