# Supplementary figures and images for: Dryinones: Structure Elucidation of Red Colorants from Submerged Cultures of Pleurotus dryinus
Source: J Nat Prod. 2025 Nov 3;88(11):2602–9. doi: 10.1021/acs.jnatprod.5c00926 (PMC12670701; doi:10.1021/acs.jnatprod.5c00926)

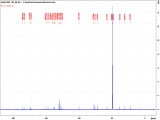

Supplement: Supplementary file 1 [file np5c00926_si_001.zip › NMR Data Dryinone A (1)/13C/pdata/1/thumb.png]

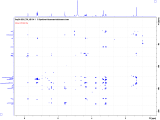

Supplement: Supplementary file 1 [file np5c00926_si_001.zip › NMR Data Dryinone A (1)/1H 13C HMBC/pdata/1/thumb.png]

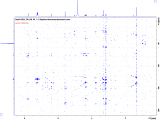

Supplement: Supplementary file 1 [file np5c00926_si_001.zip › NMR Data Dryinone A (1)/1H 13C HMBC 25NUS/pdata/1/thumb.png]

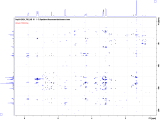

Supplement: Supplementary file 1 [file np5c00926_si_001.zip › NMR Data Dryinone A (1)/1H 13C HMBC 50NUS/pdata/1/thumb.png]

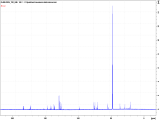

Supplement: Supplementary file 2 [file np5c00926_si_002.zip › NMR Data Dryinone B (2)/13C/pdata/1/thumb.png]

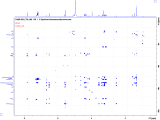

Supplement: Supplementary file 2 [file np5c00926_si_002.zip › NMR Data Dryinone B (2)/1H 13C HMBC/pdata/1/thumb.png]

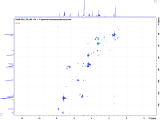

Supplement: Supplementary file 2 [file np5c00926_si_002.zip › NMR Data Dryinone B (2)/1H 13C HSQC/pdata/1/thumb.png]

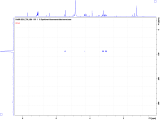

Supplement: Supplementary file 2 [file np5c00926_si_002.zip › NMR Data Dryinone B (2)/1H 15N HMBC/pdata/1/thumb.png]
