# Supplementary material for: Dryinones: Structure Elucidation of Red Colorants from Submerged Cultures of Pleurotus dryinus
Source: J Nat Prod. 2025 Nov 3;88(11):2602–9. doi: 10.1021/acs.jnatprod.5c00926 (PMC12670701; doi:10.1021/acs.jnatprod.5c00926)
Supplement: Supplementary file 2 [file np5c00926_si_002.zip › NMR Data Dryinone B (2)/13C/pdata/1/email_Oct08-2024_700_NBr_106_1.pdf]

Broel

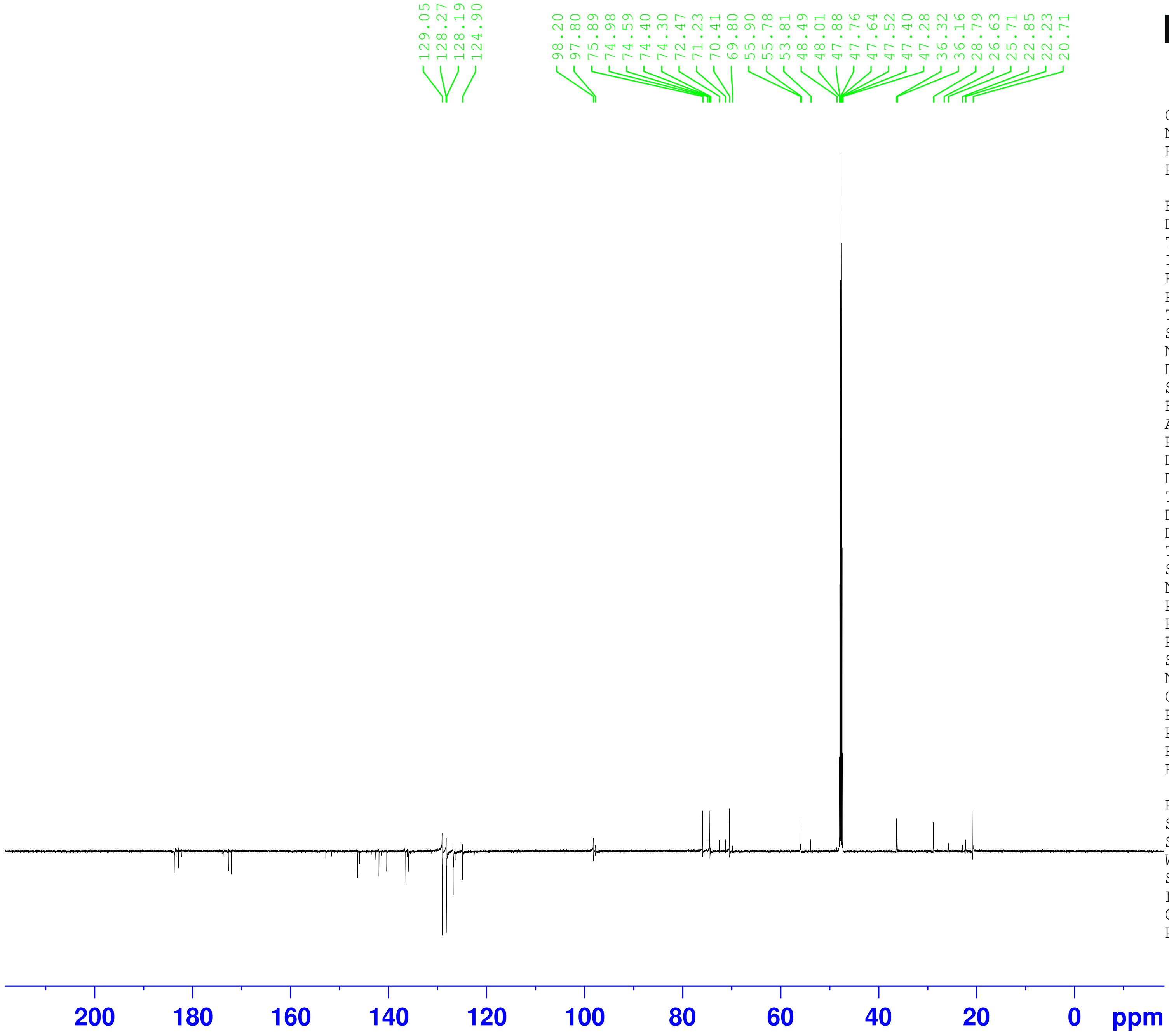

Current Data Parameters
NAME Oct08-2024_700_NBr
EXPNO 106
PROCNO 1

F2 - Acquisition Parameters
Date_ 20241008
Time 16.25 h
INSTRUM Avance Neo
PROBHD Z168794_0004 (
PULPROG zgpg30
TD 65536
SOLVENT MeOD
NS 1024
DS 4
SWH 41666.668 Hz
FIDRES 1.271566 Hz
AQ 0.7864320 sec
RG 101
DW 12.000 usec
DE 18.00 usec
TE 293.0 K
D1 2.00000000 sec
D11 0.03000000 sec
TD0 1
SFO1 176.1031546 MHz
NUC1 13C
P0 4.00 usec
P1 12.00 usec
PLW1 110.76999664 W
SFO2 700.2828011 MHz
NUC2 1H
CPDPRG[2 waltz65
PCPD2 80.00 usec
PLW2 13.90999985 W
PLW12 0.13841000 W
PLW13 0.06950700 W

F2 - Processing parameters
SI 32768
SF 176.0855461 MHz
WDW EM
SSB 0
LB 1.00 Hz
GB 0
PC 1.40
