# Supplementary material for: Dryinones: Structure Elucidation of Red Colorants from Submerged Cultures of Pleurotus dryinus
Source: J Nat Prod. 2025 Nov 3;88(11):2602–9. doi: 10.1021/acs.jnatprod.5c00926 (PMC12670701; doi:10.1021/acs.jnatprod.5c00926)
Supplement: Supplementary file 2 [file np5c00926_si_002.zip › NMR Data Dryinone B (2)/1H/pdata/1/email_Oct08-2024_700_NBr_90_1.pdf]

Broel

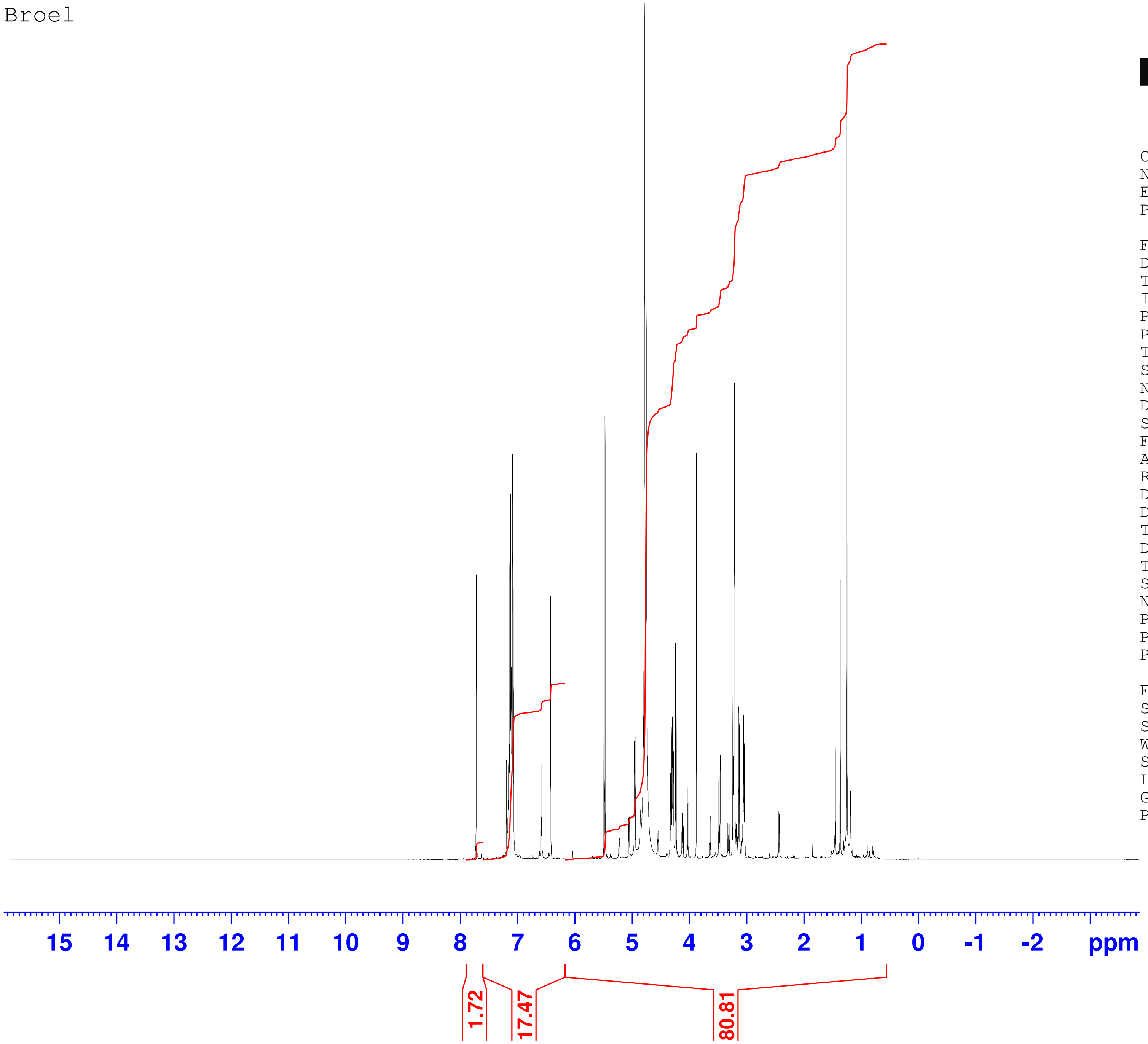

Current Data Parameters
NAME Oct08-2024_700_NBr
EXPNO 90
PROCNO 1

F2 - Acquisition Parameters
Date_ 20241008
Time 12.50 h
INSTRUM Avance Neo
PROBHD Z168794_0004 (
PULPROG zg30
TD 65536
SOLVENT MeOD
NS 64
DS 2
SWH 13888.889 Hz
FIDRES 0.423855 Hz
AQ 2.3592961 sec
RG 52
DW 36.000 usec
DE 12.14 usec
TE 303.0 K
D1 1.00000000 sec
TD0 1
SFO1 700.2843242 MHz
NUC1 1H
P0 2.66 usec
P1 7.98 usec
PLW1 13.90999985 W

F2 - Processing parameters
SI 65536
SF 700.2800833 MHz
WDW EM
SSB 0
LB 0.30 Hz
GB 0
PC 1.00
