# Supplementary material for: Dryinones: Structure Elucidation of Red Colorants from Submerged Cultures of Pleurotus dryinus
Source: J Nat Prod. 2025 Nov 3;88(11):2602–9. doi: 10.1021/acs.jnatprod.5c00926 (PMC12670701; doi:10.1021/acs.jnatprod.5c00926)
Supplement: Supplementary file 2 [file np5c00926_si_002.zip › NMR Data Dryinone B (2)/1H 13C HMBC/pdata/1/email_Oct08-2024_700_NBr_105_1.pdf]

Broel
PDR2_6b

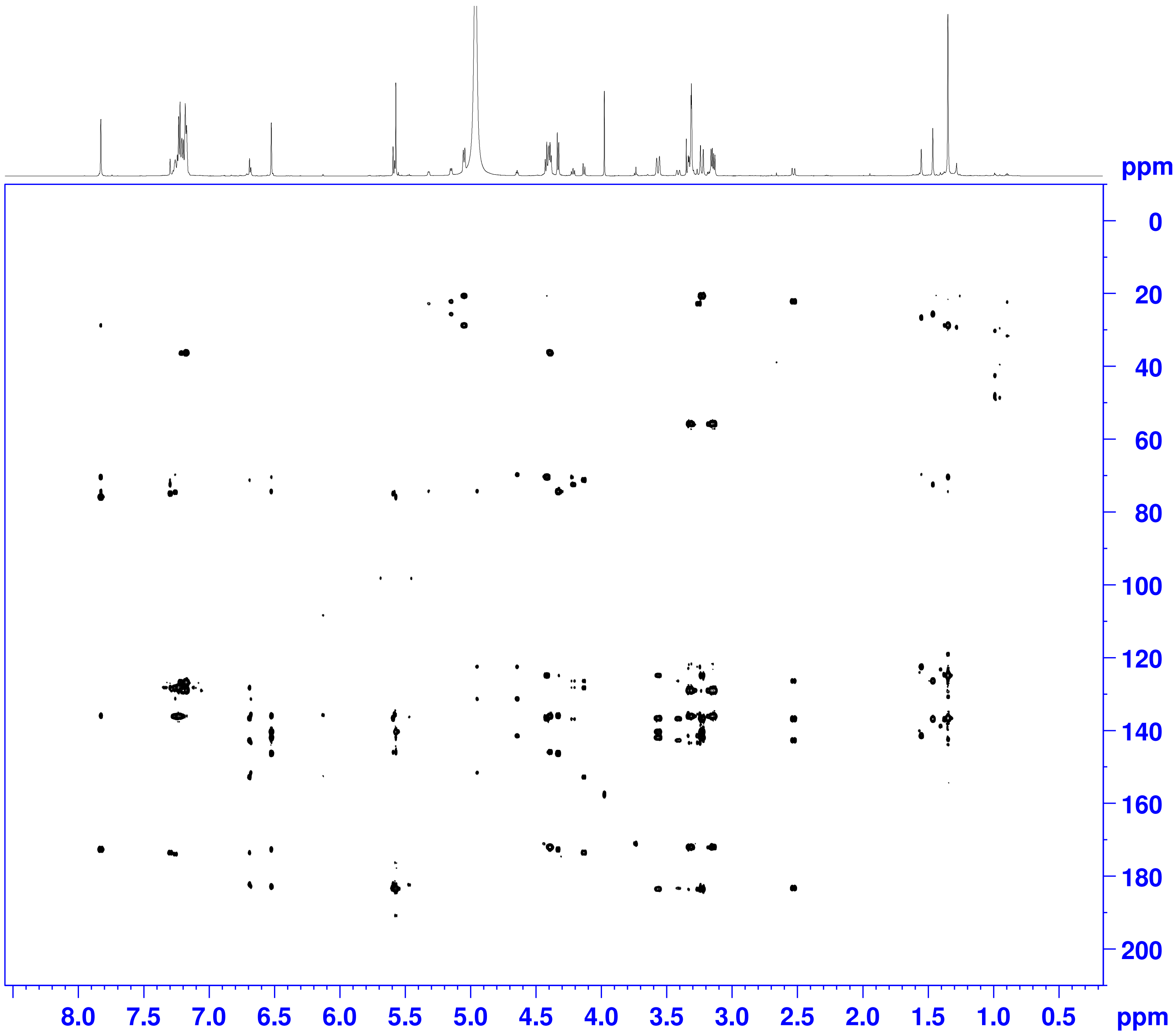

Current Data Parameters
NAME Oct08-2024_700_NBr
EXPNO 105
PROCNO 1

F2 - Acquisition Parameters
Date_ 20241008
Time 15.36 h
INSTRUM Avance Neo
PROBHD Z168794_0004 (
PULPROG hmbcetgpl3nd
TD 4096
SOLVENT MeOD
NS 4
DS 16
SWH 5882.353 Hz
FIDRES 2.872243 Hz
AQ 0.3481600 sec
RG 101
DW 85.000 usec
DE 10.00 usec
TE 293.0 K
CNST6 120.0000000
CNST7 170.0000000
CNST13 8.0000000
CNST30 0.5981152
D0 0.00000300 sec
D1 2.00000000 sec
D6 0.06250000 sec
D16 0.00020000 sec
IN0 0.00001291 sec
TDav 1
SFO1 700.2830679 MHz
NUC1 1H
P1 7.98 usec
P2 15.96 usec
PLW1 13.90999985 W
SFO2 176.1031546 MHz
NUC2 13C
P3 12.00 usec
P24 2000.00 usec
PLW2 110.76999664 W
SPNAM[7] Crp60comp.4
SPOAL7 0.500
SPOFFS7 0 Hz
SPW7 24.37100029 W
GPNAM[1] SMSQ10.100
GPZ1 80.00 %
GPNAM[3] SMSQ10.100
GPZ3 14.00 %
GPNAM[4] SMSQ10.100
GPZ4 -8.00 %
GPNAM[5] SMSQ10.100
GPZ5 -4.00 %
GPNAM[6] SMSQ10.100
GPZ6 -2.00 %
P16 1000.00 usec

======== F1 INDIRECT DIMENSION ========
td1 256
sw_F1 220.000000

F1 - Acquisition parameters
TD 256
SFO1 176.1032 MHz
FIDRES 302.677307 Hz
SW 220.000 ppm
FnMODE Echo-Antiecho

F2 - Processing parameters
SI 4096
SF 700.2800135 MHz
WDW QSINE
SSB 4
LB 0 Hz
GB 0
PC 1.40

F1 - Processing parameters
SI 1024
MC2 echo-antiecho
SF 176.0855461 MHz
WDW QSINE
SSB 2
LB 0 Hz
GB 0
