# Supplementary material for: Dryinones: Structure Elucidation of Red Colorants from Submerged Cultures of Pleurotus dryinus
Source: J Nat Prod. 2025 Nov 3;88(11):2602–9. doi: 10.1021/acs.jnatprod.5c00926 (PMC12670701; doi:10.1021/acs.jnatprod.5c00926)
Supplement: Supplementary file 2 [file np5c00926_si_002.zip › NMR Data Dryinone B (2)/1H 15N HMBC/pdata/1/email_Oct08-2024_700_NBr_103_1.pdf]

Broel

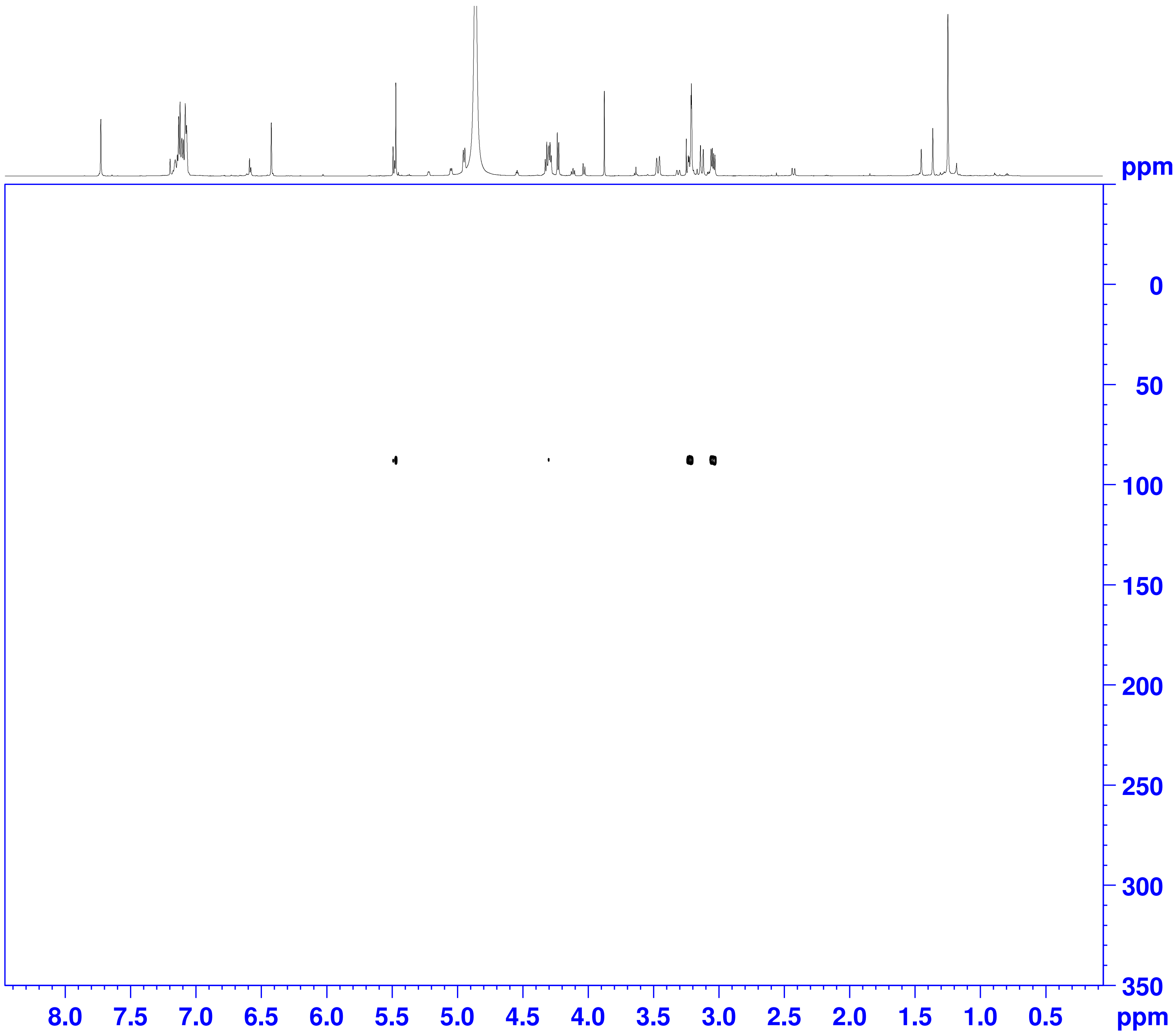

Current Data Parameters

NAME	Oct08-2024_700_NBr	
EXPNO	103	
PROCNO	1	

F2 - Acquisition Parameters

Date_	20241008	
Time	14.15	h
INSTRUM	Avance Neo	
PROBHD	Z168794_0004 (	
PULPROG	hmbcgpndqf	
TD	4096	
SOLVENT	MeOD	
NS	8	
DS	16	
SWH	5882.353	Hz
FIDRES	2.872243	Hz
AQ	0.3481600	sec
RG	101	
DW	85.000	usec
DE	10.00	usec
TE	293.0	K
CNST13	5.0000000	
D0	0.00000300	sec
D1	2.00000000	sec
D6	0.10000000	sec
D16	0.00020000	sec
IN0	0.00001761	sec
TDav	1	
SFO1	700.2830679	MHz
NUC1	1H	
P1	7.98	usec
P2	15.96	usec
PLW1	13.90999985	W
SFO2	70.9694000	MHz
NUC2	15N	
P3	32.00	usec
PLW2	210.44000244	W
GPNAM[1]	SMSQ10.100	
GPZ1	70.00	%
GPNAM[2]	SMSQ10.100	
GPZ2	30.00	%
GPNAM[3]	SMSQ10.100	
GPZ3	50.10	%
P16	1000.00	usec

======== F1 INDIRECT DIMENSION ========

td1	128
sw_F1	400.000000

F1 - Acquisition parameters

TD	128	
SFO1	70.9694	MHz
FIDRES	443.558746	Hz
SW	400.000	ppm
FnMODE	QF	

F2 - Processing parameters

SI	4096	
SF	700.2800839	MHz
WDW	QSINE	
SSB	0	
LB	0	Hz
GB	0	
PC	1.40	

F1 - Processing parameters

SI	1024	
MC2	QF	
SF	70.9587562	MHz
WDW	QSINE	
SSB	0	
LB	0	Hz
GB	0	
