# Supplementary material for: Dryinones: Structure Elucidation of Red Colorants from Submerged Cultures of Pleurotus dryinus
Source: J Nat Prod. 2025 Nov 3;88(11):2602–9. doi: 10.1021/acs.jnatprod.5c00926 (PMC12670701; doi:10.1021/acs.jnatprod.5c00926)
Supplement: Supplementary file 3 [file np5c00926_si_003.pdf]

SUPPORTING INFORMATION

Dryinones: Structure Elucidation of Red Colorants from Submerged Cultures of *Pleurotus dryinus*

Niklas Broel[1], Johanna V. Stein[1], Franziska V. Wengner[1], Marvin H.J. Domanski[2], Tatyana Zhuk[1,3], Martin Gand[1,*]

[1]Institute of Food Chemistry and Food Biotechnology, Justus Liebig University Giessen, Heinrich-Buff-Ring 17, 35392 Giessen, Germany

[2]Institute of Organic Chemistry, Justus Liebig University Giessen, Heinrich-Buff-Ring 17, 35392 Giessen, Germany

[3]Faculty of Chemical Technology, Igor Sikorsky Kyiv Polytechnic Institute, Beresteiskyi Ave 37, 03056 Kyiv, Ukraine

*Corresponding author: Martin Gand; E-Mail: Martin.Gand@lcb.chemie.uni-giessen.de, ORC-ID: 0000-0001-8211-691X.

SUPPORTING FIGUES AND TABLES

Figure S1	Phylogenetic analysis of *P. dryinus* and related species	Page 2
Figure S2	HPLC-DAD chromatogram of *P. dryinus* crude extract	Page 3
Figure S3	UV/vis spectra of **1** and **2**	Page 3
Figure S4	Prep. HPLC chromatogram of eluate 6, C18 phase	Page 4
Figure S5	Analy. HPLC chromatogram of **2** isomers	Page 5
Figure S6	Prep. HPLC chromatogram of **2**, phenyl-hexyl phase	Page 6
Figure S7	Prep. HPLC chromatogram of **1**, phenyl-hexyl phase	Page 7
Figure S8	HR-ESI(+)-MS of **1**	Page 8
Figure S9	HR-ESI(+)-MS/MS of **1**	Page 8
Figure S10	HR-ESI(+)-MS/MS of **2**	Page 8
Figure S11	^{1}H NMR (CD_3OD, 700 MHz) of **1**	Page 9
Figure S12	^{13}C NMR (CD_3OD, 176 MHz) of **1**	Page 10
Figure S13	*J*-modulated ^{13}C NMR (CD_3OD, 176 MHz) of **1**	Page 11
Figure S14	^{1}H-^{13}C HSQC NMR (CD_3OD, 700 MHz, 176 MHz) of **1**	Page 12
Figure S15	^{1}H-^{13}C HMBC NMR (CD_3OD, 700 MHz, 176 MHz) of **1**	Page 13
Figure S16	^{1}H-^{13}C HMBC NMR (CD_3OD, 700 MHz, 176 MHz, 25% NUS) of **1**	Page 14
Figure S17	^{1}H-^{13}C HMBC NMR (CD_3OD, 700 MHz, 176 MHz, 50% NUS) of **1**	Page 15
Figure S18	^{1}H-^{15}N HMBC NMR (CD_3OD, 700 MHz, 71 MHz) of **1**	Page 16
Figure S19	^{1}H-^{1}H COSY NMR (CD_3OD, 700 MHz) of **1**	Page 17
Figure S20	^{1}H-^{1}H NOESY NMR (CD_3OD, 700 MHz) of **1**	Page 18
Figure S21	^{1}H NMR (CD_3OD, 700 MHz) of **2**	Page 19
Figure S22	^{13}C NMR (CD_3OD, 176 MHz) of **2**	Page 20
Figure S23	*J*-modulated ^{13}C NMR (CD_3OD, 176 MHz) of **2**	Page 21
Figure S24	^{1}H-^{13}C HSQC NMR (CD_3OD, 700 MHz, 176 MHz) of **2**	Page 22
Figure S25	^{1}H-^{13}C HMBC NMR (CD_3OD, 700 MHz, 176 MHz) of **2**	Page 23
Figure S26	^{1}H-^{15}N HMBC NMR (CD_3OD, 700 MHz, 71 MHz) of **2**	Page 24
Figure S27	^{1}H-^{1}H COSY NMR (CD_3OD, 700 MHz) of **2**	Page 25
Figure S28	Detailed information, UV/vis and ECD of calcd. (6*R*,9*R*,10*R*,18*S*)-**1**	Page 26
Figure S29	Detailed information, UV/vis and ECD of calcd. (6*R*,9*R*,10*S*,18*S*)-**1**	Page 27
Figure S30	Detailed information, UV/vis and ECD of calcd. (6*R*,9*S*,10*R*,18*S*)-**1**	Page 28
Figure S31	Detailed information, UV/vis and ECD of calcd. (6*R*,9*S*,10*S*,18*S*)-**1**	Page 29
Figure S32	Detailed information, UV/vis and ECD of calcd. (6*S*,9*R*,10*R*,18*S*)-**1**	Page 30
Figure S33	Detailed information, UV/vis and ECD of calcd. (6*S*,9*R*,10*S*,18*S*)-**1**	Page 31
Figure S34	Detailed information, UV/vis and ECD of calcd. (6*S*,9*S*,10*R*,18*S*)-**1**	Page 32
Figure S35	Detailed information, UV/vis and ECD of calcd. (6*S*,9*S*,10*S*,18*S*)-**1**	Page 33
Figure S36	ECD spectra of **1** and its computed 18*S*-isomers	Page 34
Figure S37	Detailed information, UV/vis and ECD of calcd. (6*R*,9*R*,10*R*,18*S*)-**2**	Page 35
Figure S38	IR spectrum of **1**	Page 36
Figure S39	IR spectrum of **2**	Page 37
Figure S40	ECD spectrum of **2**	Page 38

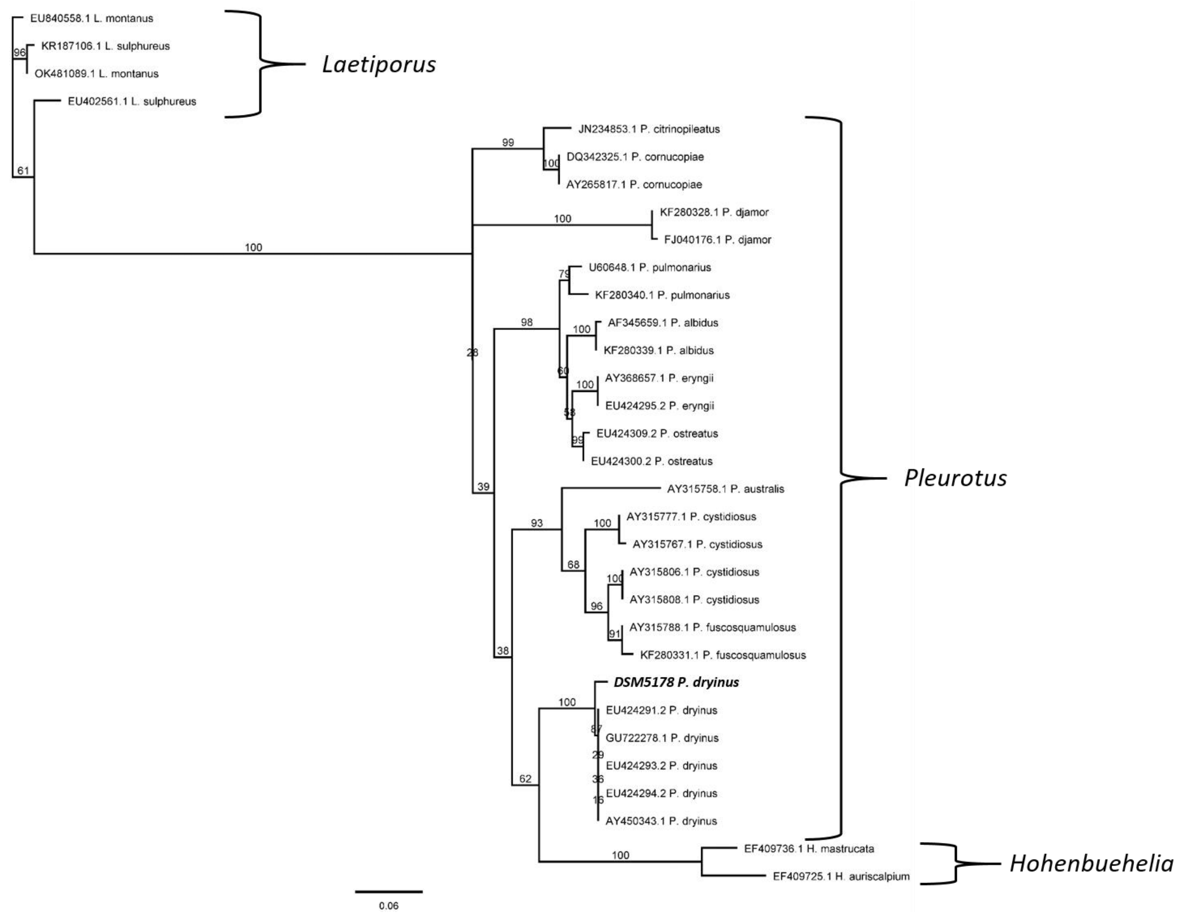

Figure S1: Phylogenetic analysis of ITS regions from members of the *Pleurotus* and *Hohenbuehelia* family from GenBank. The alignment type was global with gaps open penalty of 13 and gap extension penalty of 3 using the cost matrix 65%. The phylogenetic tree was calculated using Geneious v.9.1 by maximum likelihood inference[36] using the PhyML plugin, substation model JC69[37] calculated with 1000 bootstraps, numbers at the branches are confidence values, all other parameters were set as default.

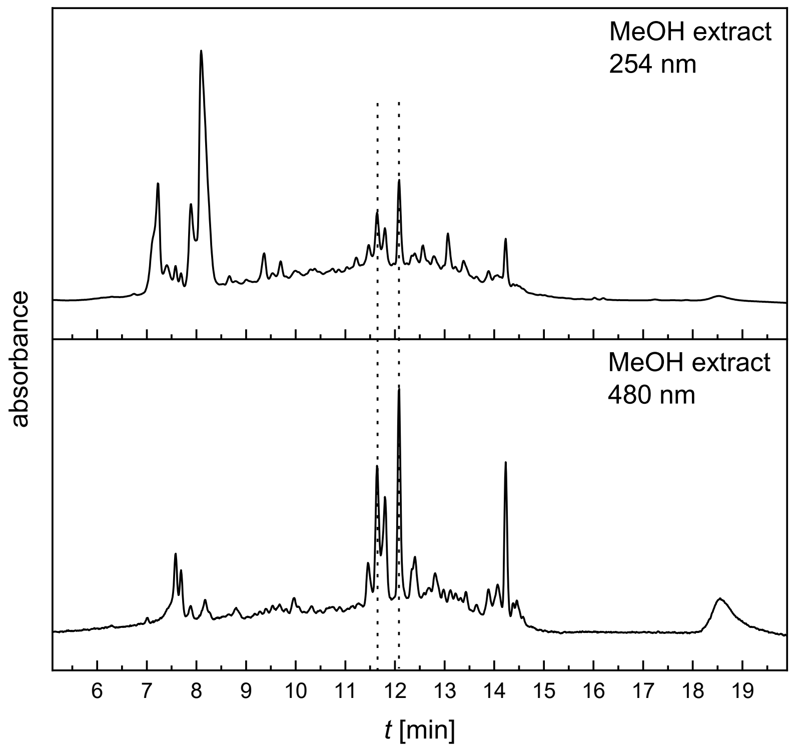

Figure S2: HPLC-DAD chromatograms of *P. dryinus* crude methanol extract measured at 254 nm (top) and 480 nm (bottom). The two main signals were marked with dashed lines.

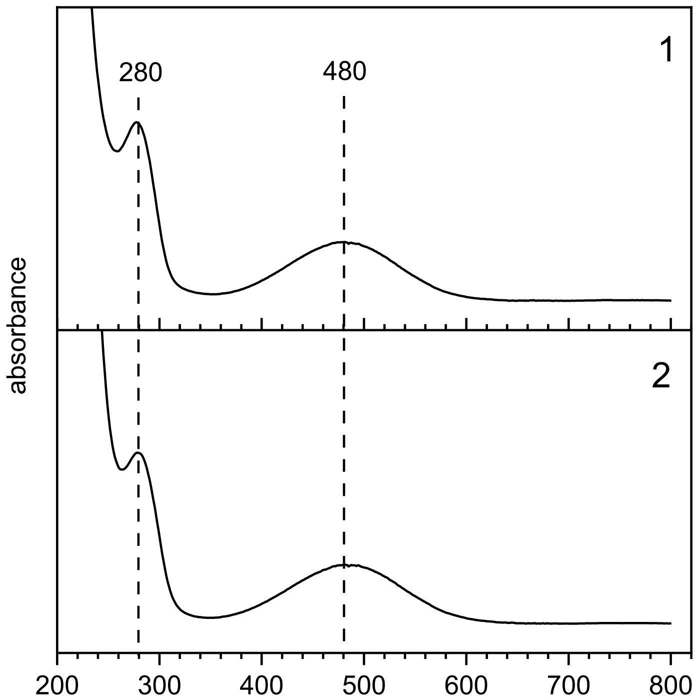

Figure S3: UV/vis spectra of the compounds **1** and **2**.

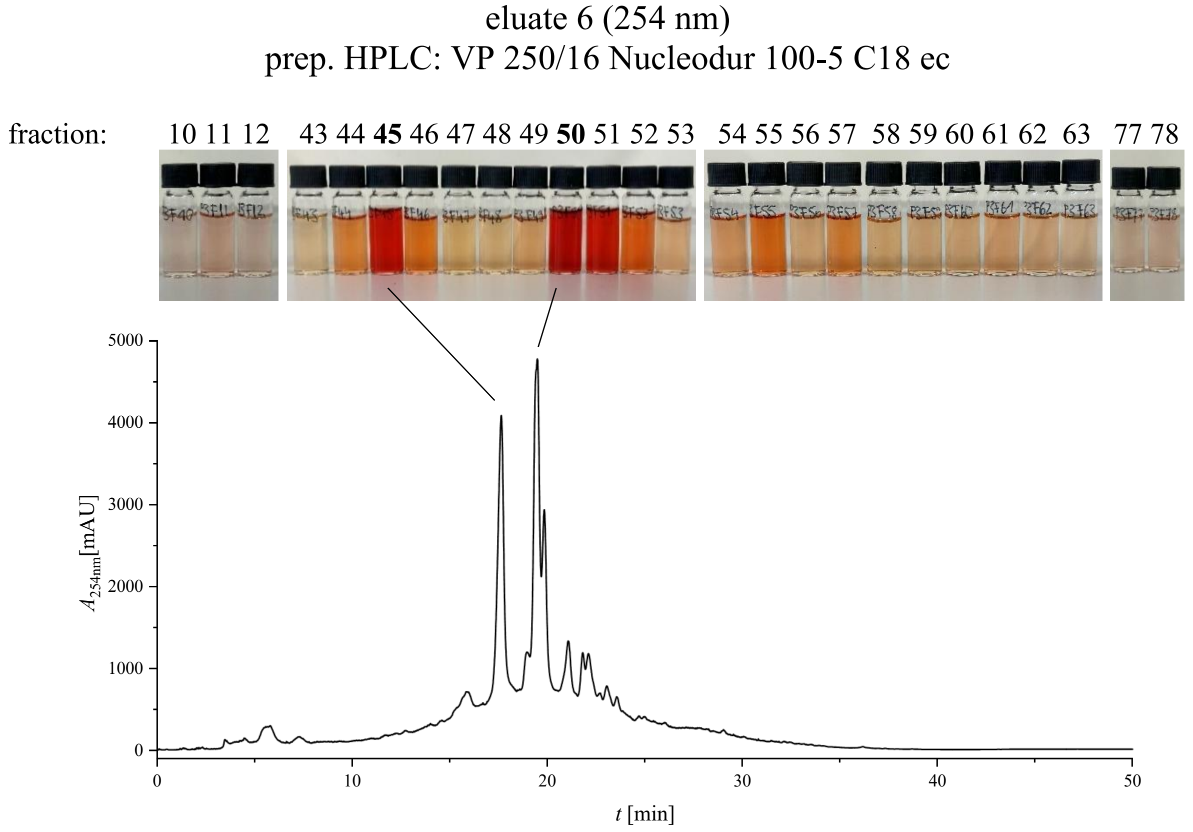

Figure S4: Chromatogram of the preparative HPLC separation of eluate 6 using a C18 phase.

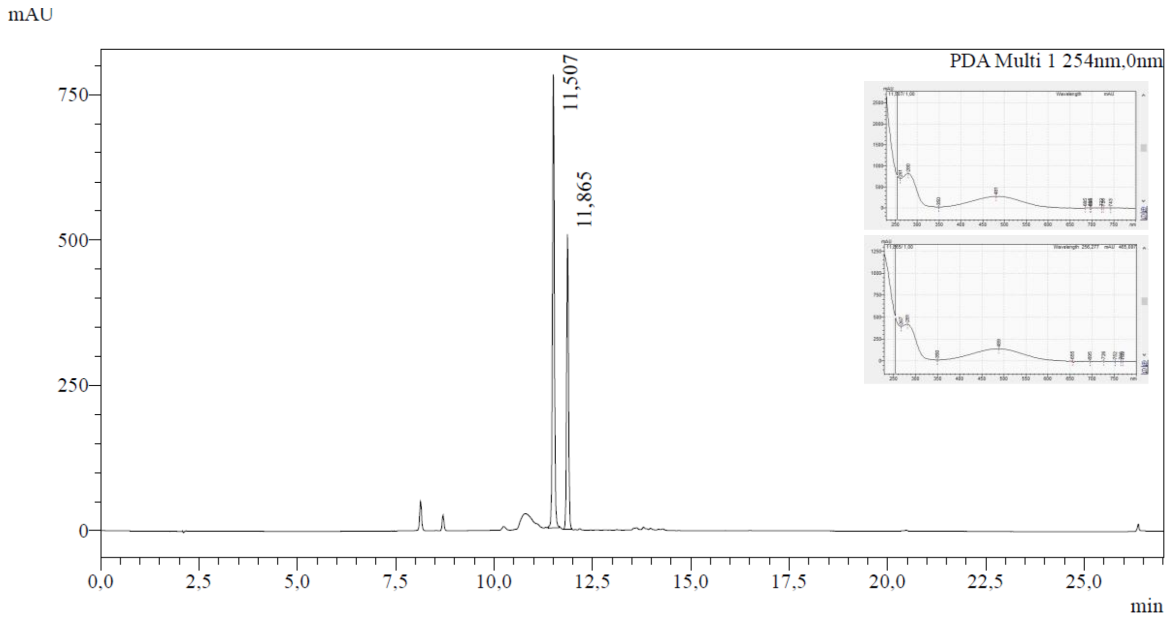

PDA Ch1 254nm

Peak#	Ret. Time	Area	Height
1	11,507	2694789	780002
2	11,865	1725790	507355

Figure S5: Analytical HPLC chromatogram of preparative HPLC fractions containing **2** exhibiting two main signals with their t_R as well es their peak areas using a phenyl phase.

2 (254 nm)
prep. HPLC: VP 250/21 Nucleodur Phenyl-Hexyl, 5 µm

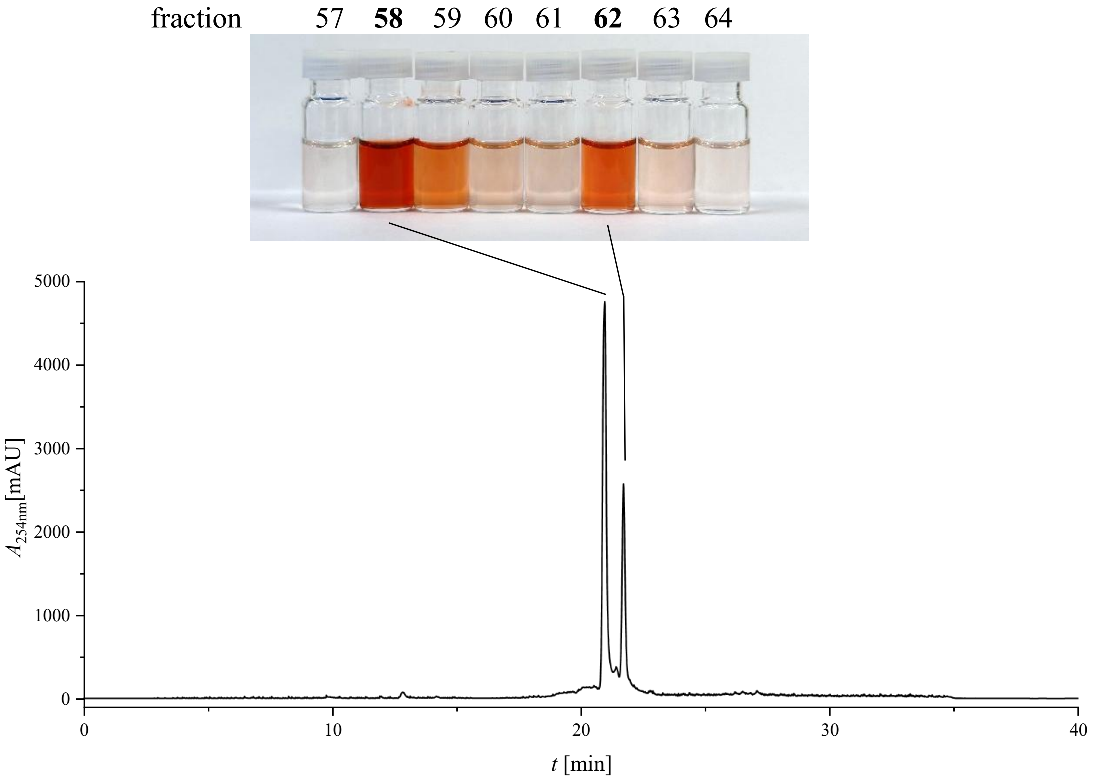

Figure S6: Chromatogram of the preparative HPLC separation of C18 prep. fractions containing **2** using a phenyl-hexyl phase.

1 (254 nm)
prep. HPLC: VP 250/21 Nucleodur Phenyl-Hexyl, 5 µm

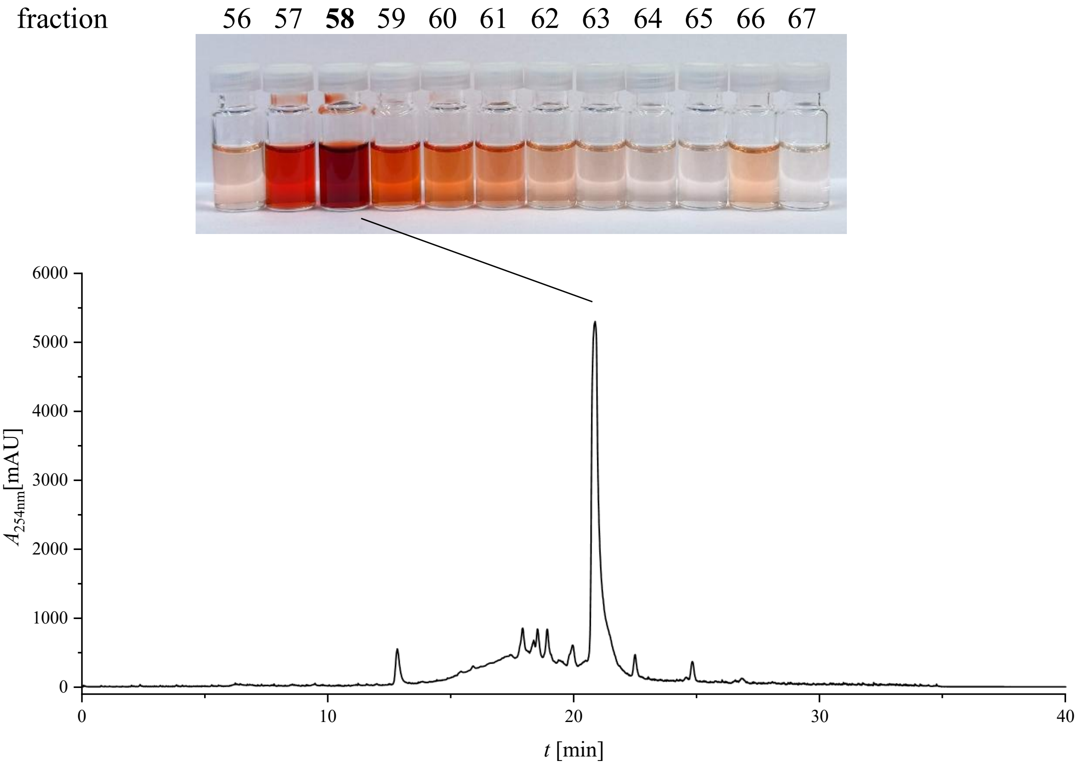

Figure S7: Chromatogram of the preparative HPLC separation of C18 prep. fractions containing **1** using a phenyl-hexyl phase.

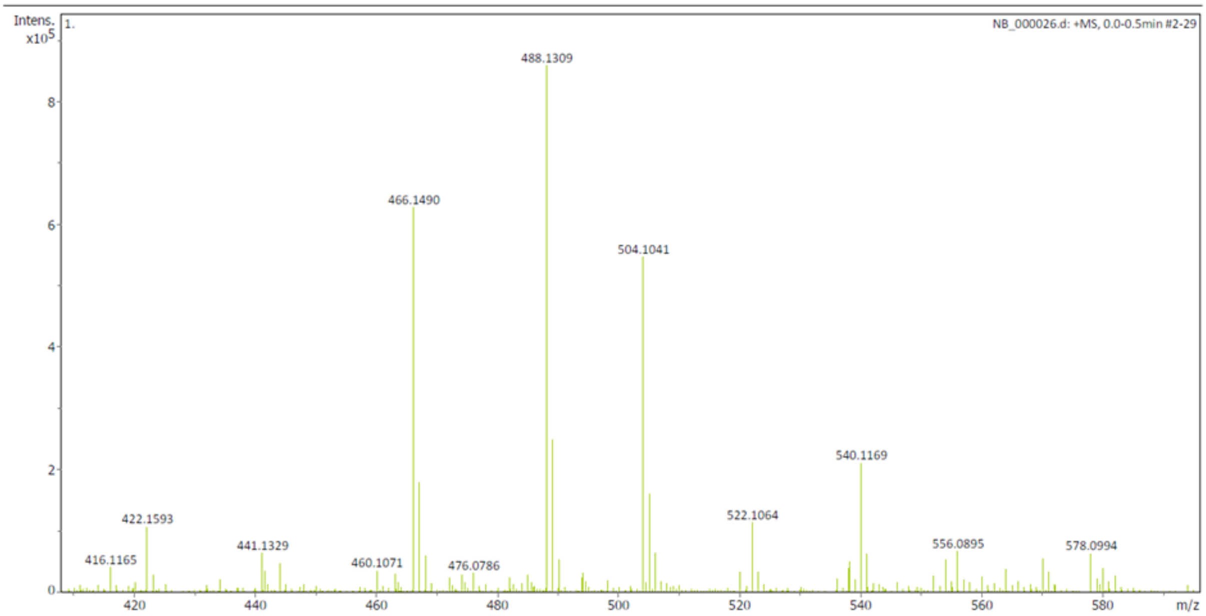

Figure S8: HR-ESI(+)-MS spectrum of **1**.

1

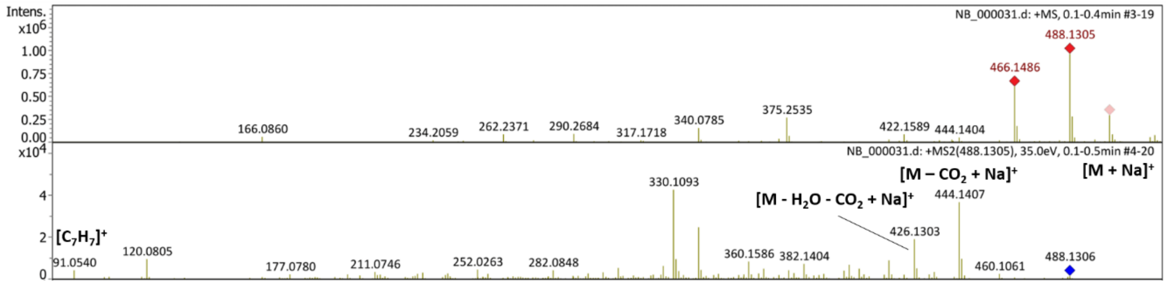

Figure S9: HR-ESI(+)-MS/MS of **1**.

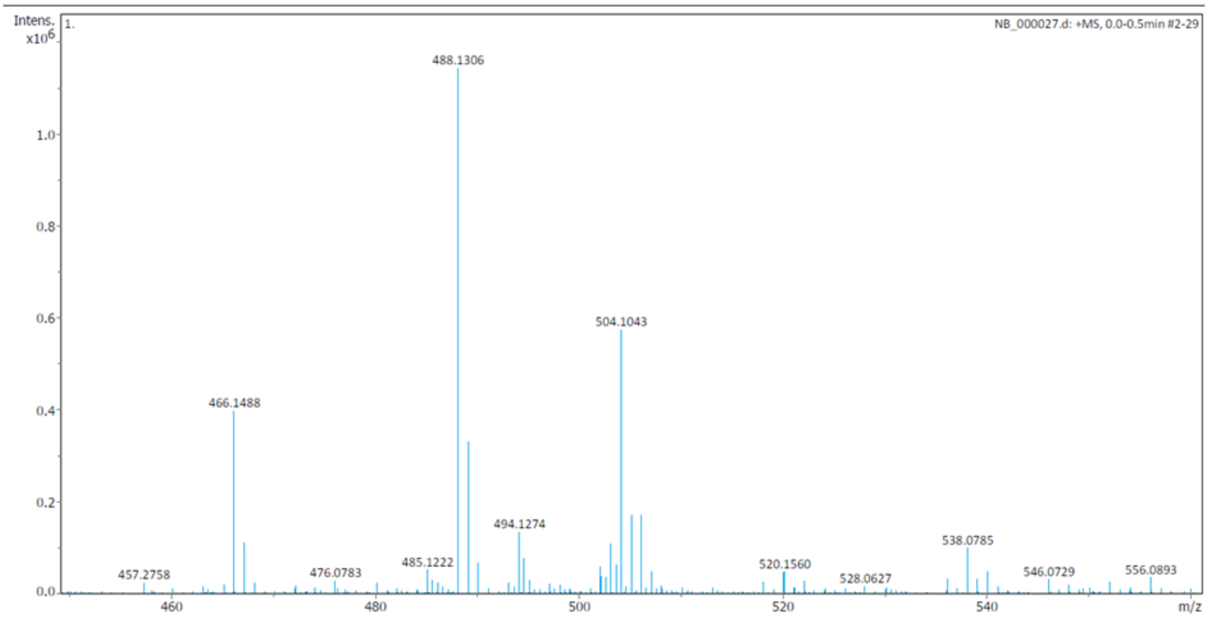

Figure S10: HR-ESI(+)-MS of **2**.

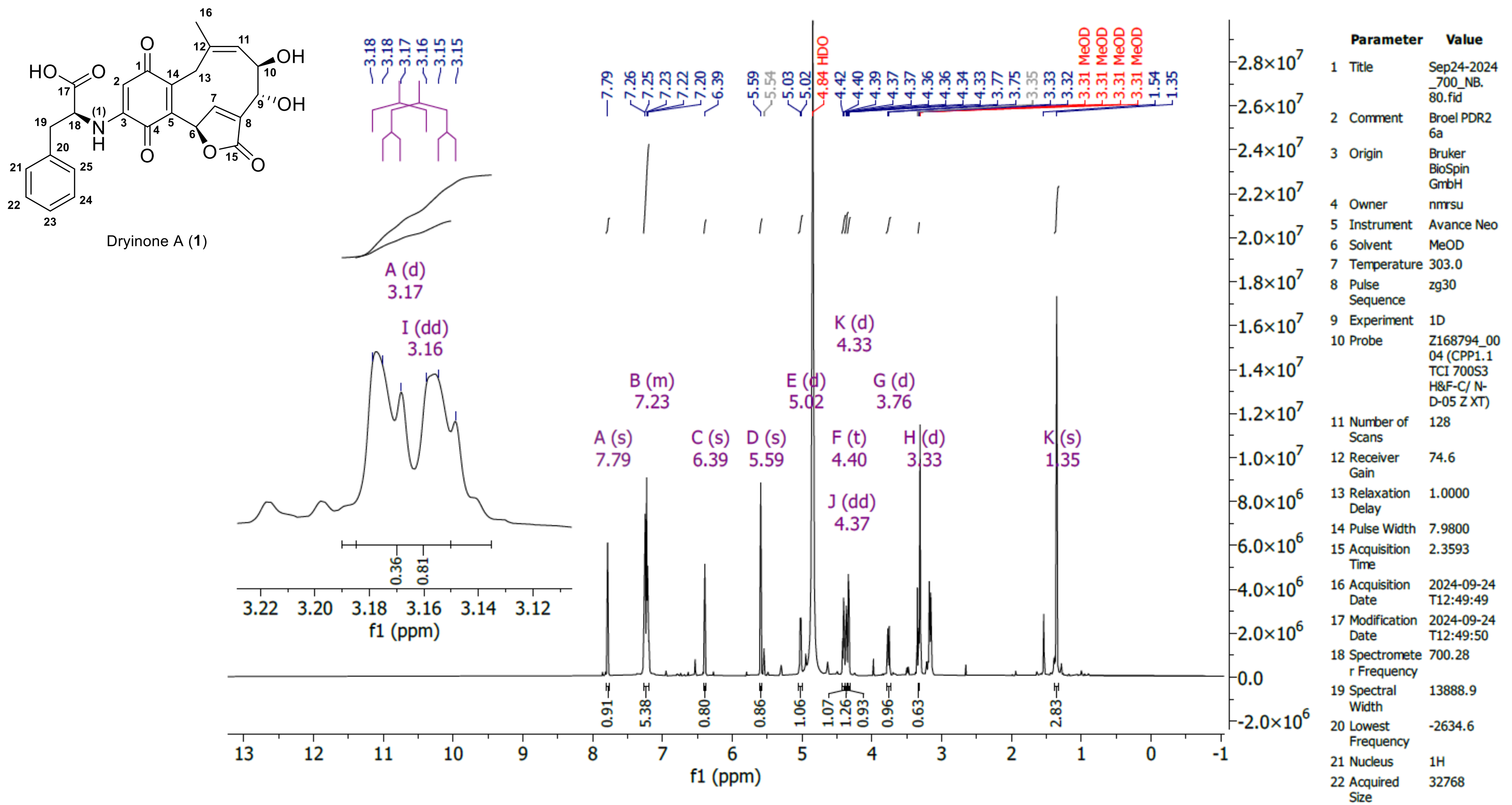

^{1}H NMR (MeOD, 700 MHz) δ 7.79 (1H, s), 7.26 – 7.20 (5H, m), 6.39 (1H, s), 5.59 (1H, s), 5.02 (1H, d, *J* = 8.6 Hz), 4.40 (1H, t, *J* = 8.3 Hz), 4.37 (1H, dd, *J* = 7.7, 5.0 Hz), 4.33 (1H, d, *J* = 8.1 Hz), 3.76 (1H, d, *J* = 14.4 Hz), 3.33 (1H, d, *J* = 5.1 Hz), 3.17 (1H, d, *J* = 13.9 Hz), 3.16 (1H, dd, *J* = 14.2, 4.5 Hz), 1.35 (3H, s)

Figure S11: ^{1}H NMR (CD_3OD, 700 MHz) spectrum of **1**.

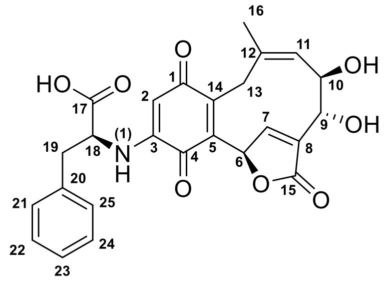

Dryinone A (**1**)

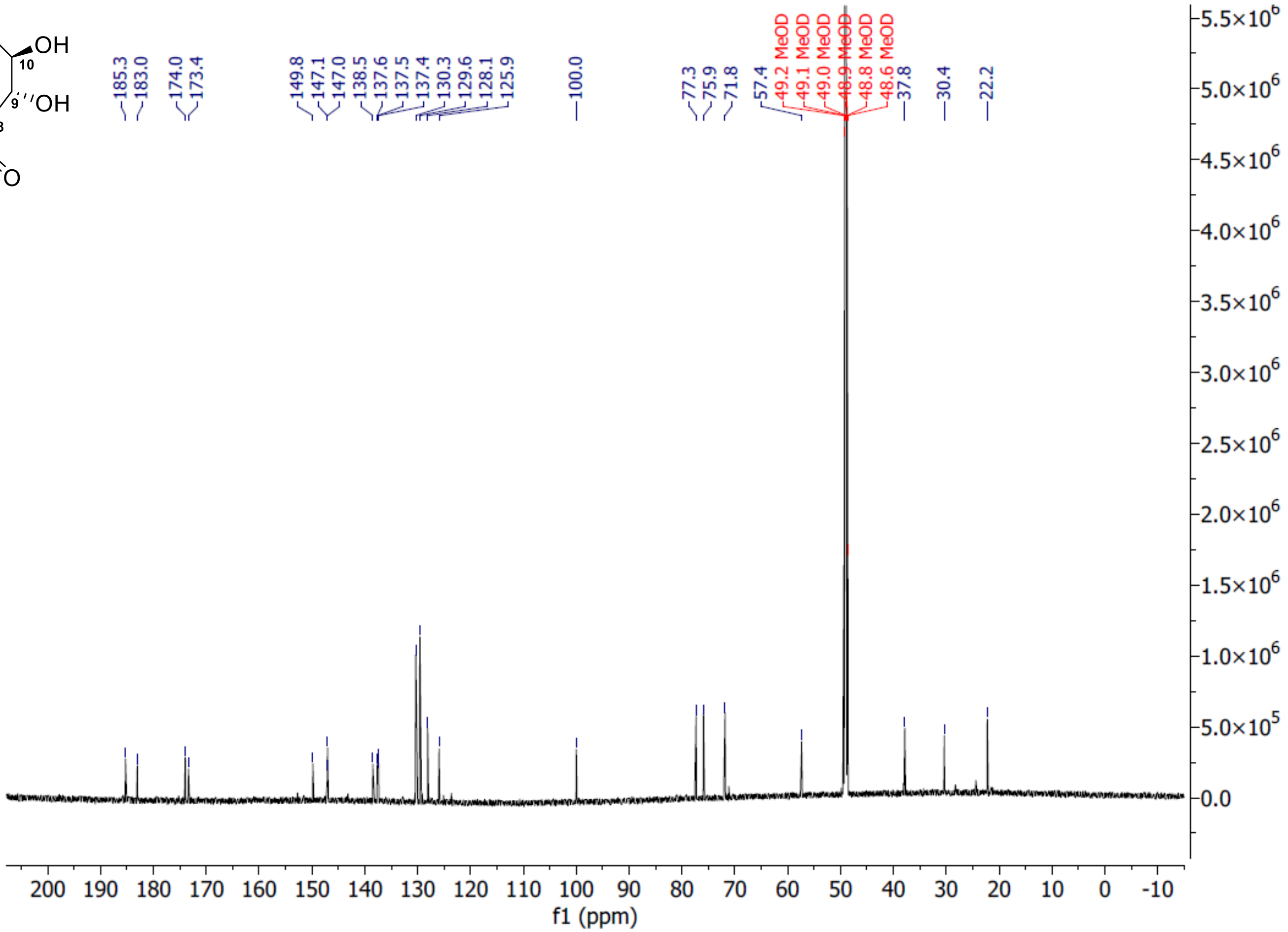

	Parameter	Value
1	Title	Sep24-2024_700_NB.86.fid
2	Comment	Broel PDR2 6a
3	Origin	Bruker BioSpin GmbH
4	Owner	nmrsu
5	Instrument	Avance Neo
6	Solvent	MeOD
7	Temperature	303.0
8	Pulse Sequence	zgpg30
9	Experiment	1D
10	Probe	Z168794_0004 (CPP1.1 TCI 700S3 H&F-C/ N-D-05 Z XT)
11	Number of Scans	1024
12	Receiver Gain	101.0
13	Relaxation Delay	2.0000
14	Pulse Width	12.0000
15	Acquisition Time	0.7864
16	Acquisition Date	2024-09-24T17:23:41
17	Modification Date	2024-09-24T17:23:42
18	Spectrometer Frequency	176.10
19	Spectral Width	41666.7
20	Lowest Frequency	-2982.6
21	Nucleus	13C
22	Acquired Size	32768
23	Spectral Size	65536
24	Digital Resolution	0.64

^{13}C NMR (MeOD, 176 MHz) δ 185.3, 183.0, 174.0, 173.4, 149.8, 147.1, 147.0, 138.5, 137.6, 137.5, 137.4, 130.3, 129.6, 128.1, 125.9, 100.0, 77.3, 75.9, 71.8, 57.4, 37.8, 30.4, 22.2.

Figure S 12: ^{13}C NMR (CD_3OD, 176 MHz) spectrum of **1**.

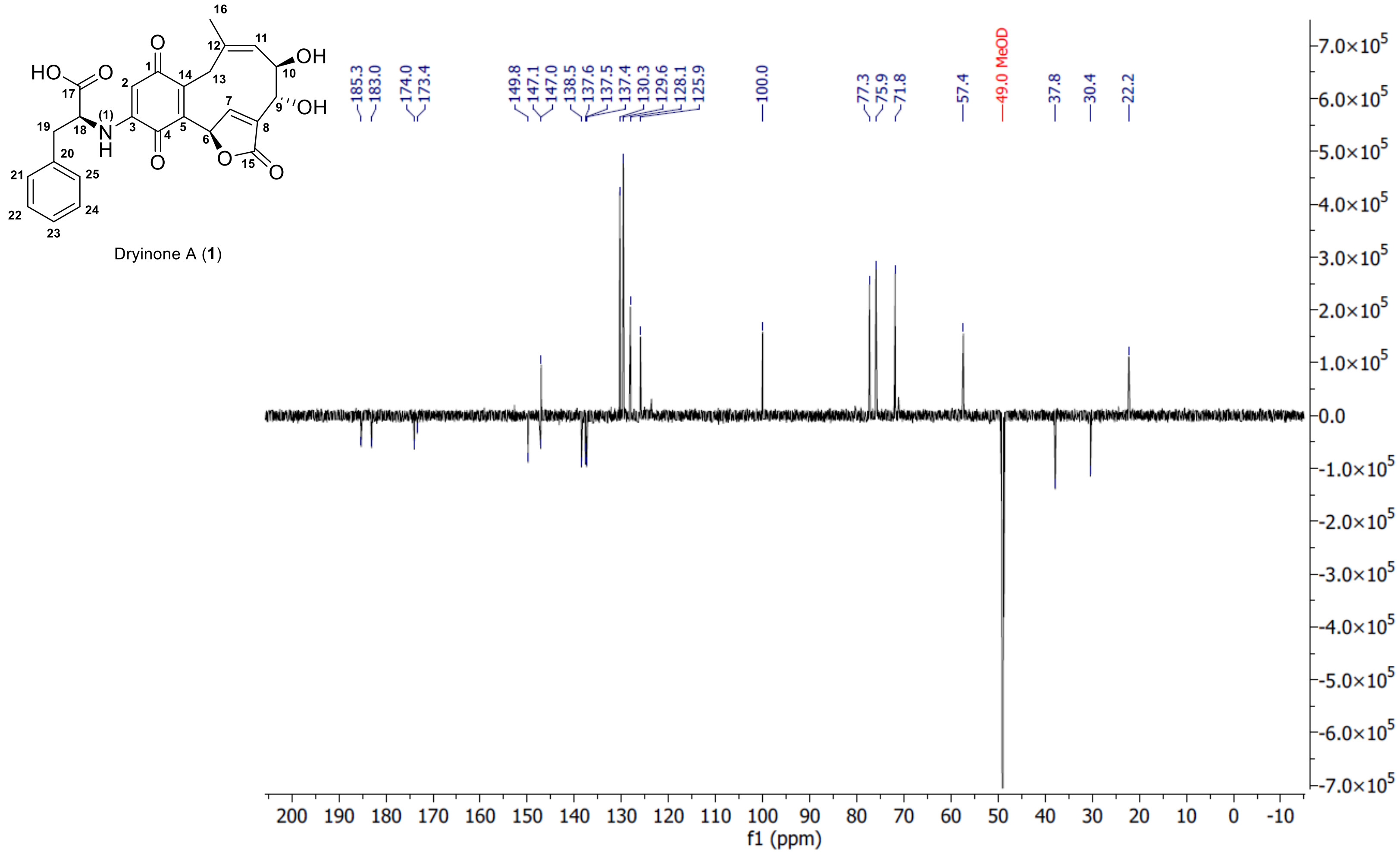

	Parameter	Value
1	Title	Sep24-2024_700_NB.87.fid
2	Comment	Broel PDR2 6a
3	Origin	Bruker BioSpin GmbH
4	Owner	nmrsu
5	Instrument	Avance Neo
6	Solvent	MeOD
7	Temperature	303.0
8	Pulse Sequence	jmod
9	Experiment	JMOD
10	Probe	Z168794_0004 (CPP1.1 TCI 700S3 H&F-C/ N-D-05 Z XT)
11	Number of Scans	256
12	Receiver Gain	101.0
13	Relaxation Delay	2.0000
14	Pulse Width	12.0000
15	Acquisition Time	0.7864
16	Acquisition Date	2024-09-24T18:21:02
17	Modification Date	2024-09-24T18:21:04
18	Spectrometer Frequency	176.10
19	Spectral Width	41666.7
20	Lowest Frequency	-2982.5
21	Nucleus	13C
22	Acquired Size	32768
23	Spectral Size	65536
24	Digital Resolution	0.64

^{13}C NMR (MeOD, 176 MHz) δ 185.3, 183.0, 174.0, 173.4, 149.8, 147.1, 147.0, 138.5, 137.6, 137.5, 137.4, 130.3, 129.6, 128.1, 125.9, 100.0, 77.3, 75.9, 71.8, 57.4, 37.8, 30.4, 22.2.

Figure S13: *J*-modulated ^{13}C NMR (CD_3OD, 176 MHz) spectrum of **1.**

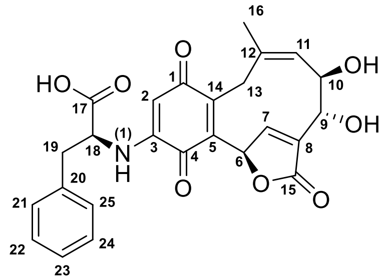

Dryinone A (**1**)

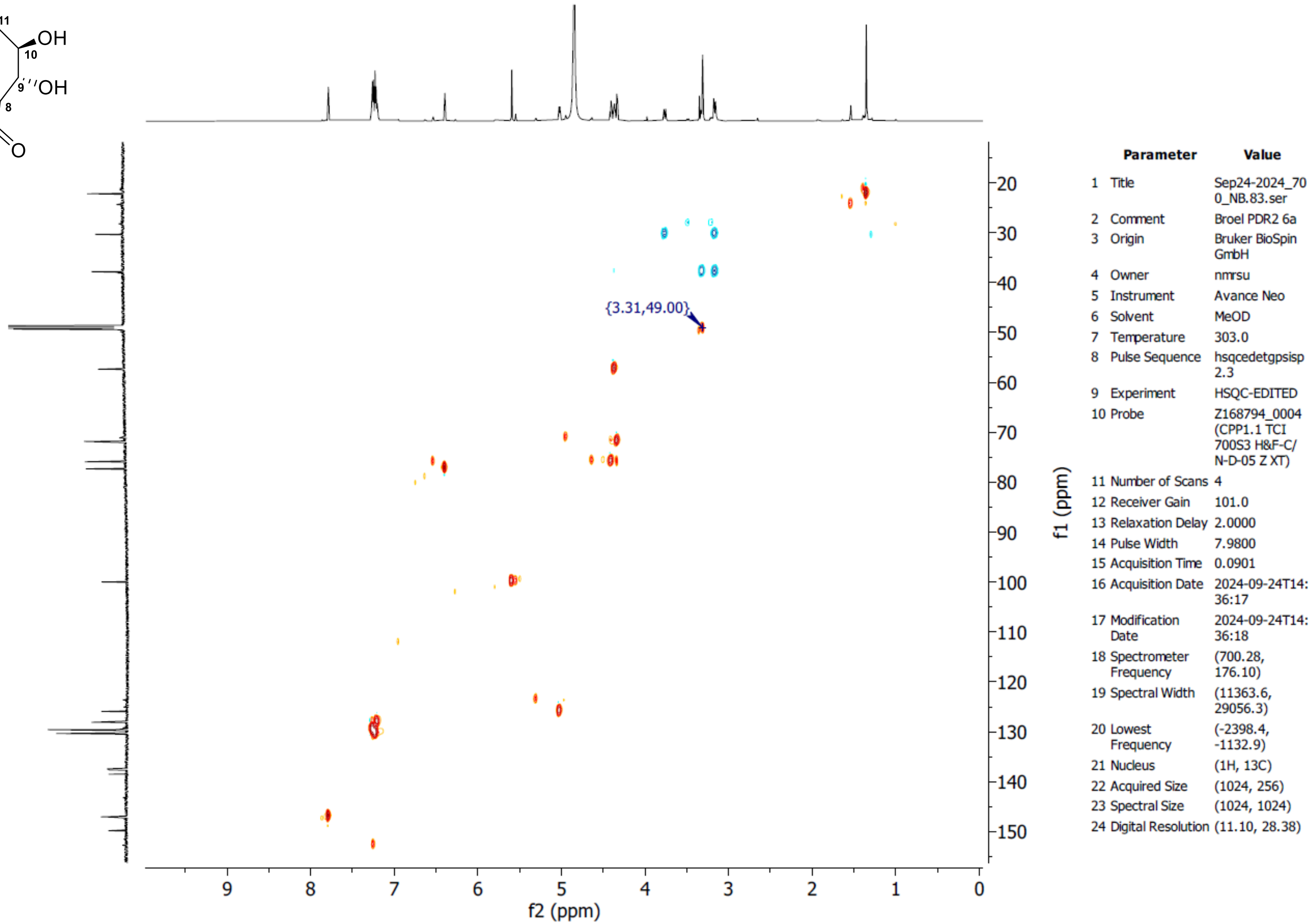

Figure S 14: ^{1}H-^{13}C HSQC NMR (CD_3OD, 700 MHz, 176 MHz) spectrum of **1**.

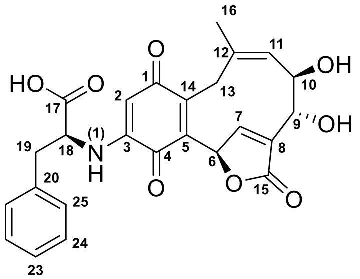

Dryinone A (**1**)

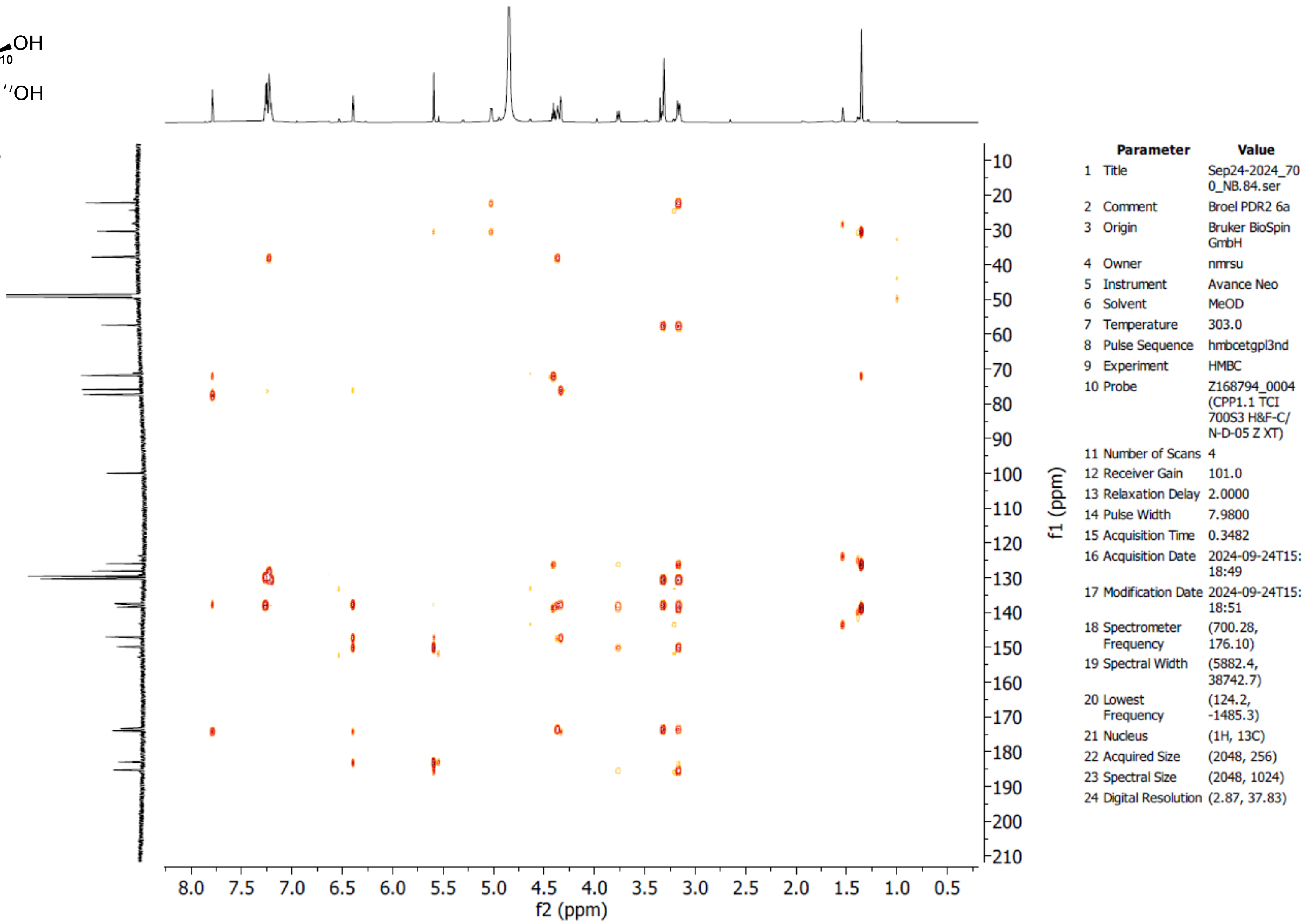

Figure S15: ^{1}H-^{13}C HMBC NMR (CD_3OD, 700 MHz, 176 MHz) spectrum of **1**.

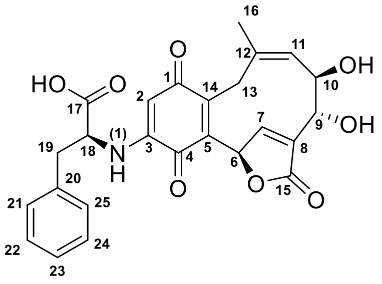

Dryinone A (**1**)

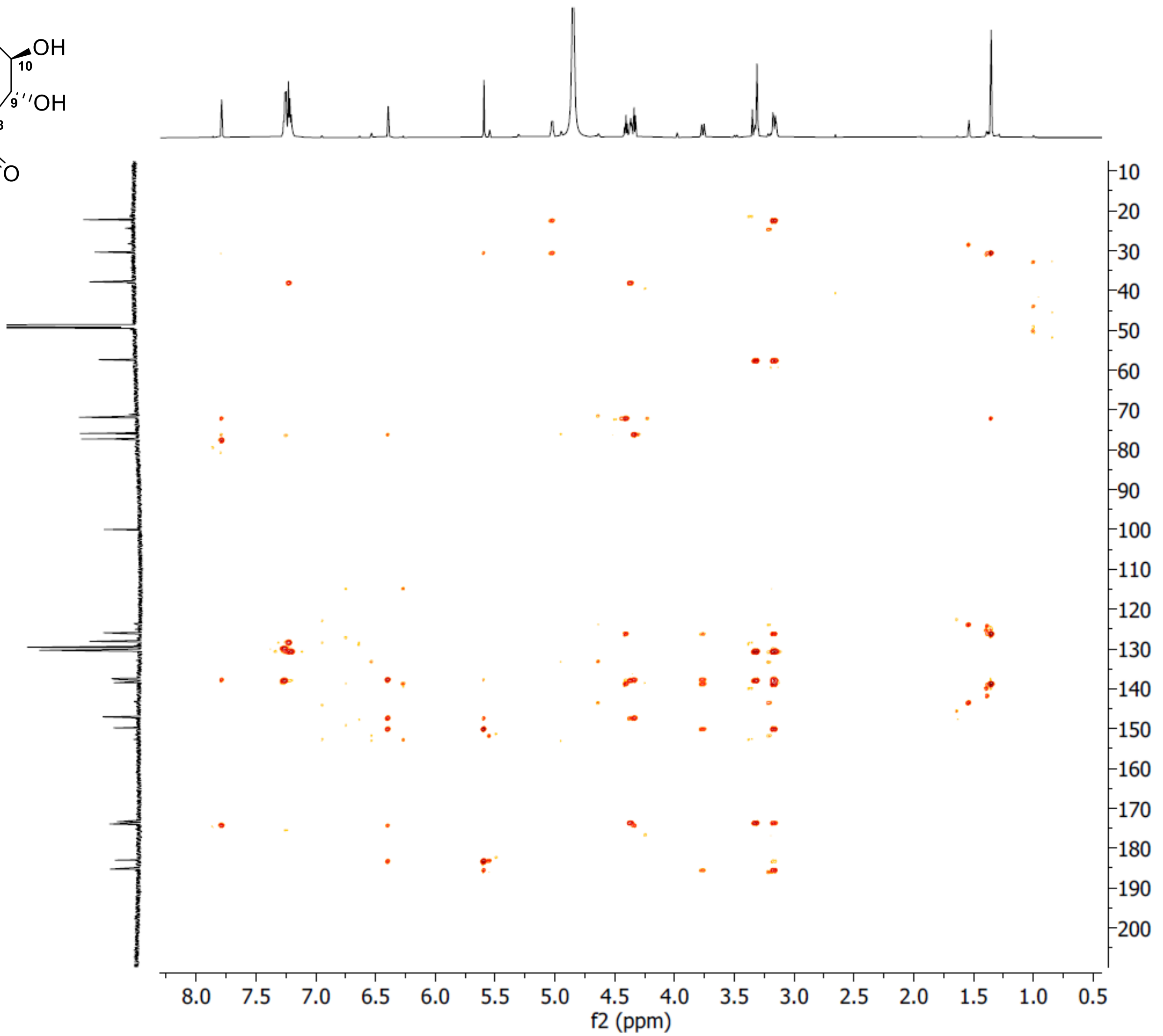

	Parameter	Value
1	Title	Sep24-2024_700_NB.88.ser
2	Comment	Broel PDR2 6a<br><br>NUS sampling 25%
3	Origin	Bruker BioSpin GmbH
4	Owner	nmrsu
5	Instrument	Avance Neo
6	Solvent	MeOD
7	Temperature	303.0
8	Pulse Sequence	hmbcetgpl3nd
9	Experiment	HMBC
10	Probe	Z168794_0004 (CPP1.1 TCI 700S3 H&F-C/N-D-05 Z XT)
11	Number of Scans	4
12	Receiver Gain	101.0
13	Relaxation Delay	2.0000
14	Pulse Width	7.9800
15	Acquisition Time	0.3482
16	Acquisition Date	2024-09-24T18:06:03
17	Modification Date	2024-09-24T18:06:04
18	Spectrometer Frequency	(700.28, 176.10)
19	Spectral Width	(5882.4, 38742.7)
20	Lowest Frequency	(124.2, -1485.3)
21	Nucleus	(1H, 13C)
22	Acquired Size	(2048, 256)
23	Spectral Size	(2048, 2048)
24	Digital Resolution	(2.87, 18.92)

Figure S16: ^{1}H-^{13}C HMBC NMR (CD_3OD, 700 MHz, 176 MHz) spectrum of **1** with 25% NUS sampling.

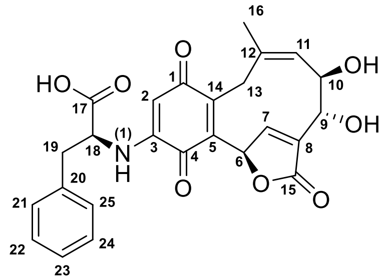

Dryinone A (**1**)

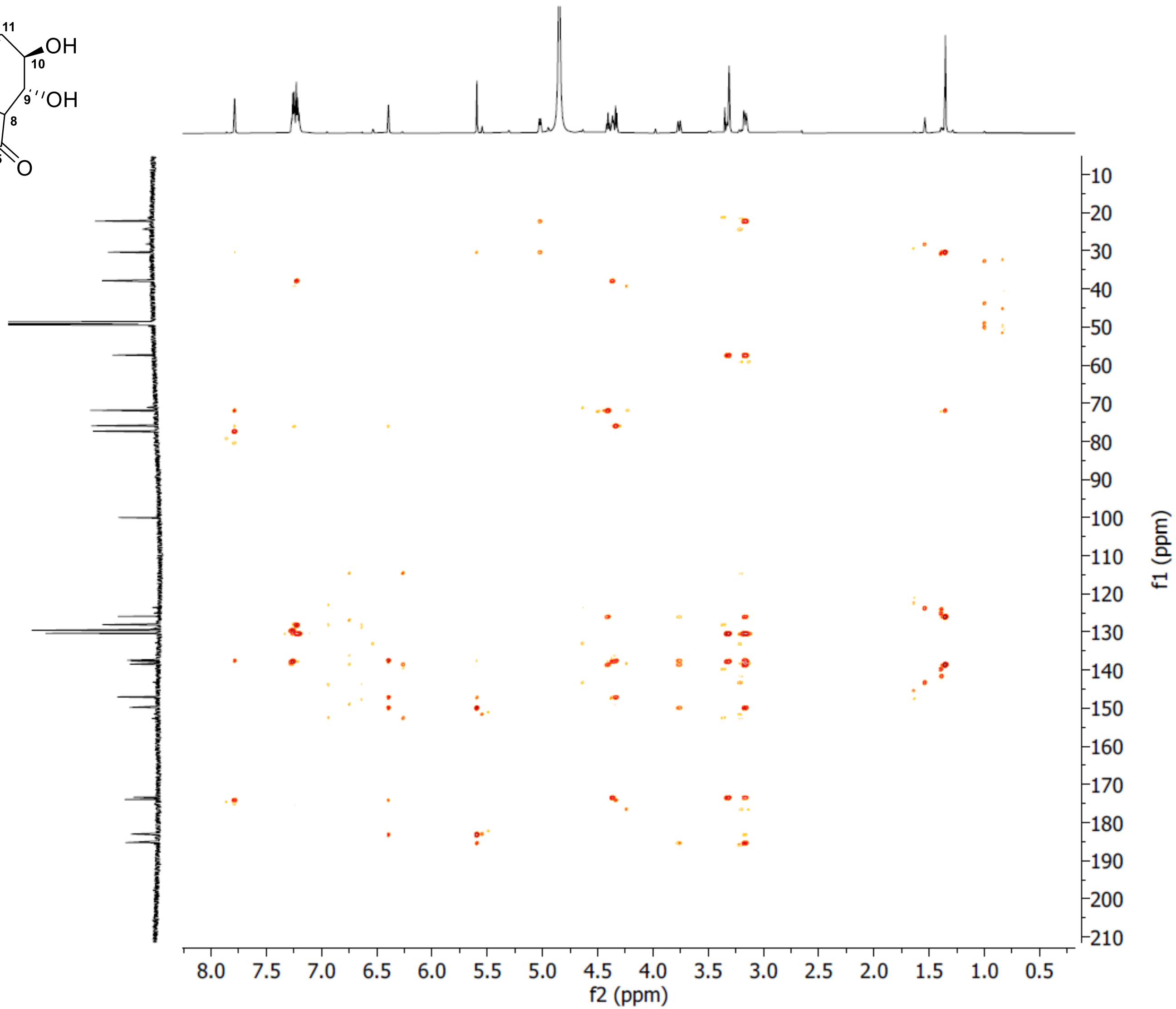

	Parameter	Value
1	Title	Sep24-2024_700_NB.91.ser
2	Comment	Broel PDR2 6a<br><br>NUS sampling 50%
3	Origin	Bruker BioSpin GmbH
4	Owner	nmrsu
5	Instrument	Avance Neo
6	Solvent	MeOD
7	Temperature	303.0
8	Pulse Sequence	hmbcetgpl3nd
9	Experiment	HMBC
10	Probe	Z168794_0004 (CPP1.1 TCI 700S3 H&F-C/N-D-05 Z XT)
11	Number of Scans	4
12	Receiver Gain	101.0
13	Relaxation Delay	2.0000
14	Pulse Width	7.9800
15	Acquisition Time	0.3482
16	Acquisition Date	2024-09-24T23:27:01
17	Modification Date	2024-09-24T23:27:02
18	Spectrometer Frequency	(700.28, 176.10)
19	Spectral Width	(5882.4, 38742.7)
20	Lowest Frequency	(122.1, -1523.0)
21	Nucleus	(1H, 13C)
22	Acquired Size	(2048, 512)
23	Spectral Size	(2048, 2048)
24	Digital Resolution	(2.87, 18.92)

Figure S17: ^{1}H-^{13}C HMBC NMR (CD_3OD, 700 MHz, 176 MHz) spectrum of **1** with 50% NUS sampling.

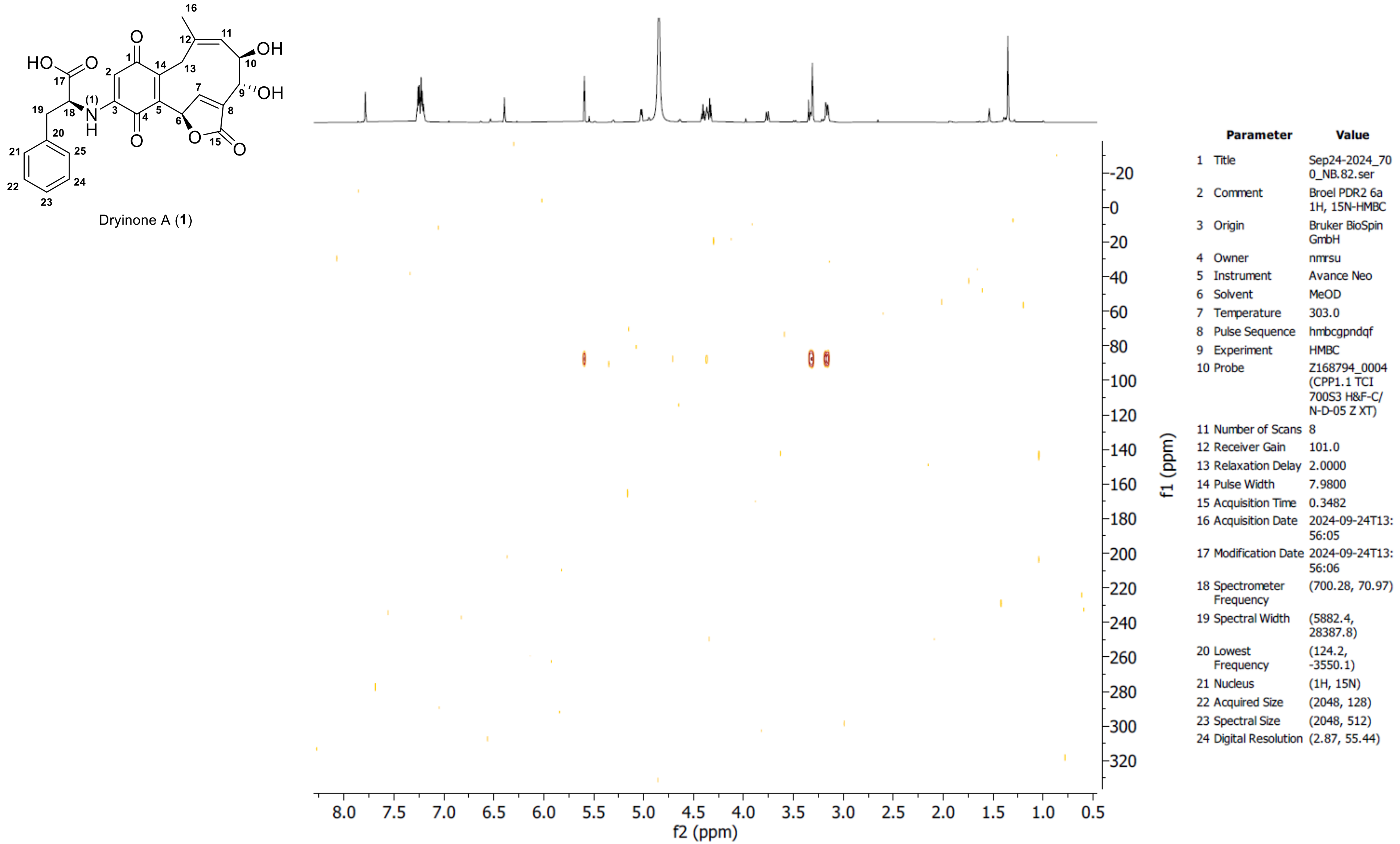

Figure S 18: ^{1}H-^{15}N HMBC NMR (CD_3OD, 700 MHz, 71 MHz) spectrum of **1**.

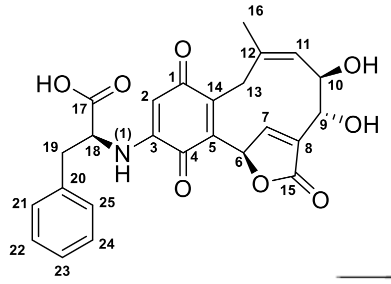

Dryinone A (**1**)

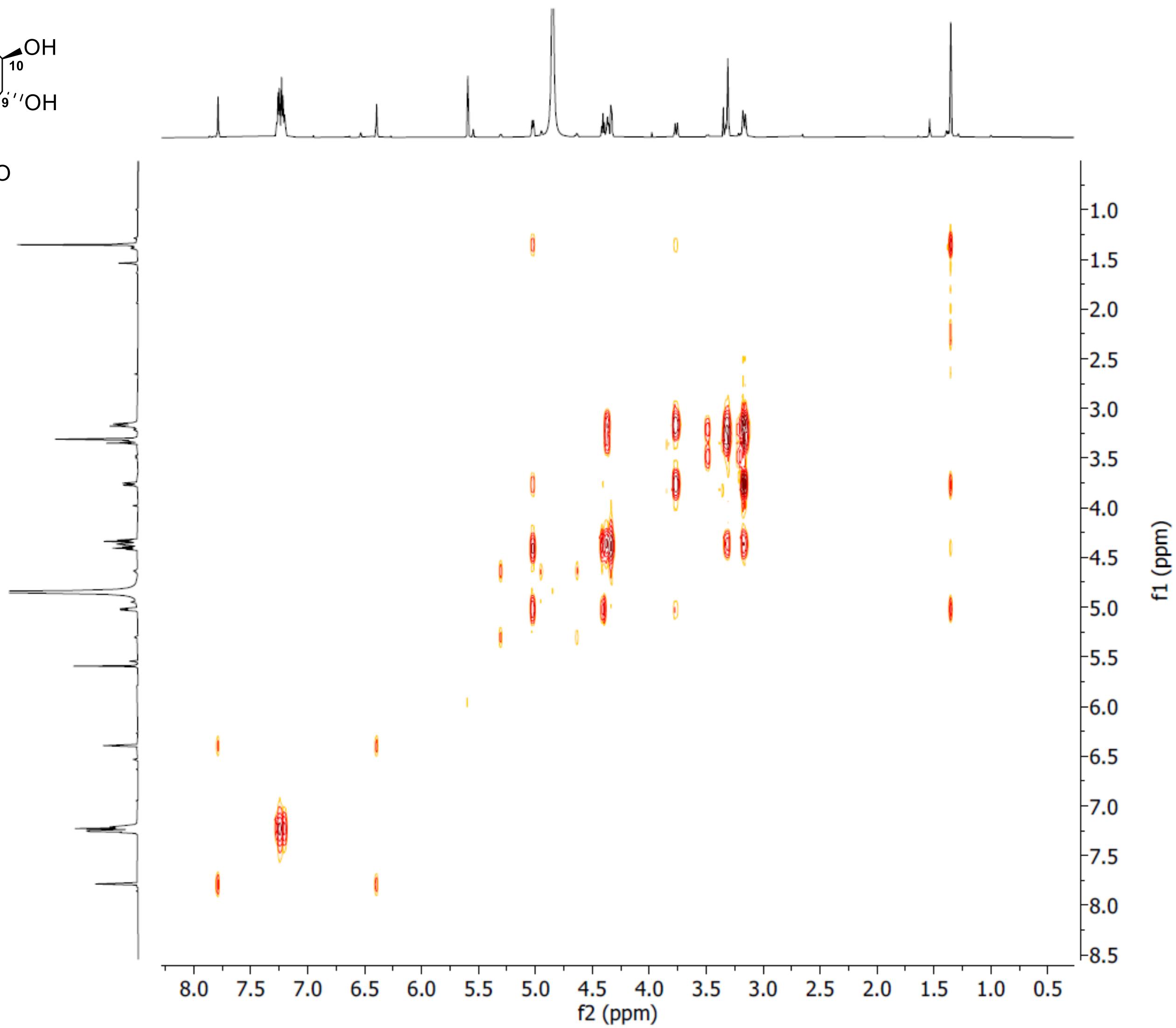

	Parameter	Value
1	Title	Sep24-2024_700_NB.81.ser
2	Comment	Broel PDR2 6a
3	Origin	Bruker BioSpin GmbH
4	Owner	nmrsu
5	Instrument	Avance Neo
6	Solvent	MeOD
7	Temperature	303.0
8	Pulse Sequence	cosygpmfqf
9	Experiment	COSY
10	Probe	Z168794_0004 (CPP1.1 TCI 700S3 H&F-C/N-D-05 Z XT)
11	Number of Scans	4
12	Receiver Gain	101.0
13	Relaxation Delay	1.9468
14	Pulse Width	7.9800
15	Acquisition Time	0.1638
16	Acquisition Date	2024-09-24T13:08:58
17	Modification Date	2024-09-24T13:08:59
18	Spectrometer Frequency	(700.28, 700.28)
19	Spectral Width	(6250.0, 9090.9)
20	Lowest Frequency	(-76.9, -1497.3)
21	Nucleus	(1H, 1H)
22	Acquired Size	(1024, 128)
23	Spectral Size	(1024, 1024)
24	Digital Resolution	(6.10, 8.88)

Figure S19: ^{1}H-^{1}H COSY NMR (CD_3OD, 700 MHz) spectrum of **1**.

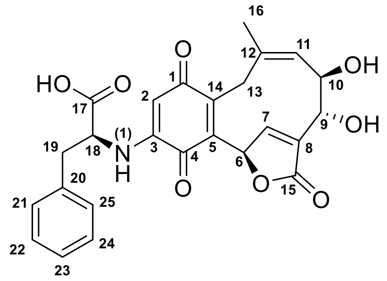

Dryinone A (**1**)

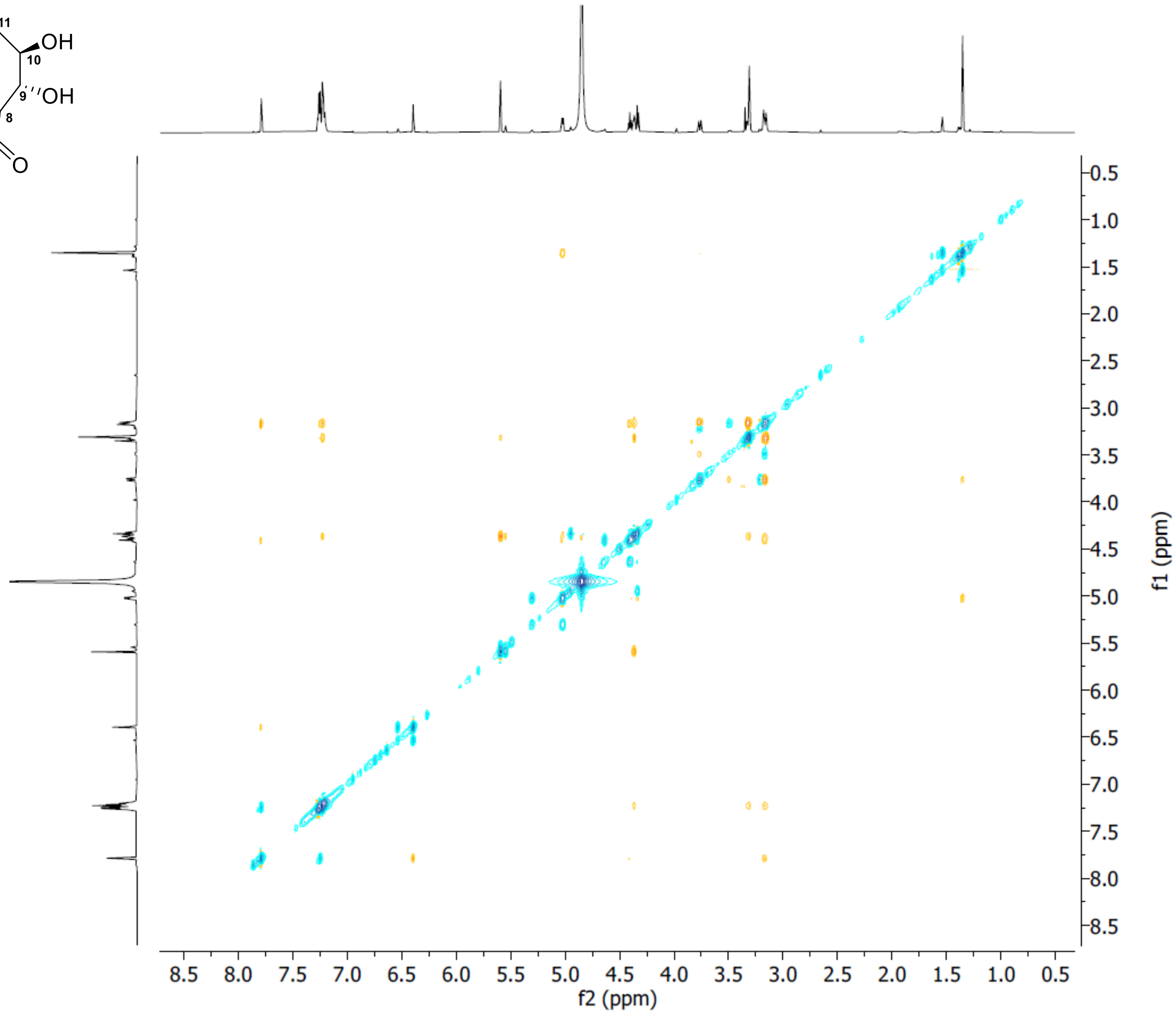

	Parameter	Value
1	Title	Sep24-2024_700_NB.90.ser
2	Comment	Broel PDR2 6a
3	Origin	Bruker BioSpin GmbH
4	Owner	nmrsu
5	Instrument	Avance Neo
6	Solvent	MeOD
7	Temperature	303.0
8	Pulse Sequence	noesygpphpp
9	Experiment	NOESY
10	Probe	Z168794_0004 (CPP1.1 TCI 700S3 H&F-C/N-D-05 Z XT)
11	Number of Scans	16
12	Receiver Gain	101.0
13	Relaxation Delay	2.0000
14	Pulse Width	7.9800
15	Acquisition Time	0.1741
16	Acquisition Date	2024-09-24T22:03:14
17	Modification Date	2024-09-24T22:03:15
18	Spectrometer Frequency	(700.28, 700.28)
19	Spectral Width	(5882.4, 5882.4)
20	Lowest Frequency	(220.0, 220.0)
21	Nucleus	(1H, 1H)
22	Acquired Size	(1024, 256)
23	Spectral Size	(1024, 1024)
24	Digital Resolution	(5.74, 5.74)

Figure S20: ^{1}H-^{1}H NOESY NMR (CD_3OD, 700 MHz) spectrum of **1**.

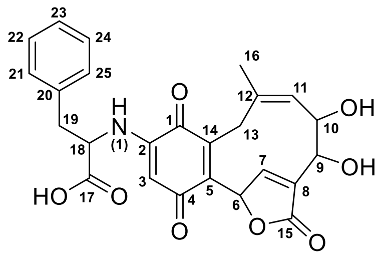

Dryinone B (**2**)

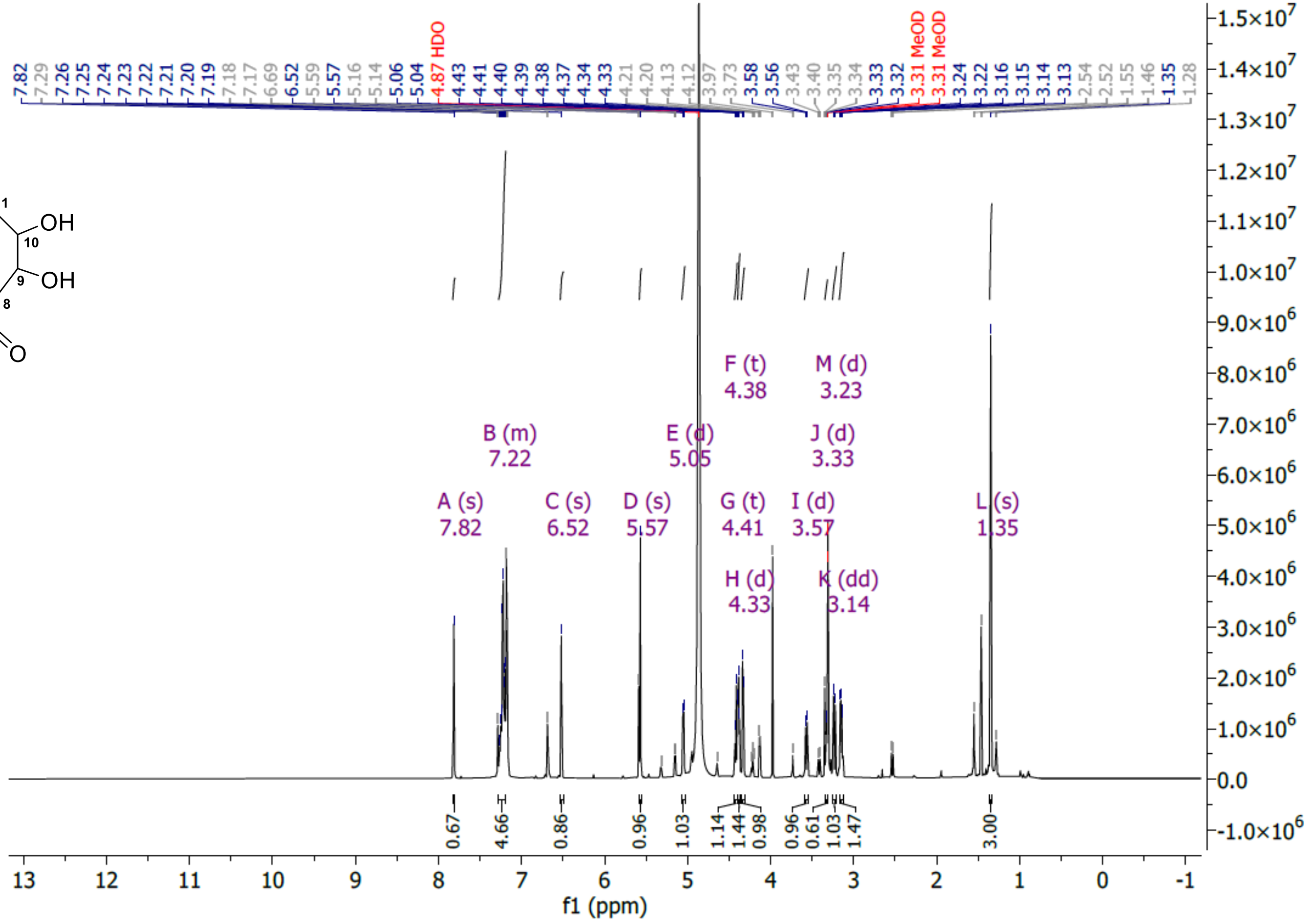

Parameter	Value
1 Title	Oct08-2024_700_NBr.90.fid
2 Comment	Broel PDR2_6b
3 Origin	Bruker BioSpin GmbH
4 Owner	nmrsu
5 Instrument	Avance Neo
6 Solvent	MeOD
7 Temperature	303.0
8 Pulse Sequence	zg30
9 Experiment	1D
10 Probe	Z168794_0004 (CPP1.1 TCI 700S3 H&F-C/ N-D-05 Z XT)
11 Number of Scans	64
12 Receiver Gain	52.0
13 Relaxation Delay	1.0000
14 Pulse Width	7.9800
15 Acquisition Time	2.3593
16 Acquisition Date	2024-10-08T12:50:36
17 Modification Date	2024-10-08T12:50:38
18 Spectrometer Frequency	700.28
19 Spectral Width	13888.9
20 Lowest Frequency	-2634.2
21 Nucleus	1H
22 Acquired Size	32768
23 Spectral Size	131072
24 Digital	0.11

^{1}H NMR (MeOD, 700 MHz) δ 7.82 (1H, s), 7.28 – 7.19 (5H, m), 6.52 (1H, s), 5.57 (1H, s), 5.05 (1H, d, *J* = 8.6 Hz), 4.41 (1H, t, *J* = 8.4 Hz), 4.38 (1H, t, *J* = 6.2 Hz), 4.33 (1H, d, *J* = 8.1 Hz), 3.57 (1H, d, *J* = 14.8 Hz), 3.33 (1H, d, *J* = 5.2 Hz), 3.23 (1H, d, *J* = 14.9 Hz), 3.14 (1H, dd, *J* = 14.0, 7.2 Hz), 1.35 (3H, s)

Figure S21: ^{1}H NMR (CD_3OD, 700 MHz) spectrum of **2**.

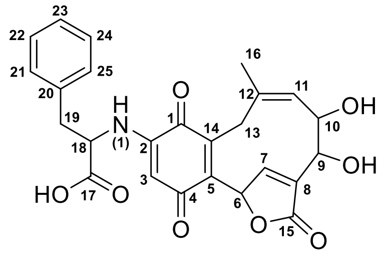

Dryinone B (**2**)

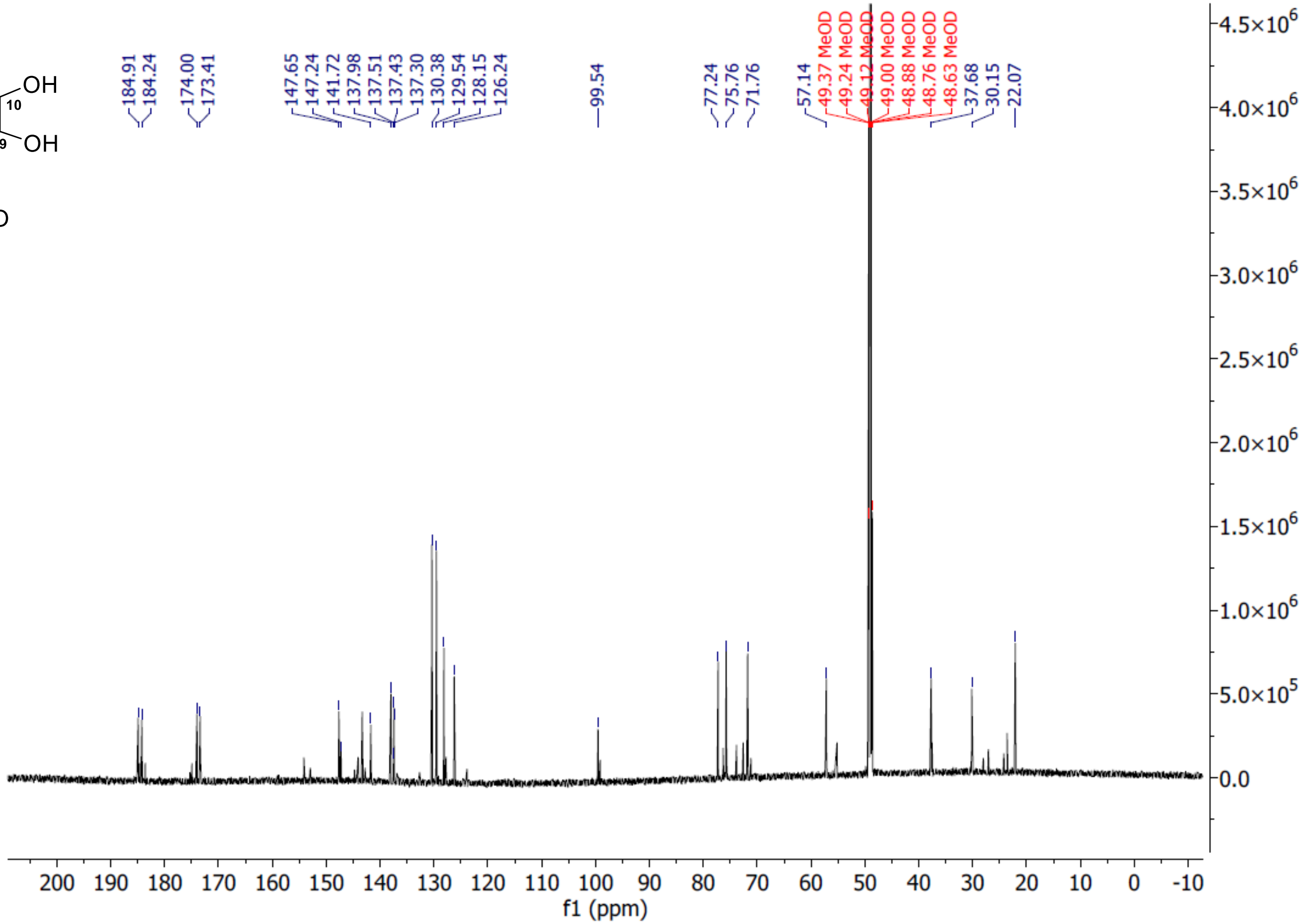

	Parameter	Value
1	Title	Oct08-2024_700_NBr.106.fid
2	Comment	Broel PDR2_6b
3	Origin	Bruker BioSpin GmbH
4	Owner	nmrsu
5	Instrument	Avance Neo
6	Solvent	MeOD
7	Temperature	293.0
8	Pulse Sequence	zgpg30
9	Experiment	1D
10	Probe	Z168794_0004 (CPP1.1 TCI 700S3 H&F-C/ N-D-05 Z XT)
11	Number of Scans	1024
12	Receiver Gain	101.0
13	Relaxation Delay	2.0000
14	Pulse Width	12.0000
15	Acquisition Time	0.7864
16	Acquisition Date	2024-10-08T16:25:31
17	Modification Date	2024-10-08T16:25:32
18	Spectrometer Frequency	176.10
19	Spectral Width	41666.7
20	Lowest Frequency	-2986.0
21	Nucleus	13C
22	Acquired Size	32768
23	Spectral Size	65536
24	Digital Resolution	0.64

^{13}C NMR (MeOD, 176 MHz) δ 184.9, 184.2, 174.0, 173.4, 147.6, 147.2, 141.7, 138.0, 137.5, 137.4, 137.3, 130.4, 129.5, 128.2, 126.2, 99.5, 77.2, 75.8, 71.8, 57.1, 37.7, 30.2, 22.1.

Figure S22: ^{13}C NMR (CD_3OD, 176 MHz) spectrum of **2**.

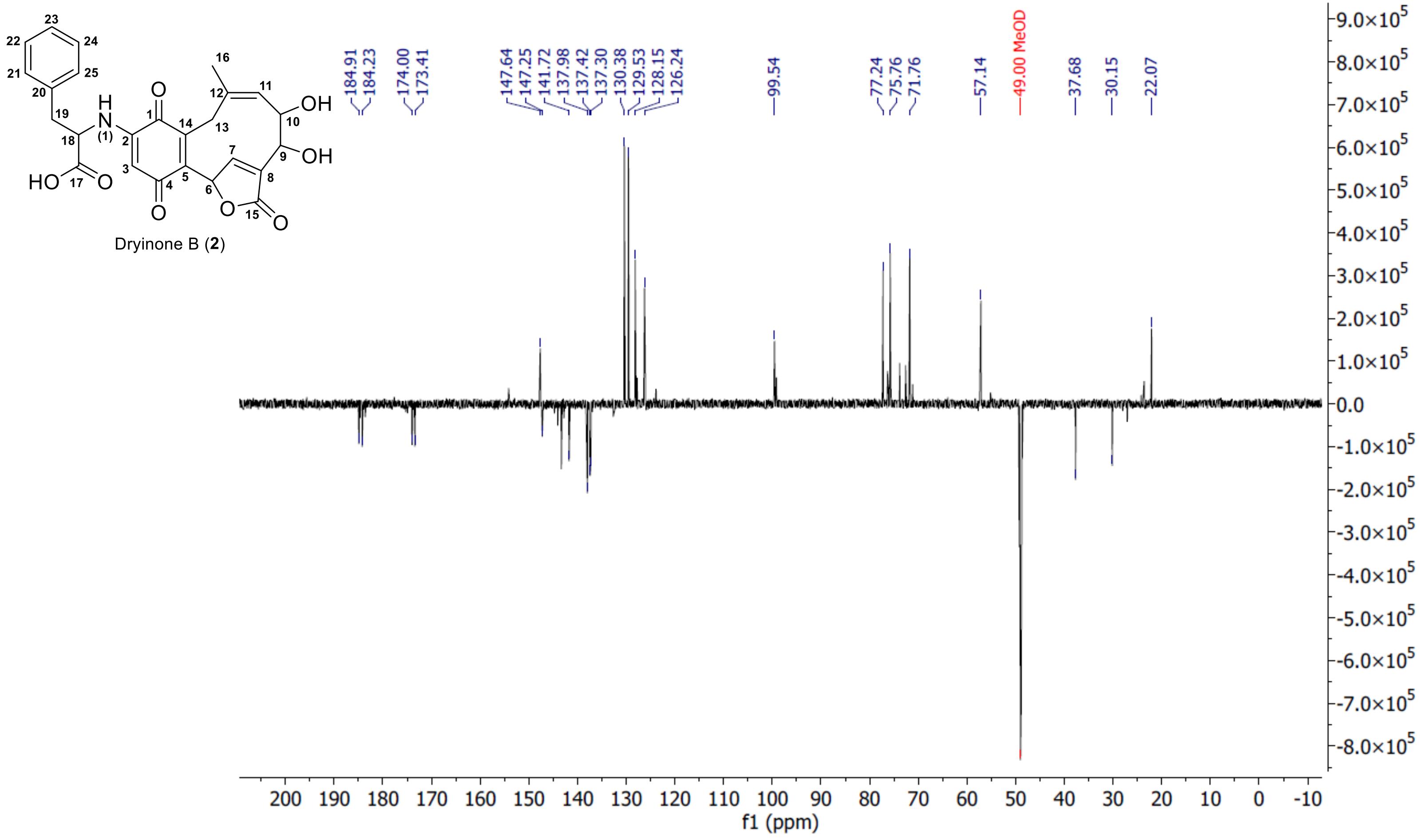

Parameter		Value
1	Title	Oct08-2024_700_NBr.107.fid
2	Comment	Broel PDR2_6b
3	Origin	Bruker BioSpin GmbH
4	Owner	nmrsu
5	Instrument	Avance Neo
6	Solvent	MeOD
7	Temperature	293.0
8	Pulse Sequence	jmod
9	Experiment	JMOD
10	Probe	Z168794_0004 (CPP1.1 TCI 700S3 H&F-C/ N-D-05 Z XT)
11	Number of Scans	256
12	Receiver Gain	101.0
13	Relaxation Delay	2.0000
14	Pulse Width	12.0000
15	Acquisition Time	0.7864
16	Acquisition Date	2024-10-08T16:38:10
17	Modification Date	2024-10-08T16:38:12
18	Spectrometer Frequency	176.10
19	Spectral Width	41666.7
20	Lowest Frequency	-2986.3
21	Nucleus	13C
22	Acquired Size	32768
23	Spectral Size	65536
24	Digital Resolution	0.64

^{13}C NMR (MeOD, 176 MHz) δ 184.9, 184.2, 174.0, 173.4, 147.6, 147.2, 141.7, 138.0, 137.4, 137.3, 130.4, 129.5, 128.1, 126.2, 99.5, 77.2, 75.8, 71.8, 57.1, 37.7, 30.2, 22.1.

Figure S23: *J*-modulated ^{13}C NMR (CD_3OD, 176 MHz) spectrum of **2.**

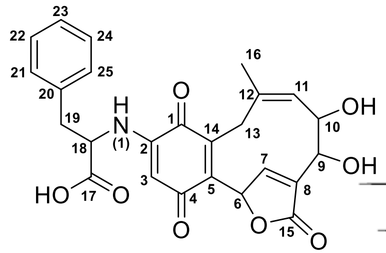

Dryinone B (**2**)

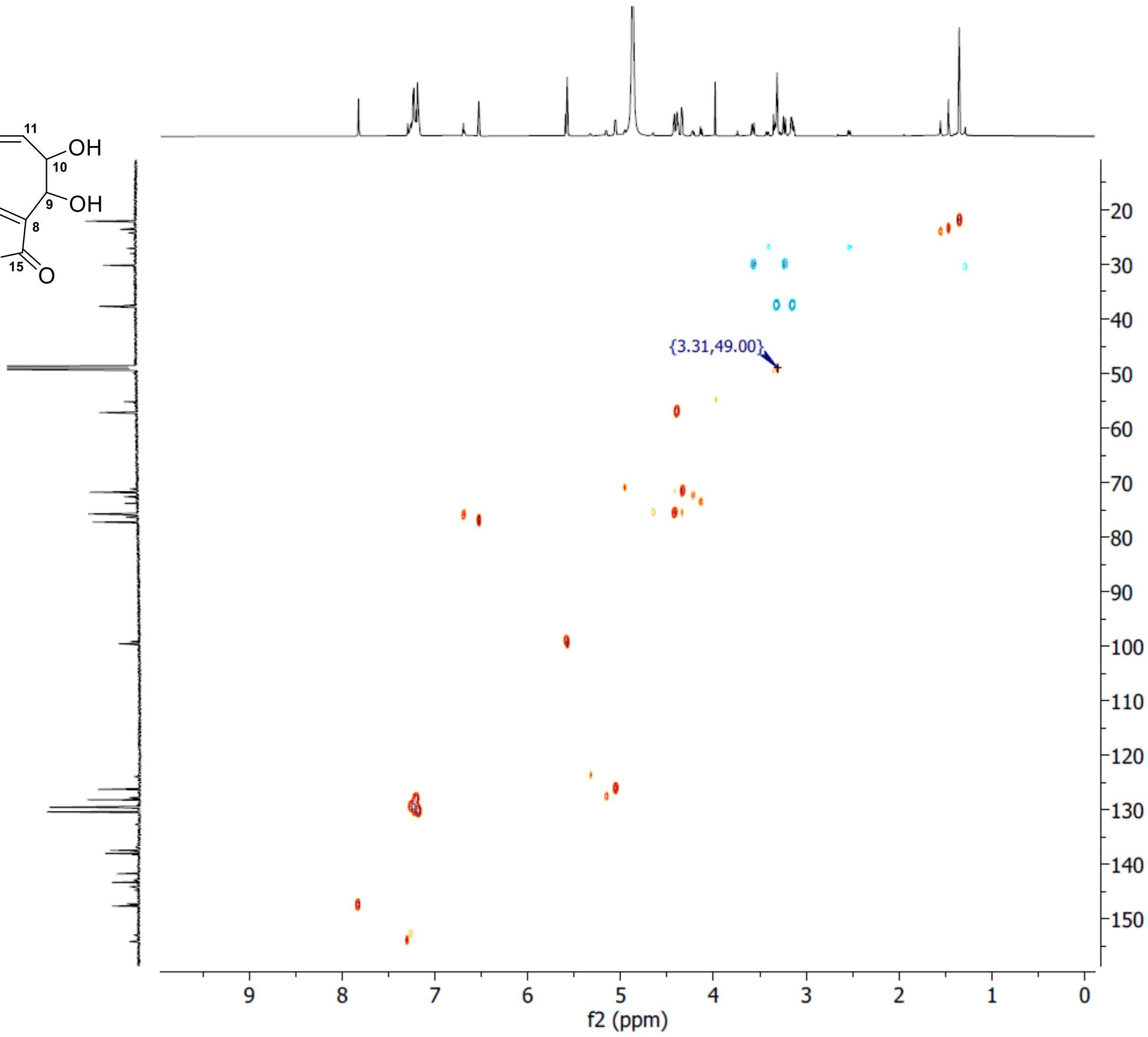

	Parameter	Value
1	Title	Oct08-2024_700_NBr.104.ser
2	Comment	Broel PDR2_6b
3	Origin	Bruker BioSpin GmbH
4	Owner	nmrsu
5	Instrument	Avance Neo
6	Solvent	MeOD
7	Temperature	293.0
8	Pulse Sequence	hsqcedetgpsisp2.3
9	Experiment	HSQC-EDITED
10	Probe	Z168794_0004 (CPP1.1 TCI 700S3 H&F-C/N-D-05 Z XT)
11	Number of Scans	4
12	Receiver Gain	101.0
13	Relaxation Delay	2.0000
14	Pulse Width	7.9800
15	Acquisition Time	0.0901
16	Acquisition Date	2024-10-08T14:53:48
17	Modification Date	2024-10-08T14:53:50
18	Spectrometer Frequency	(700.28, 176.10)
19	Spectral Width	(11363.6, 29056.3)
20	Lowest Frequency	(-2400.2, -1132.9)
21	Nucleus	(1H, 13C)
22	Acquired Size	(1024, 256)
23	Spectral Size	(1024, 1024)
24	Digital Resolution	(11.10, 28.38)

Figure S 24: ^{1}H-^{13}C HSQC NMR (CD_3OD, 700 MHz, 176 MHz) spectrum of **2**.

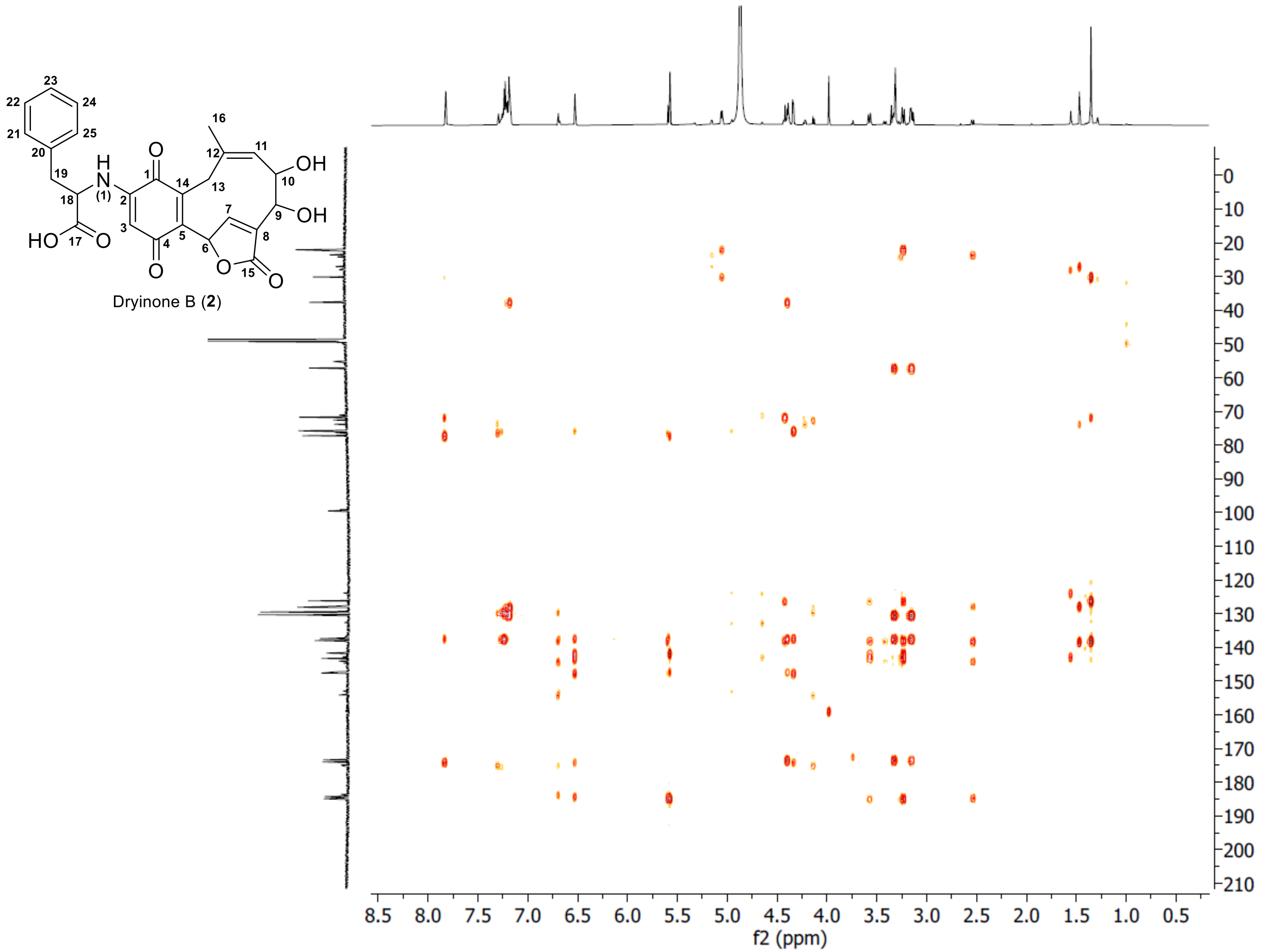

	Parameter	Value
1	Title	Oct08-2024_700_NBr.105.ser
2	Comment	Broel PDR2_6b
3	Origin	Bruker BioSpin GmbH
4	Owner	nmrsu
5	Instrument	Avance Neo
6	Solvent	MeOD
7	Temperature	293.0
8	Pulse Sequence	hmbcetgpl3nd
9	Experiment	HMBC
10	Probe	Z168794_0004 (CPP1.1 TCI 700S3 H&F-C/N-D-05 Z XT)
11	Number of Scans	4
12	Receiver Gain	101.0
13	Relaxation Delay	2.0000
14	Pulse Width	7.9800
15	Acquisition Time	0.3482
16	Acquisition Date	2024-10-08T15:36:20
17	Modification Date	2024-10-08T15:36:22
18	Spectrometer Frequency	(700.28, 176.10)
19	Spectral Width	(5882.4, 38742.7)
20	Lowest Frequency	(116.6, -1501.3)
21	Nucleus	(1H, 13C)
22	Acquired Size	(2048, 256)
23	Spectral Size	(2048, 1024)
24	Digital Resolution	(2.87, 37.83)

Figure S25: ^{1}H-^{13}C HMBC NMR (CD_3OD, 700 MHz, 176 MHz) spectrum of **2.**

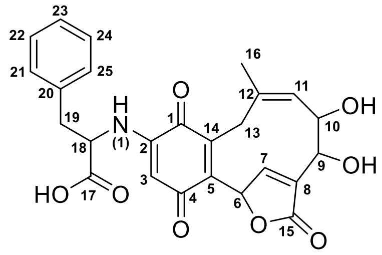

Dryinone B (**2**)

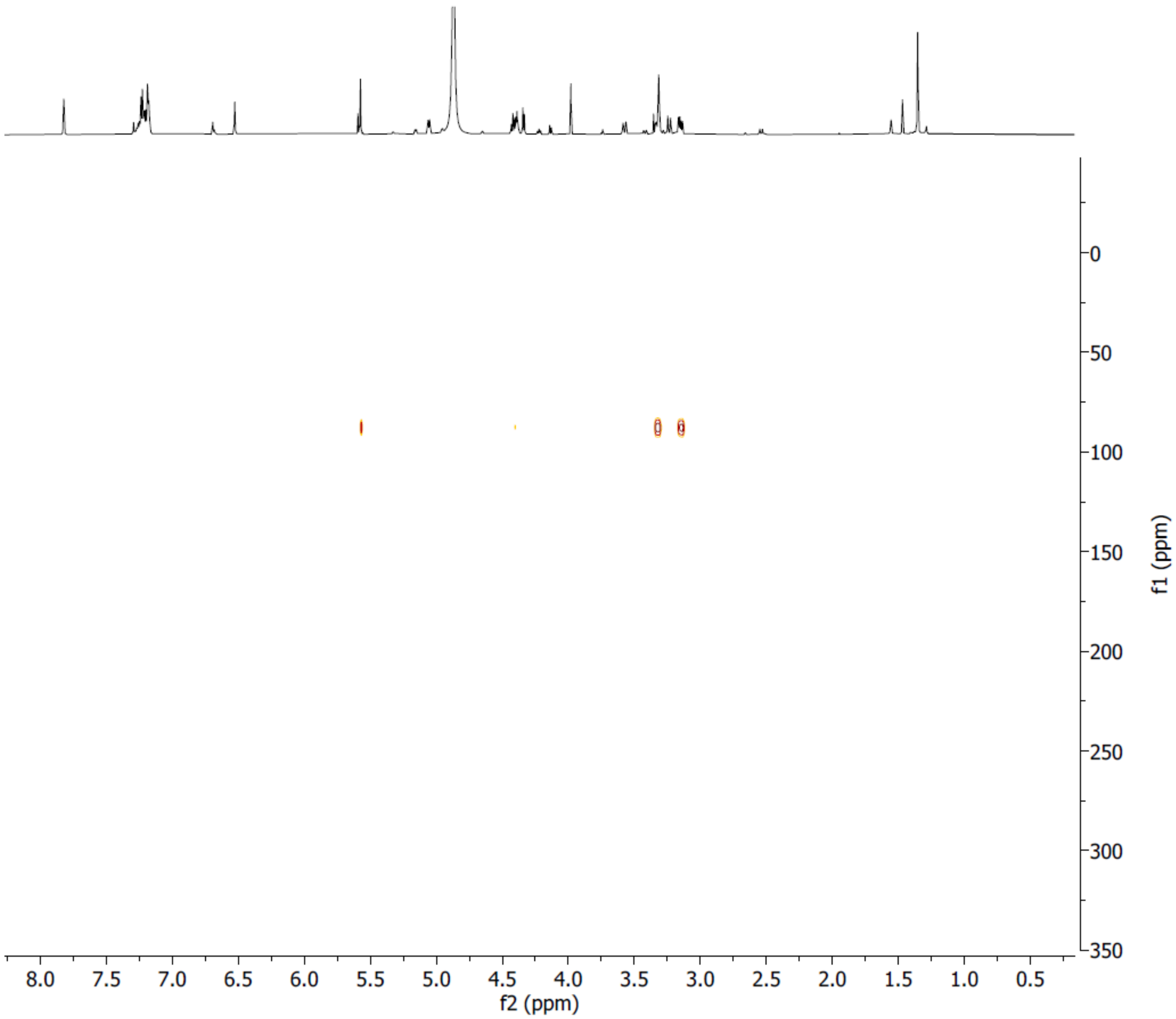

	Parameter	Value
1	Title	Oct08-2024_700_NBr.103.ser
2	Comment	Broel PDR2_6b
3	Origin	Bruker BioSpin GmbH
4	Owner	nmrsu
5	Instrument	Avance Neo
6	Solvent	MeOD
7	Temperature	293.0
8	Pulse Sequence	hmbcgpndqf
9	Experiment	HMBC
10	Probe	Z168794_0004 (CPP1.1 TCI 700S3 H&F-C/N-D-05 Z XT)
11	Number of Scans	8
12	Receiver Gain	101.0
13	Relaxation Delay	2.0000
14	Pulse Width	7.9800
15	Acquisition Time	0.3482
16	Acquisition Date	2024-10-08T14:15:50
17	Modification Date	2024-10-08T14:15:51
18	Spectrometer Frequency	(700.28, 70.97)
19	Spectral Width	(5882.4, 28387.8)
20	Lowest Frequency	(113.2, -3550.1)
21	Nucleus	(1H, 15N)
22	Acquired Size	(2048, 128)
23	Spectral Size	(2048, 512)
24	Digital Resolution	(2.87, 55.44)

Figure S26: ^{1}H-^{15}N HMBC NMR (CD_3OD, 700 MHz, 71 MHz) spectrum of **2**.

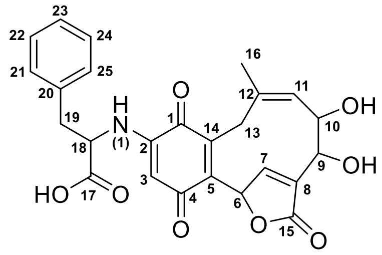

Dryinone B (**2**)

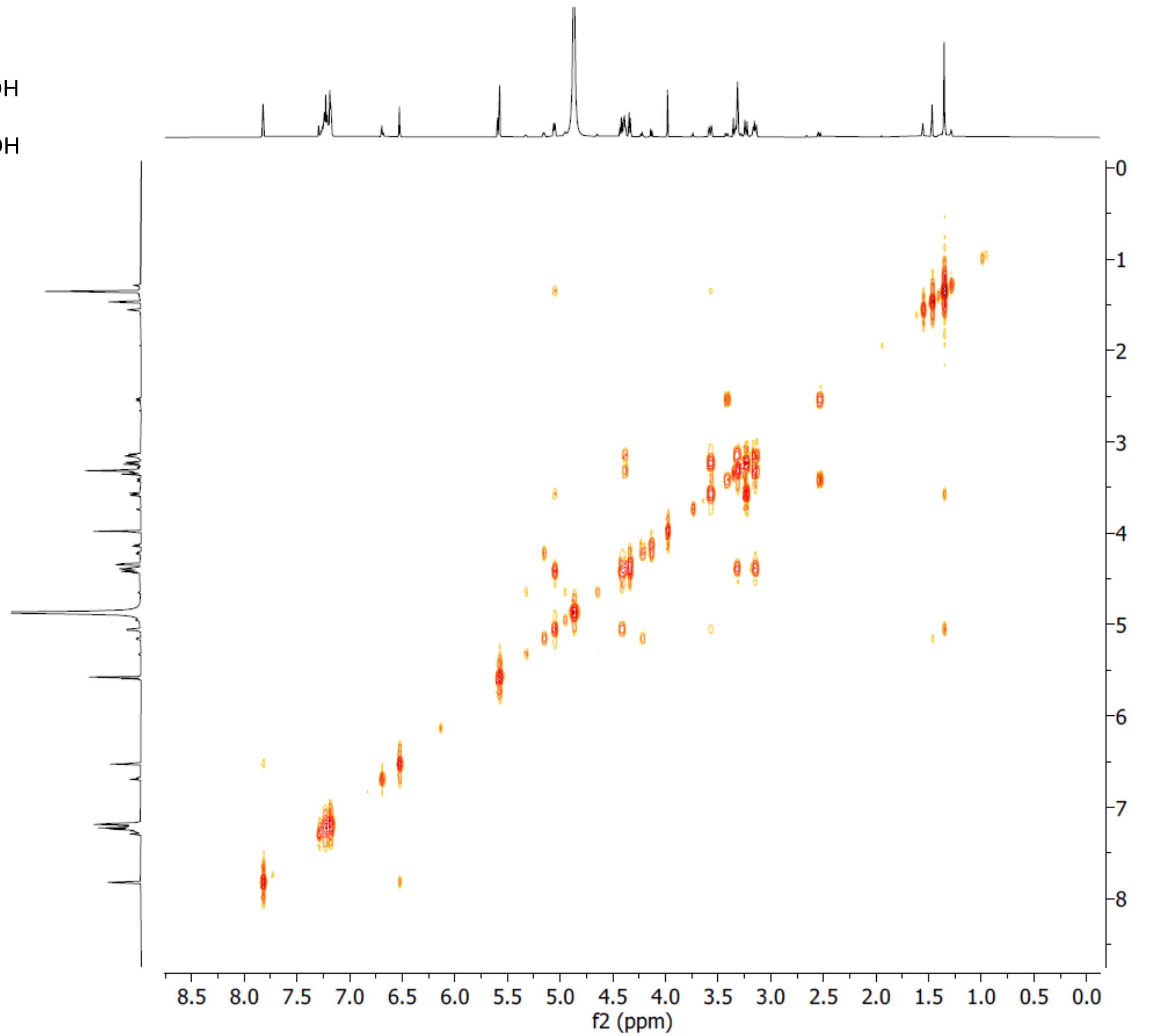

	Parameter	Value
1	Title	Oct08-2024_700_NBr.91.ser
2	Comment	Broel<br><br>PDR2_6b
3	Origin	Bruker BioSpin GmbH
4	Owner	nmrsu
5	Instrument	Avance Neo
6	Solvent	MeOD
7	Temperature	303.0
8	Pulse Sequence	cosygpppqf
9	Experiment	COSY
10	Probe	Z168794_0004 (CPP1.1 TCI 700S3 H&F-C/N-D-05 Z XT)
11	Number of Scans	1
12	Receiver Gain	101.0
13	Relaxation Delay	1.9468
14	Pulse Width	7.9800
15	Acquisition Time	0.1638
16	Acquisition Date	2024-10-08T13:06:12
17	Modification Date	2024-10-08T13:06:14
18	Spectrometer Frequency	(700.28, 700.28)
19	Spectral Width	(6250.0, 9090.9)
20	Lowest Frequency	(-94.0, -1514.4)
21	Nucleus	(1H, 1H)
22	Acquired Size	(1024, 256)
23	Spectral Size	(1024, 1024)
24	Digital Resolution	(6.10, 8.88)

Figure S27: ^{1}H-^{1}H COSY NMR (CD_3OD, 700 MHz) spectrum of **2**.

(6*R*,9*R*,10*R*,18*S*)-**1**

6	-5.632203000	0.456868000	-1.058065000
6	-4.261783000	-0.027376000	-0.621078000
8	-6.293151000	-0.499642000	-1.717441000
1	-7.187934000	-0.179541000	-1.933084000
8	-6.090859000	1.554841000	-0.810028000
6	-4.460388000	-1.036109000	0.554007000
1	-3.787231000	-0.537838000	-1.458735000
7	-3.489413000	1.135542000	-0.219673000
6	-3.153952000	-1.572012000	1.069026000
1	-5.006381000	-0.522186000	1.354279000
1	-5.086640000	-1.852483000	0.180043000
6	-2.480075000	-0.925094000	2.107666000
1	-0.737046000	-0.879877000	3.367134000
1	0.300912000	-2.871805000	2.302267000
6	-1.244178000	-1.391471000	2.553405000
6	-2.569140000	-2.701731000	0.487928000
1	-3.083783000	-3.214973000	-0.321945000
6	-0.666796000	-2.511435000	1.962682000
6	-1.335828000	-3.168234000	0.930275000
1	-2.927116000	-0.050780000	2.575873000
1	-0.891731000	-4.045460000	0.466716000
8	1.230905000	-1.506069000	-0.647138000
6	-2.154524000	1.306002000	-0.127728000
1	2.665427000	0.800127000	-2.630818000
6	2.449002000	-0.002715000	-1.935206000
8	-1.625802000	-0.481953000	-1.605394000
6	-0.237033000	2.581451000	0.700168000
6	-1.632275000	2.348033000	0.596019000
6	2.560466000	-1.723397000	-0.452697000
6	0.741121000	1.673531000	-0.017176000
8	2.954758000	-2.598826000	0.294038000
8	0.211732000	3.519156000	1.389033000
6	0.252064000	0.674381000	-0.790190000
6	1.062816000	-0.365040000	-1.542827000
6	2.196536000	1.997817000	0.207621000
6	-1.207984000	0.423740000	-0.893905000
1	-2.290114000	3.017506000	1.145705000
6	2.901015000	1.104871000	1.216775000
1	2.716950000	2.012573000	-0.749818000
1	2.230788000	3.018460000	0.608222000
6	4.121871000	0.583284000	1.028623000
1	1.847655000	1.803996000	2.959347000
6	2.141874000	0.858298000	2.486285000
1	1.212854000	0.309209000	2.277961000
1	2.730497000	0.274474000	3.199148000
6	4.972331000	0.666024000	-0.207053000
1	4.543118000	-0.028799000	1.828893000
6	4.788890000	-0.586678000	-1.107294000
6	3.320653000	-0.775666000	-1.279207000
1	-4.034645000	1.814002000	0.304651000
1	0.475091000	-0.716847000	-2.398514000
1	5.218399000	-1.460447000	-0.591168000
8	5.406762000	-0.390590000	-2.377791000
1	6.333215000	-0.163079000	-2.210745000
1	4.708303000	1.530662000	-0.826561000
8	6.361697000	0.844101000	0.098750000
1	6.633642000	0.134497000	0.699142000

E_{el} = -1624.31381002 Eh

E_{ZPVE} = 0.45058971 Eh

(6*R*,9*R*,10*R*,18*S*)-**1**

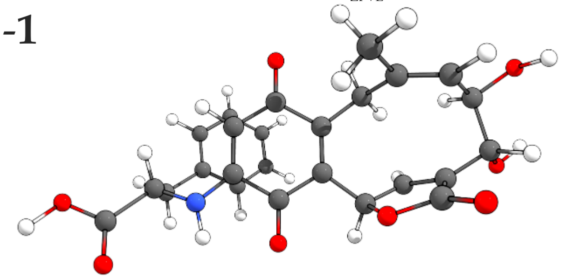

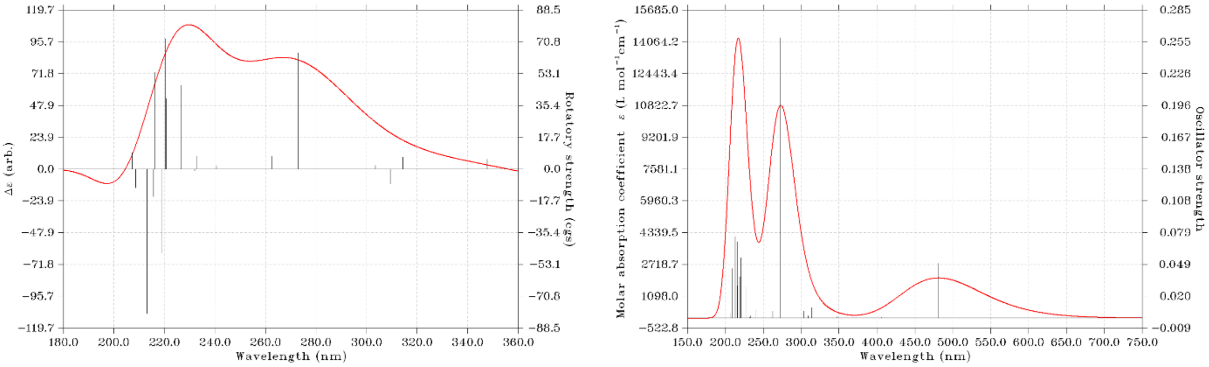

Figure S28: Detailed information on the most stable conformer of the (6*R*,9*R*,10*R*,18*S*)-**1** isomer, computed via DFT at the CAM-B3LYP/def2-TZVP/CPCM(MeOH) level of theory using n_{roots} = 20, as well as the visualized UV/vis and ECD spectra.

(6*R*,9*R*,10*S*,18*S*)-**1**

6	-5.484964000	1.756946000	-0.233544000
6	-4.444122000	0.650571000	-0.241130000
8	-6.619835000	1.353057000	-0.810413000
1	-7.283690000	2.063641000	-0.745803000
8	-5.322620000	2.851172000	0.268220000
6	-4.864944000	-0.431473000	0.801746000
1	-4.429346000	0.200433000	-1.239683000
7	-3.154820000	1.223309000	0.068478000
6	-3.859504000	-1.547273000	0.860929000
1	-4.960600000	0.056605000	1.778666000
1	-5.848474000	-0.809769000	0.505187000
6	-2.822085000	-1.521787000	1.796484000
1	-1.027036000	-2.466224000	2.511946000
1	-1.090599000	-4.277222000	0.812321000
6	-1.831009000	-2.500367000	1.781128000
6	-3.899881000	-2.583280000	-0.076795000
1	-4.704833000	-2.612246000	-0.808761000
6	-1.868016000	-3.517667000	0.830081000
6	-2.910855000	-3.561579000	-0.094087000
1	-2.784992000	-0.722745000	2.533802000
1	-2.950903000	-4.358468000	-0.832300000
8	2.424468000	2.652705000	-0.326737000
6	-1.973375000	0.794313000	-0.390225000
1	2.411285000	0.220232000	1.992466000
6	2.679881000	0.961881000	1.249004000
8	-1.009611000	2.404148000	1.041143000
6	-0.435657000	-0.540804000	-1.712710000
6	-1.754085000	-0.168203000	-1.338226000
6	3.739568000	2.286779000	-0.262178000
6	0.762678000	0.088748000	-1.025158000
8	4.580597000	2.821148000	-0.958483000
8	-0.229527000	-1.390240000	-2.600693000
6	0.566087000	1.079204000	-0.121436000
6	1.662289000	1.836412000	0.606252000
6	2.105312000	-0.456649000	-1.446948000
6	-0.800181000	1.502996000	0.235325000
1	-2.567449000	-0.695518000	-1.824187000
6	2.639524000	-1.556853000	-0.538119000
1	1.959025000	-0.908599000	-2.436035000
1	2.822395000	0.354073000	-1.568922000
6	3.912059000	-1.643091000	-0.123640000
1	2.097796000	-3.395393000	0.450296000
6	1.644084000	-2.618490000	-0.171064000
1	0.800720000	-2.184735000	0.382483000
1	1.219890000	-3.086560000	-1.068267000
6	5.046584000	-0.680090000	-0.344951000
1	4.178804000	-2.510277000	0.483633000
6	5.120593000	0.382666000	0.802208000
6	3.884585000	1.207818000	0.722946000
1	-3.126613000	1.945330000	0.782863000
1	1.193373000	2.521847000	1.321551000
1	4.951160000	-0.138592000	-1.292772000
1	6.009982000	0.998417000	0.623854000
8	5.315003000	-0.243874000	2.066073000
8	6.295637000	-1.372913000	-0.429537000
1	4.568143000	-0.835320000	2.241720000
1	6.447278000	-1.809431000	0.422043000

E_{el} = -1624.32173279 Eh

E_{ZPVE} = 0.45077953 Eh

(6*R*,9*R*,10*S*,18*S*)-**1**

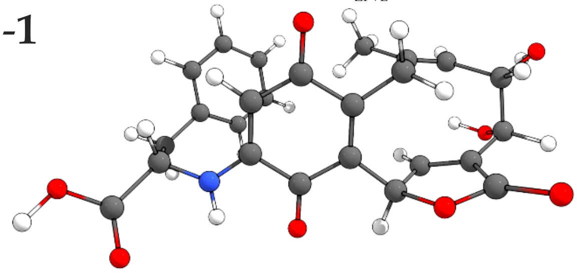

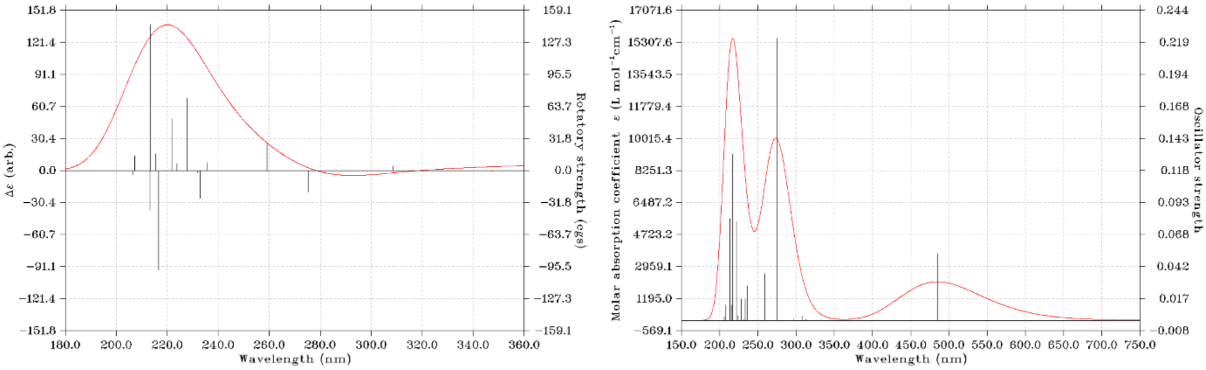

Figure S29: Detailed information on the most stable conformer of the (6*R*,9*R*,10*S*,18*S*)-**1** isomer, computed via DFT at the CAM-B3LYP/def2-TZVP/CPCM(MeOH) level of theory using n_{roots} = 20, as well as the visualized UV/vis and ECD spectra.

(6*R*,9*S*,10*R*,18*S*)-**1**

6	-5.384627000	1.961170000	-0.475045000
6	-4.406652000	0.804359000	-0.369127000
8	-6.515037000	1.580168000	-1.077015000
1	-7.142374000	2.325870000	-1.082576000
8	-5.184976000	3.075241000	-0.034567000
6	-4.925525000	-0.187132000	0.719959000
1	-4.377081000	0.290437000	-1.335348000
7	-3.099963000	1.320987000	-0.037095000
6	-3.968410000	-1.331332000	0.901585000
1	-5.047866000	0.369463000	1.656386000
1	-5.908130000	-0.546811000	0.398468000
6	-2.960167000	-1.267924000	1.867225000
1	-1.220450000	-2.210682000	2.710577000
1	-1.298700000	-4.147447000	1.155475000
6	-2.003384000	-2.275818000	1.959186000
6	-4.015866000	-2.434458000	0.044553000
1	-4.797684000	-2.493200000	-0.710309000
6	-2.048029000	-3.362850000	1.088949000
6	-3.062440000	-3.443736000	0.136282000
1	-2.916830000	-0.416275000	2.542871000
1	-3.108344000	-4.294097000	-0.539268000
8	2.520884000	2.386696000	-0.031162000
1	5.132732000	-0.576689000	1.988958000
6	-1.930630000	0.795427000	-0.423188000
1	2.388136000	-0.213689000	2.093810000
6	2.688034000	0.571081000	1.409822000
8	-0.927586000	2.324004000	1.071064000
6	-0.427521000	-0.657434000	-1.659855000
6	-1.733352000	-0.187404000	-1.355167000
1	6.238415000	1.385998000	0.286580000
6	3.824198000	1.993547000	0.056029000
6	0.774926000	-0.107999000	-0.917209000
8	4.703593000	2.588529000	-0.544029000
8	-0.237875000	-1.530219000	-2.528699000
6	0.600608000	0.870199000	0.003831000
6	1.708852000	1.517343000	0.811897000
6	2.107978000	-0.698726000	-1.299739000
6	-0.744266000	1.404937000	0.279041000
1	-2.555712000	-0.653008000	-1.887291000
6	2.572111000	-1.828304000	-0.388218000
1	1.987938000	-1.122631000	-2.304582000
1	2.859316000	0.084362000	-1.367684000
6	3.826086000	-1.990143000	0.063404000
1	1.092989000	-3.290618000	-0.950177000
6	1.513908000	-2.836716000	-0.043994000
1	1.908249000	-3.631995000	0.594411000
1	0.676585000	-2.357354000	0.480628000
6	5.035255000	-1.121808000	-0.101041000
1	4.006102000	-2.857147000	0.698475000
6	5.120807000	-0.040086000	1.033434000
6	3.916671000	0.833792000	0.948772000
1	-3.053785000	2.032670000	0.686686000
8	6.350369000	0.679447000	0.944065000
1	1.251361000	2.158094000	1.575412000
8	5.073724000	-0.505899000	-1.392657000
1	5.953672000	-0.120241000	-1.506579000
1	5.929843000	-1.749661000	0.031625000

E_{el} = -1624.32046283 Eh

E_{ZPVE} = 0.45094501 Eh

(6*R*,9*S*,10*R*,18*S*)-**1**

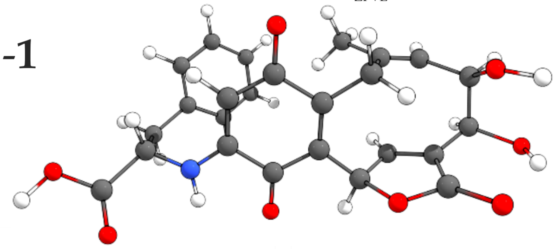

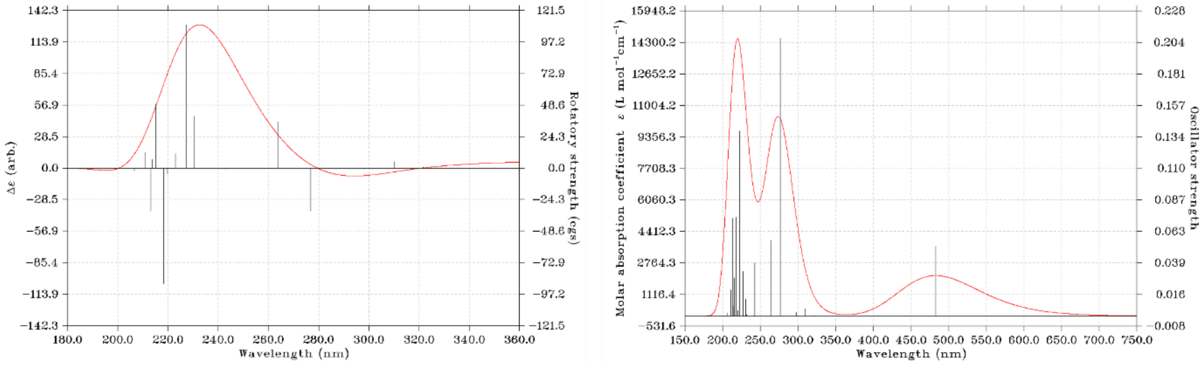

Figure S30: Detailed information on the most stable conformer of the (6*R*,9*S*,10*R*,18*S*)-**1** isomer, computed via DFT at the CAM-B3LYP/def2-TZVP/CPCM(MeOH) level of theory using n_{roots} = 20, as well as the visualized UV/vis and ECD spectra.

(6*R*,9*S*,10*S*,18*S*)-**1**

6	-5.492838000	1.841638000	-0.417136000
6	-4.472271000	0.718810000	-0.351073000
8	-6.632559000	1.423390000	-0.974986000
1	-7.283980000	2.148020000	-0.954217000
8	-5.314326000	2.961526000	0.017717000
6	-4.936158000	-0.308320000	0.729905000
1	-4.442172000	0.225190000	-1.328124000
7	-3.179722000	1.277815000	-0.032252000
6	-3.926328000	-1.407256000	0.906452000
1	-5.083782000	0.234042000	1.671236000
1	-5.900890000	-0.712739000	0.408093000
6	-2.919226000	-1.296909000	1.869255000
1	-1.145694000	-2.167570000	2.720380000
1	-1.156154000	-4.122273000	1.186229000
6	-1.925957000	-2.268026000	1.970107000
6	-3.931649000	-2.517702000	0.057765000
1	-4.711151000	-2.612458000	-0.695832000
6	-1.932259000	-3.364973000	1.111436000
6	-2.941864000	-3.490635000	0.158698000
1	-2.908361000	-0.439618000	2.539146000
1	-2.956423000	-4.348111000	-0.509189000
8	2.433638000	2.493441000	-0.093807000
1	5.074117000	-0.488116000	1.883726000
6	-1.999261000	0.798908000	-0.443382000
1	2.321705000	-0.097198000	2.046454000
6	2.617238000	0.693256000	1.366584000
8	-1.026409000	2.333890000	1.062967000
6	-0.466232000	-0.575290000	-1.730963000
6	-1.782768000	-0.160719000	-1.394876000
1	6.211240000	1.482667000	0.197583000
6	3.736886000	2.100707000	-0.017203000
6	0.729077000	-0.005610000	-0.989298000
8	4.607150000	2.661505000	-0.660481000
8	-0.260266000	-1.416217000	-2.626695000
6	0.535753000	0.952302000	-0.050685000
6	1.630053000	1.624816000	0.757185000
6	2.068635000	-0.567549000	-1.396371000
6	-0.823607000	1.436442000	0.251470000
1	-2.595811000	-0.644172000	-1.925336000
6	2.557948000	-1.710862000	-0.516080000
1	1.940606000	-0.975985000	-2.406596000
1	2.802271000	0.234920000	-1.464634000
6	3.819009000	-1.842070000	-0.082842000
1	0.695407000	-2.303486000	0.379405000
6	1.526533000	-2.748587000	-0.183939000
1	1.090533000	-3.177227000	-1.094997000
1	1.951688000	-3.557136000	0.416852000
6	4.969257000	-0.901357000	-0.246334000
1	4.058120000	-2.719553000	0.519666000
6	5.054409000	0.098350000	0.958205000
1	4.897388000	-0.306492000	-1.170793000
6	3.842730000	0.962574000	0.903045000
1	-3.147412000	1.981224000	0.700193000
8	6.164877000	-1.688240000	-0.275827000
1	6.908897000	-1.067881000	-0.251213000
8	6.281478000	0.826129000	0.910511000
1	1.158473000	2.267200000	1.510490000

E_{el} = -1624.32261539 Eh

E_{ZPVE} = 0.45048804 Eh

(6*R*,9*S*,10*S*,18*S*)-**1**

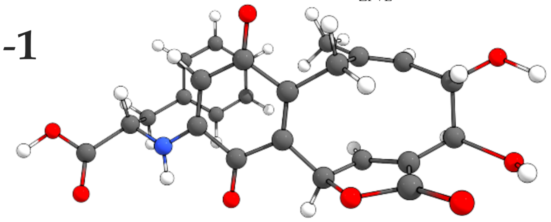

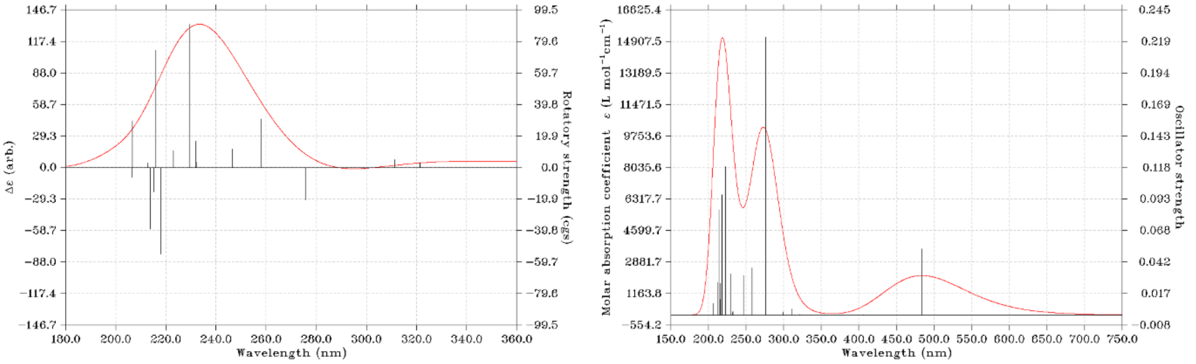

Figure S31: Detailed information on the most stable conformer of the (6*R*,9*S*,10*S*,18*S*)-**1** isomer, computed via DFT at the CAM-B3LYP/def2-TZVP/CPCM(MeOH) level of theory using n_{roots} = 20, as well as the visualized UV/vis and ECD spectra.

(6*S*,9*R*,10*R*,18*S*)-**1**

6	-5.554358000	-0.317577000	-1.544944000
6	-4.474833000	-0.312200000	-0.478138000
8	-6.663026000	-0.920247000	-1.104481000
1	-7.352181000	-0.863568000	-1.791256000
8	-5.444513000	0.194790000	-2.640700000
6	-4.893795000	0.684506000	0.652346000
1	-4.403731000	-1.320838000	-0.059079000
7	-3.216906000	0.072733000	-1.077153000
6	-3.803373000	0.815591000	1.678355000
1	-5.107127000	1.655109000	0.188790000
1	-5.816033000	0.304147000	1.102644000
6	-2.823439000	1.803314000	1.545896000
1	-0.968651000	2.603726000	2.286147000
1	-0.752290000	0.906600000	4.087665000
6	-1.729880000	1.837325000	2.408149000
6	-3.686762000	-0.118007000	2.712119000
1	-4.443919000	-0.891796000	2.823767000
6	-1.609189000	0.887168000	3.419314000
6	-2.597831000	-0.083279000	3.577321000
1	-2.905558000	2.537634000	0.747096000
1	-2.514872000	-0.820600000	4.371686000
8	2.061698000	2.090584000	-0.264012000
6	-2.009613000	-0.302802000	-0.627302000
1	2.677055000	-0.216367000	-2.631776000
1	5.410513000	-0.165900000	-2.125552000
6	2.740610000	0.527604000	-1.846512000
8	-1.150603000	1.515658000	-1.860558000
6	-0.410000000	-1.574160000	0.683849000
6	-1.732792000	-1.339755000	0.219865000
1	1.047196000	1.839650000	-2.041993000
6	3.413793000	1.930336000	-0.194187000
6	0.743714000	-0.737288000	0.163727000
8	4.084824000	2.548690000	0.613628000
8	-0.167644000	-2.459919000	1.525448000
6	0.493847000	0.272095000	-0.704827000
6	1.536000000	1.203497000	-1.294494000
6	2.104084000	-1.114464000	0.695824000
6	-0.887455000	0.568378000	-1.126335000
1	-2.509834000	-1.974402000	0.631237000
6	2.887080000	-2.061958000	-0.203741000
1	2.676187000	-0.217710000	0.930963000
1	1.929880000	-1.643058000	1.641328000
6	4.192152000	-1.928877000	-0.476968000
1	1.276330000	-2.891149000	-1.371475000
6	2.110546000	-3.228695000	-0.740974000
1	2.745981000	-3.887690000	-1.338431000
1	1.668506000	-3.812087000	0.076210000
6	5.125164000	-0.830865000	-0.076288000
1	4.657048000	-2.683759000	-1.112659000
6	5.175928000	0.298444000	-1.160974000
1	4.834754000	-0.362314000	0.877740000
6	3.832561000	0.940723000	-1.193873000
1	-3.227671000	0.910118000	-1.653363000
8	6.430707000	-1.404601000	0.050905000
1	7.048461000	-0.668049000	0.173916000
8	6.240253000	1.206229000	-0.875504000
1	5.974500000	1.747084000	-0.113138000

E_{el} = -1624.32217687 Eh

E_{ZPVE} = 0.45063374 Eh

(6*S*,9*R*,10*R*,18*S*)-**1**

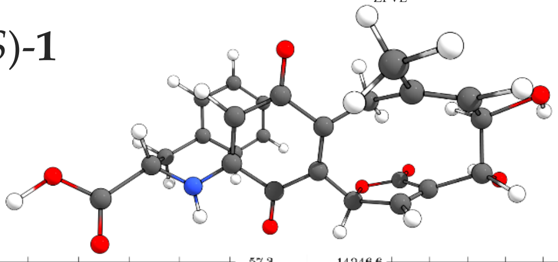

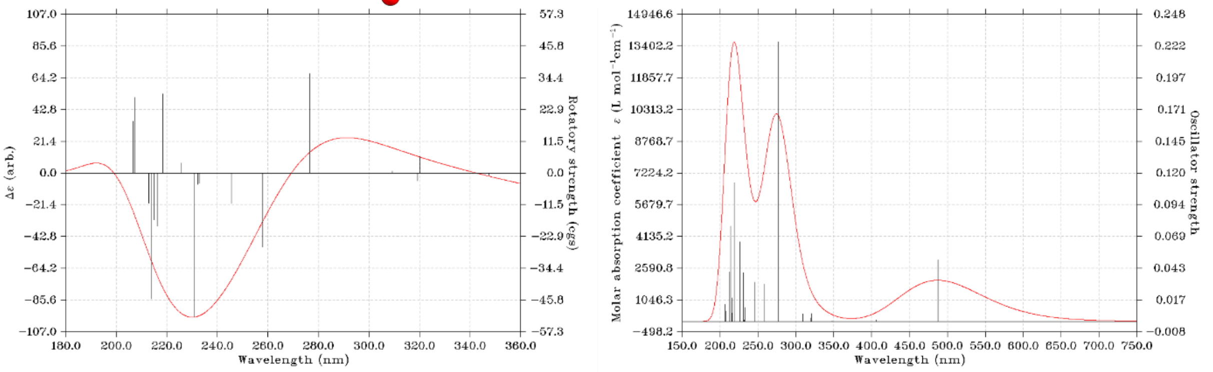

Figure S32: Detailed information on the most stable conformer of the (6*S*,9*R*,10*R*,18*S*)-**1** isomer, computed via DFT at the CAM-B3LYP/def2-TZVP/CPCM(MeOH) level of theory using n_{roots} = 20, as well as the visualized UV/vis and ECD spectra.

(6*S*,9*R*,10*S*,18*S*)-**1**

6	-5.506931000	0.157692000	-1.522693000
6	-4.424601000	-0.125259000	-0.497250000
8	-6.620732000	-0.528776000	-1.248666000
1	-7.313371000	-0.282312000	-1.887975000
8	-5.394750000	0.936724000	-2.447616000
6	-4.825907000	0.555508000	0.852902000
1	-4.367410000	-1.208144000	-0.349554000
7	-3.163148000	0.380689000	-0.987728000
6	-3.734720000	0.397777000	1.873805000
1	-5.020452000	1.616132000	0.654706000
1	-5.755495000	0.088698000	1.193477000
6	-2.730819000	1.363413000	1.990780000
1	-0.857067000	1.905346000	2.897605000
1	-0.683493000	-0.197174000	4.210281000
6	-1.637045000	1.151436000	2.826802000
6	-3.642499000	-0.768911000	2.637996000
1	-4.419634000	-1.526456000	2.556465000
6	-1.540626000	-0.026270000	3.564164000
6	-2.553873000	-0.980303000	3.478204000
1	-2.795155000	2.278053000	1.404787000
1	-2.490186000	-1.895510000	4.061206000
8	2.175536000	2.064547000	0.116410000
6	-1.958986000	-0.115854000	-0.667327000
1	2.708979000	0.289848000	-2.687646000
6	2.798951000	0.846881000	-1.762863000
8	-1.078579000	1.935972000	-1.434021000
6	-0.368739000	-1.689784000	0.275378000
6	-1.692598000	-1.330150000	-0.096684000
1	1.139699000	2.206799000	-1.658771000
6	3.528284000	1.888269000	0.116224000
6	0.793951000	-0.778591000	-0.064017000
8	4.225914000	2.362832000	0.996559000
8	-0.132203000	-2.750594000	0.884425000
6	0.555097000	0.409477000	-0.669567000
6	1.616138000	1.417585000	-1.064579000
6	2.158392000	-1.281172000	0.332128000
6	-0.824770000	0.830427000	-0.965963000
1	-2.478060000	-2.028615000	0.170090000
6	2.902016000	-2.003488000	-0.785349000
1	2.754453000	-0.467480000	0.738232000
1	2.003850000	-2.006267000	1.141152000
6	4.204685000	-1.848101000	-1.070900000
1	1.228911000	-2.500012000	-2.048820000
6	2.079384000	-2.994053000	-1.558885000
1	2.674602000	-3.493884000	-2.327497000
1	1.655490000	-3.756233000	-0.893162000
6	5.223952000	-0.924711000	-0.477618000
1	4.596390000	-2.443196000	-1.895364000
6	5.245351000	0.459274000	-1.218136000
6	3.909658000	1.100739000	-1.060852000
1	-3.163531000	1.330576000	-1.349584000
1	6.221252000	-1.358451000	-0.649556000
1	5.444324000	0.255106000	-2.276316000
8	5.018509000	-0.745636000	0.927524000
1	5.802299000	-0.305696000	1.285191000
8	6.322664000	1.264094000	-0.738876000
1	6.029991000	1.706238000	0.075024000

E_{el} = -1624.32000517 Eh

E_{ZPVE} = 0.45098523 Eh

(6*S*,9*R*,10*S*,18*S*)-**1**

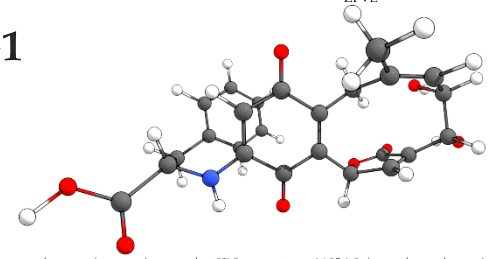

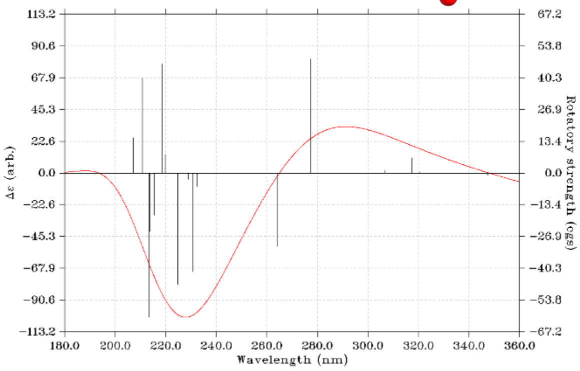

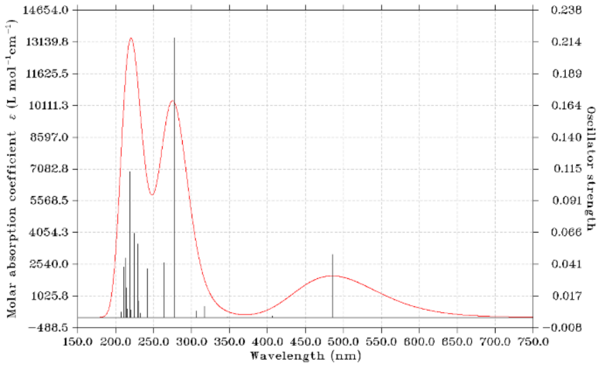

Figure S33: Detailed information on the most stable conformer of the (6*S*,9*R*,10*S*,18*S*)-**1** isomer, computed via DFT at the CAM-B3LYP/def2-TZVP/CPCM(MeOH) level of theory using n_{roots} = 20, as well as the visualized UV/vis and ECD spectra.

(6*S*,9*S*,10*R*,18*S*)-**1**

6	-5.500127000	-1.629227000	0.361233000
6	-4.436215000	-0.547212000	0.361721000
8	-6.587356000	-1.233030000	1.030207000
1	-7.272052000	-1.923803000	0.968690000
8	-5.398199000	-2.697181000	-0.208095000
6	-4.899409000	0.601775000	-0.593941000
1	-4.347560000	-0.153494000	1.378968000
7	-3.178739000	-1.115954000	-0.067866000
6	-3.831371000	1.650140000	-0.730022000
1	-5.132069000	0.159566000	-1.569903000
1	-5.818207000	1.026685000	-0.177335000
6	-2.876841000	1.557763000	-1.746675000
1	-1.061033000	2.353162000	-2.583081000
1	-0.834782000	4.125025000	-0.856311000
6	-1.802740000	2.443521000	-1.793473000
6	-3.710549000	2.667770000	0.220603000
1	-4.448958000	2.749476000	1.015965000
6	-1.676736000	3.438271000	-0.826932000
6	-2.640854000	3.556082000	0.173515000
1	-2.963360000	0.772072000	-2.494467000
1	-2.553727000	4.337582000	0.924001000
8	2.016307000	-0.171854000	-2.218081000
6	-1.970683000	-0.650589000	0.285885000
1	2.758624000	-2.525018000	0.064723000
6	2.784816000	-1.733834000	-0.675622000
8	-1.132442000	-1.844405000	-1.567899000
6	-0.368121000	0.683973000	1.528427000
6	-1.684392000	0.186416000	1.328478000
1	1.062461000	-1.979561000	-1.947237000
6	3.372524000	-0.058550000	-2.093659000
6	0.774271000	0.204892000	0.652066000
8	4.000480000	0.762175000	-2.733602000
8	-0.122817000	1.523170000	2.415687000
6	0.518588000	-0.664719000	-0.355327000
6	1.547703000	-1.220735000	-1.322855000
6	2.126712000	0.785699000	0.986399000
6	-0.860662000	-1.115896000	-0.618765000
1	-2.453034000	0.572770000	1.988467000
6	2.974573000	-0.070986000	1.917578000
1	2.662046000	1.036490000	0.071475000
1	1.933105000	1.727888000	1.514489000
6	4.285748000	-0.292143000	1.742666000
1	2.944083000	-1.182055000	3.762378000
6	2.264667000	-0.609532000	3.125424000
1	1.828839000	0.204758000	3.717354000
1	1.432509000	-1.264836000	2.833875000
6	5.167415000	0.132086000	0.600017000
1	4.799109000	-0.880530000	2.505420000
6	5.208643000	-0.959230000	-0.521111000
1	4.825197000	1.065848000	0.139904000
6	3.842884000	-1.038816000	-1.106924000
1	-3.200360000	-1.679800000	-0.913417000
8	6.498627000	0.399270000	1.051686000
1	6.856760000	-0.424291000	1.414770000
8	5.696769000	-2.197312000	-0.015021000
1	5.094635000	-2.517675000	0.672695000
1	5.933162000	-0.628867000	-1.274752000

E_{el} = -1624.32116573 Eh
E_{ZPVE} = 0.45086105 Eh

(6*S*,9*S*,10*R*,18*S*)-**1**

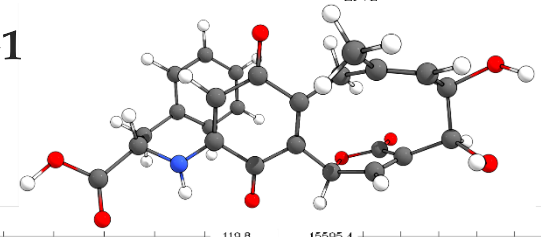

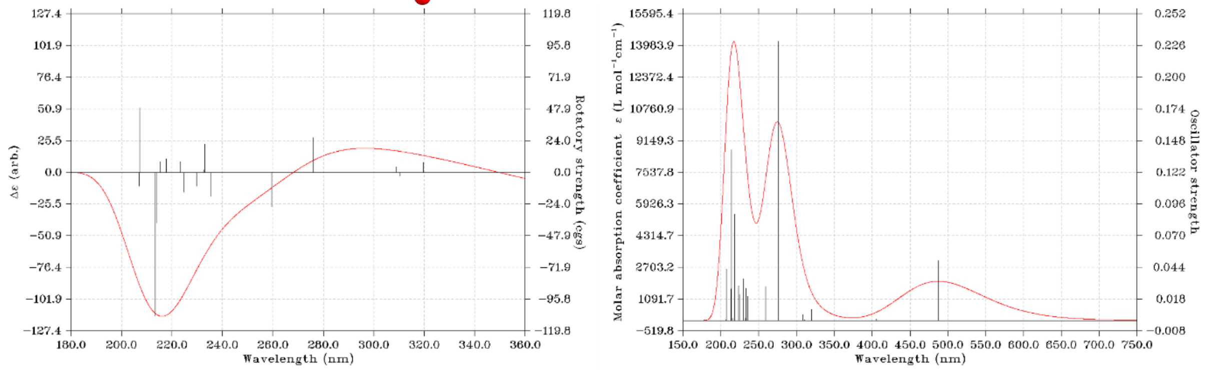

Figure S34: Detailed information on the most stable conformer of the (6*S*,9*S*,10*R*,18*S*)-**1** isomer, computed via DFT at the CAM-B3LYP/def2-TZVP/CPCM(MeOH) level of theory using n_{roots} = 20, as well as the visualized UV/vis and ECD spectra.

(6*S*,9*S*,10*S*,18*S*)-**1**

6	-5.449918000	1.597086000	-0.398372000
6	-4.390206000	0.511995000	-0.345349000
8	-6.559581000	1.156765000	-0.999286000
1	-7.238153000	1.855778000	-0.970289000
8	-5.325967000	2.706618000	0.079971000
6	-4.839883000	-0.571177000	0.690090000
1	-4.318652000	0.054157000	-1.337162000
7	-3.124822000	1.103022000	0.026508000
6	-3.774139000	-1.615134000	0.869476000
1	-5.050085000	-0.066081000	1.640368000
1	-5.768990000	-1.015598000	0.319635000
6	-2.800441000	-1.468266000	1.861267000
1	-0.972142000	-2.221977000	2.708558000
1	-0.784919000	-4.092897000	1.084576000
6	-1.728882000	-2.354967000	1.939564000
6	-3.674487000	-2.686657000	-0.022640000
1	-4.427172000	-2.809539000	-0.799083000
6	-1.624645000	-3.404939000	1.030357000
6	-2.607802000	-3.576331000	0.056641000
1	-2.870613000	-0.640774000	2.564428000
1	-2.537612000	-4.400627000	-0.648445000
8	2.133162000	0.383784000	2.125913000
1	6.025840000	0.806403000	1.056665000
6	-1.922018000	0.617677000	-0.316339000
1	2.798678000	2.525081000	-0.375696000
6	2.848772000	1.800019000	0.428232000
8	-1.056337000	1.942349000	1.434986000
6	-0.334666000	-0.791943000	-1.493605000
6	-1.651596000	-0.293786000	-1.299495000
1	1.161272000	2.153251000	1.715414000
6	3.493742000	0.294993000	2.003368000
6	0.819879000	-0.235113000	-0.684449000
8	4.144727000	-0.413046000	2.746746000
8	-0.098297000	-1.692125000	-2.321919000
6	0.578784000	0.701930000	0.263564000
6	1.632041000	1.340730000	1.149265000
6	2.176777000	-0.810708000	-0.999065000
6	-0.797411000	1.154277000	0.530678000
1	-2.431681000	-0.733316000	-1.911135000
6	2.988320000	0.006258000	-1.996509000
1	2.736292000	-0.982880000	-0.083139000
1	1.999921000	-1.790813000	-1.460147000
6	4.303155000	0.268985000	-1.897532000
1	1.381883000	1.102798000	-2.922542000
6	2.226727000	0.461356000	-3.208640000
1	2.865718000	1.021303000	-3.896329000
1	1.799771000	-0.395175000	-3.745159000
6	5.274895000	-0.038237000	-0.798162000
1	4.744112000	0.831788000	-2.719746000
6	5.284651000	1.089329000	0.298945000
1	6.288735000	-0.036330000	-1.226952000
6	3.927099000	1.165867000	0.903850000
1	-3.133607000	1.728074000	0.828039000
8	4.999395000	-1.306730000	-0.198244000
1	5.708556000	-1.496766000	0.432195000
8	5.733358000	2.320873000	-0.257852000
1	5.140632000	2.575174000	-0.979976000

E_{el} = -1624.31846625 Eh

E_{ZPVE} = 0.45086984 Eh

(6*S*,9*S*,10*S*,18*S*)-**1**

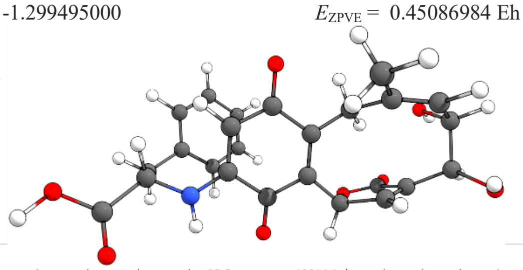

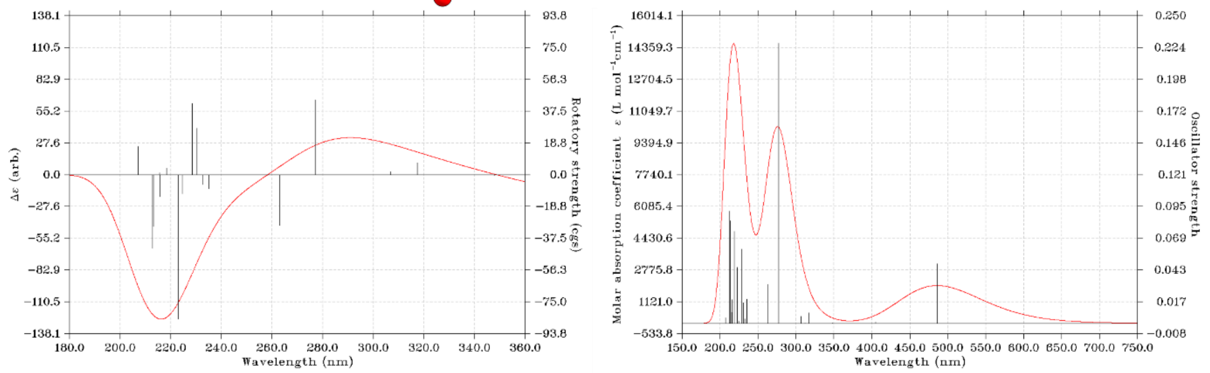

Figure S35: Detailed information on the most stable conformer of the (6*S*,9*S*,10*S*,18*S*)-**1** isomer, computed via DFT at the CAM-B3LYP/def2-TZVP/CPCM(MeOH) level of theory using n_{roots} = 20, as well as the visualized UV/vis and ECD spectra.

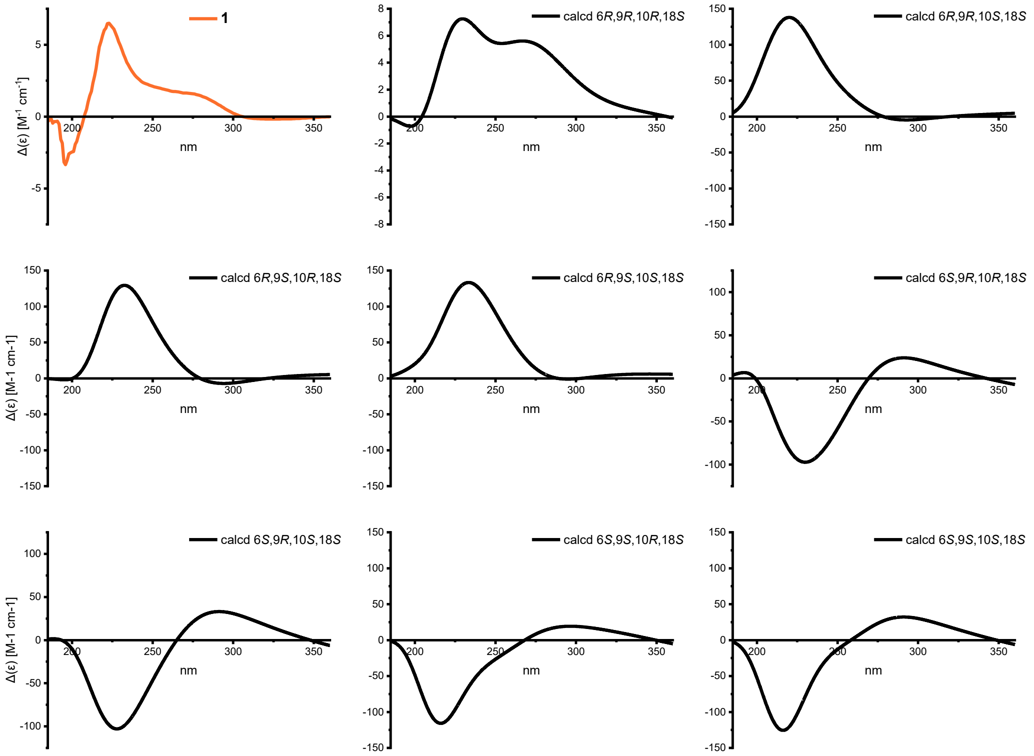

Figure S36: Experimental ECD spectrum of **1** and the time-dependent DFT spectra computed at the CAM-B3LYP/def2-TZVP/CPCM(MeOH) level of theory using n_{roots} = 20 for the eight different (18*S*)-isomers of **1**.

(6*R*,9*R*,10*R*,18*S*)-**2**

6	-5.360822000	-1.647639000	0.420115000
6	-4.347817000	-0.521872000	0.325484000
8	-6.506023000	-1.218268000	0.959937000
1	-7.153738000	-1.946365000	0.958599000
8	-5.175284000	-2.779561000	0.020960000
6	-4.796708000	0.460277000	-0.805755000
1	-4.341553000	0.012355000	1.280590000
7	-3.041491000	-1.075891000	0.050950000
6	-3.778720000	1.549037000	-0.995075000
1	-4.922128000	-0.118008000	-1.728636000
1	-5.768484000	0.874062000	-0.518675000
6	-3.791557000	2.681117000	-0.175307000
1	-2.786977000	4.493637000	0.396687000
1	-0.912679000	4.169432000	-1.200703000
6	-2.769171000	3.621556000	-0.251990000
6	-2.738142000	1.392201000	-1.914879000
1	-2.720307000	0.517496000	-2.561876000
6	-1.717694000	3.440918000	-1.149982000
6	-1.710598000	2.329729000	-1.989551000
1	-4.598144000	2.813651000	0.543274000
1	-0.900109000	2.188192000	-2.700083000
8	2.603405000	1.936136000	0.138664000
6	-1.692994000	0.446918000	1.372175000
1	3.129452000	-0.588391000	2.290959000
6	3.207621000	0.166720000	1.517552000
8	-0.231867000	1.915142000	2.437448000
6	-0.693472000	-1.040901000	-0.349530000
6	-1.878076000	-0.518454000	0.421527000
6	3.869245000	1.522825000	-0.181509000
6	0.664726000	-0.516202000	-0.056969000
8	4.515436000	2.093191000	-1.038560000
8	-0.877819000	-1.878226000	-1.224399000
6	0.790953000	0.467136000	0.861860000
6	2.104762000	1.122841000	1.236451000
6	1.790124000	-1.121483000	-0.868893000
6	-0.403753000	0.996019000	1.612136000
6	2.505949000	-2.273651000	-0.179356000
1	1.340058000	-1.525267000	-1.784900000
1	2.487547000	-0.348963000	-1.179742000
6	3.837937000	-2.456653000	-0.148919000
1	0.966975000	-2.856038000	1.212858000
6	1.599686000	-3.303139000	0.434021000
1	0.919667000	-3.721490000	-0.318935000
1	2.168698000	-4.121075000	0.883260000
6	4.947182000	-1.588435000	-0.664582000
1	4.190343000	-3.356512000	0.354209000
6	5.383202000	-0.507734000	0.392286000
6	4.220569000	0.379052000	0.668676000
1	-2.982682000	-1.742089000	-0.714399000
1	1.916493000	1.800784000	2.077870000
1	-2.515802000	0.873509000	1.935149000
1	6.209571000	0.067477000	-0.043236000
8	5.904972000	-1.139812000	1.556803000
1	5.220167000	-1.708969000	1.937443000
1	5.834708000	-2.222228000	-0.813484000
8	4.583672000	-0.959312000	-1.896410000
1	5.365458000	-0.504020000	-2.239947000

E_{el} = -1624.32104646 Eh
E_{ZPVE} = 0.45073412 Eh

(6*R*,9*R*,10*R*,18*S*)-**2**

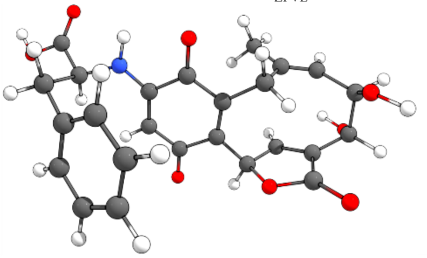

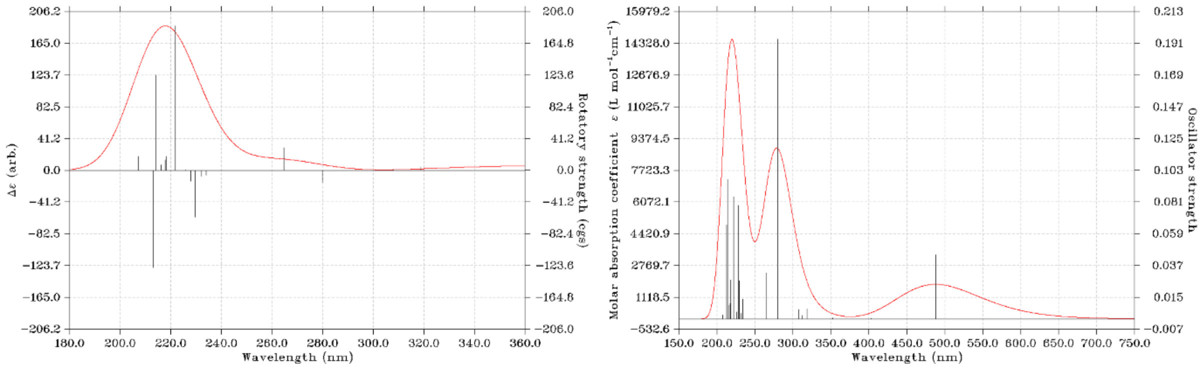

Figure S37: Detailed information on the most stable conformer of the (6*R*,9*R*,10*R*,18*S*)-**2** isomer, computed via DFT at the CAM-B3LYP/def2-TZVP/CPCM(MeOH) level of theory using n_{roots} = 20, as well as the visualized UV/vis and ECD spectra.

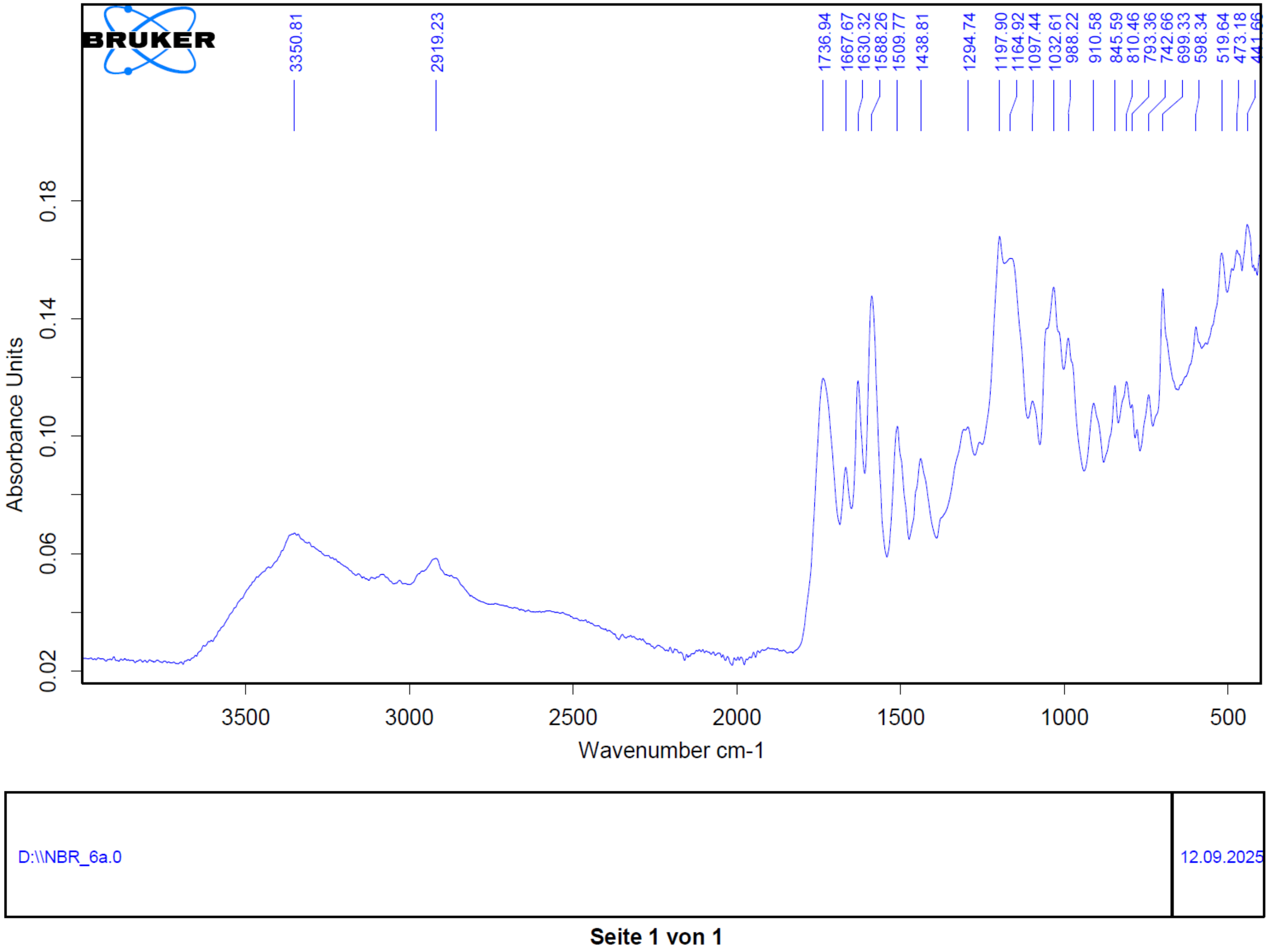

Figure S38: FTIR spectrum (ATR, neat) of **1**.

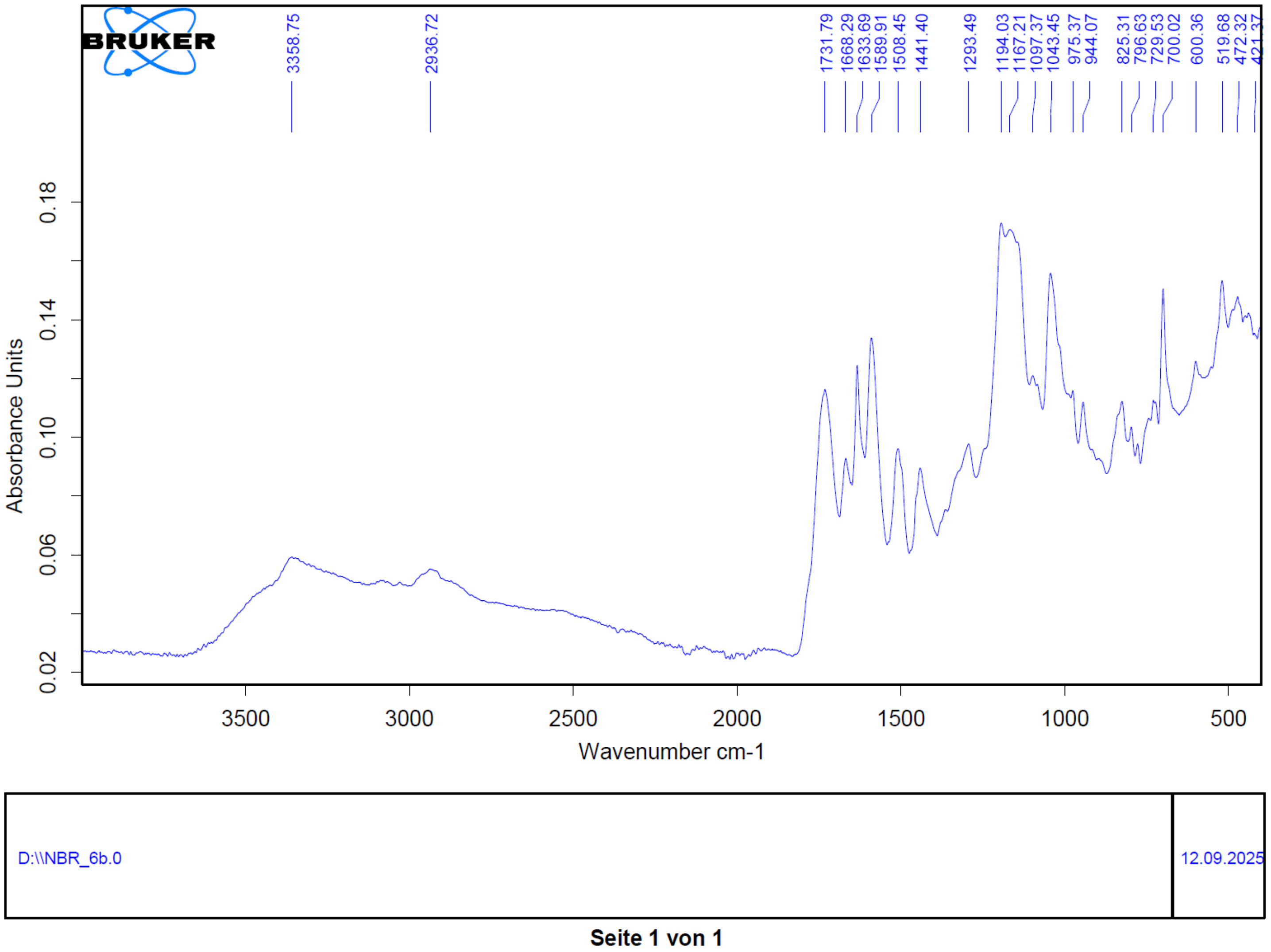

Figure S39: FTIR spectrum (ATR, neat) of **2**.

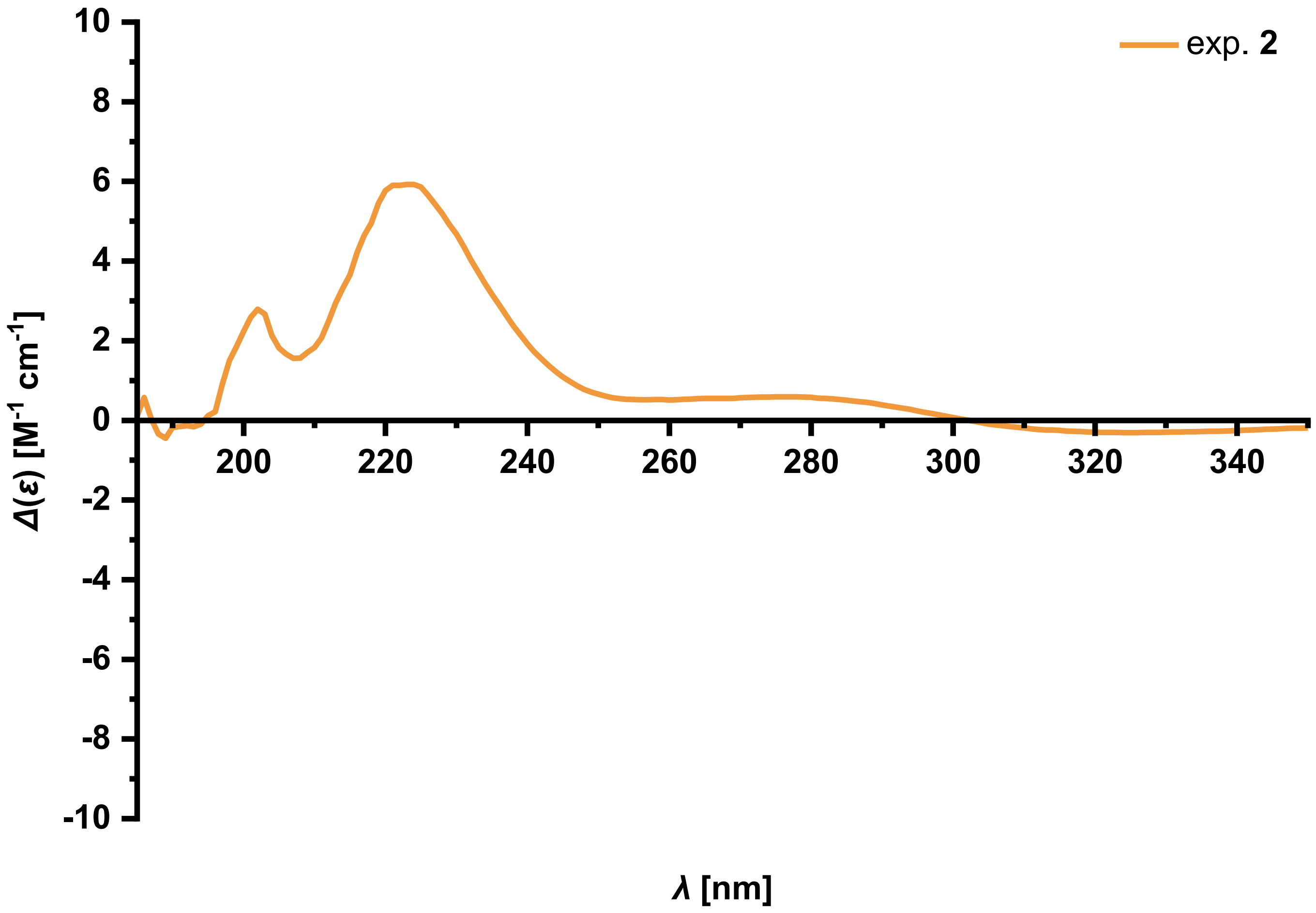

Figure S40: Experimental ECD spectrum for **2.**
