# Supplementary material for: Dryinones: Structure Elucidation of Red Colorants from Submerged Cultures of Pleurotus dryinus
Source: J Nat Prod. 2025 Nov 3;88(11):2602–9. doi: 10.1021/acs.jnatprod.5c00926 (PMC12670701; doi:10.1021/acs.jnatprod.5c00926)
Supplement: Supplementary file 1 [file np5c00926_si_001.zip › NMR Data Dryinone A (1)/1H 15N HMBC/pdata/1/email_Sep24-2024_700_NB_82_1.pdf]

Broel PDR2 6a

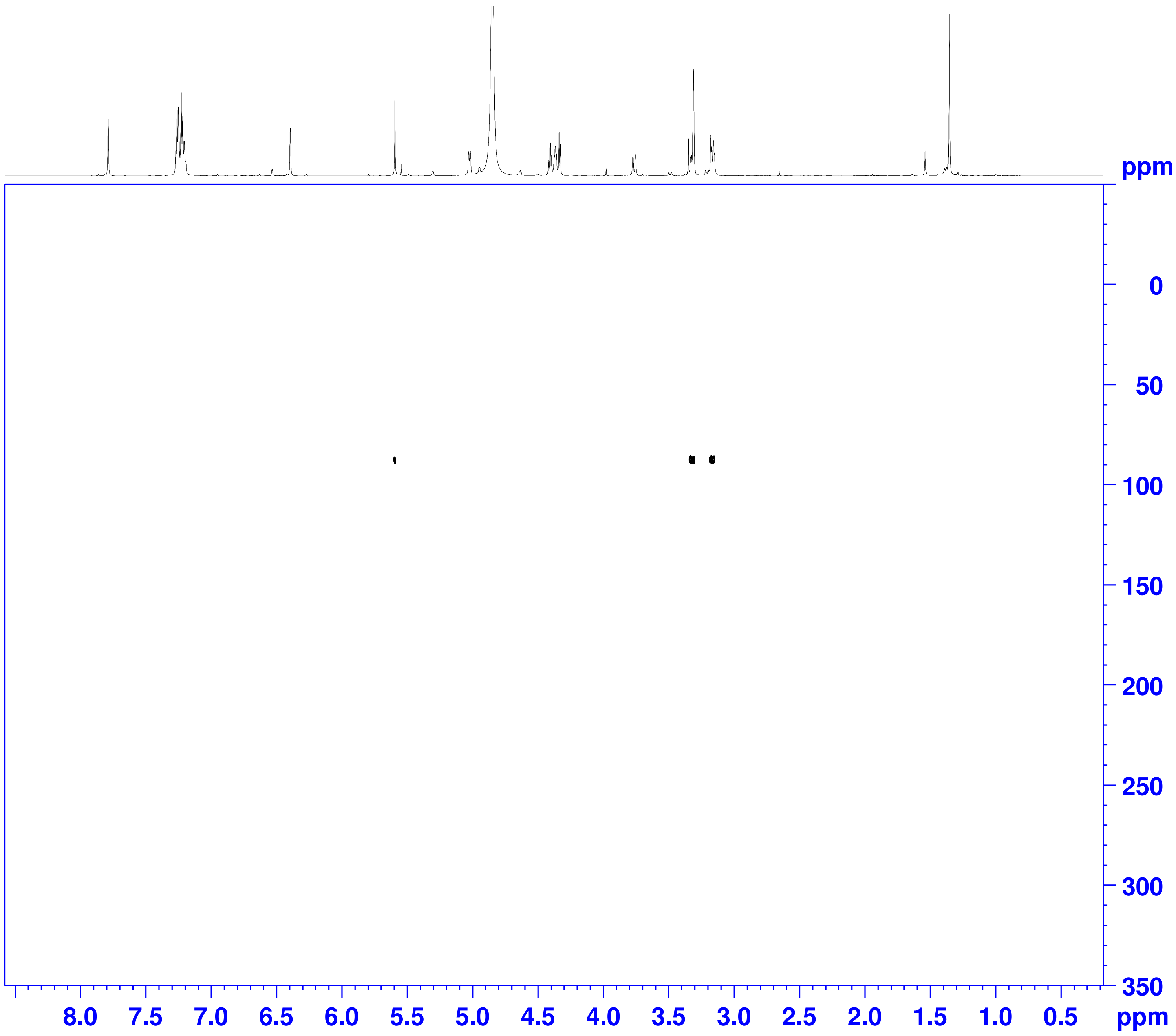


```
Current Data Parameters
NAME      Sep24-2024_700_NB
EXPNO                    82
PROCNO                    1

F2 - Acquisition Parameters
Date_              20240924
Time                  13.56 h
INSTRUM           Avance Neo
PROBHD   Z168794_0004 (
PULPROG        hmbcgpndqf
TD                     4096
SOLVENT                MeOD
NS                        8
DS                       16
SWH                5882.353 Hz
FIDRES             2.872243 Hz
AQ                0.3481600 sec
RG                      101
DW                   85.000 usec
DE                    10.00 usec
TE                    303.0 K
CNST13            5.0000000
D0               0.00000300 sec
D1               2.00000000 sec
D6               0.10000000 sec
D16              0.00020000 sec
IN0              0.00001761 sec
TDav                      1
SFO1            700.2830792 MHz
NUC1                     1H
P1                     7.98 usec
P2                    15.96 usec
PLW1            13.90999985 W
SFO2             70.9694000 MHz
NUC2                    15N
P3                    32.00 usec
PLW2           210.44000244 W
GPNAM[1]          SMSQ10.100
GPZ1                  70.00 %
GPNAM[2]          SMSQ10.100
GPZ2                  30.00 %
GPNAM[3]          SMSQ10.100
GPZ3                  50.10 %
P16                 1000.00 usec

======== F1 INDIRECT DIMENSION ========
td1        128
sw_F1      400.000000

F1 - Acquisition parameters
TD                      128
SFO1                70.9694 MHz
FIDRES           443.558746 Hz
SW                  400.000 ppm
FnMODE                   QF

F2 - Processing parameters
SI                     4096
SF              700.2800138 MHz
WDW                   QSINE
SSB                       0
LB                        0 Hz
GB                        0
PC                     1.40

F1 - Processing parameters
SI                     1024
MC2                      QF
SF               70.9587562 MHz
WDW                   QSINE
SSB                       0
LB                        0 Hz
GB                        0
```
