# Supplementary material for: Dryinones: Structure Elucidation of Red Colorants from Submerged Cultures of Pleurotus dryinus
Source: J Nat Prod. 2025 Nov 3;88(11):2602–9. doi: 10.1021/acs.jnatprod.5c00926 (PMC12670701; doi:10.1021/acs.jnatprod.5c00926)
Supplement: Supplementary file 1 [file np5c00926_si_001.zip › NMR Data Dryinone A (1)/1H 1H COSY/pdata/1/email_Sep24-2024_700_NB_81_1.pdf]

Broel PDR2 6a

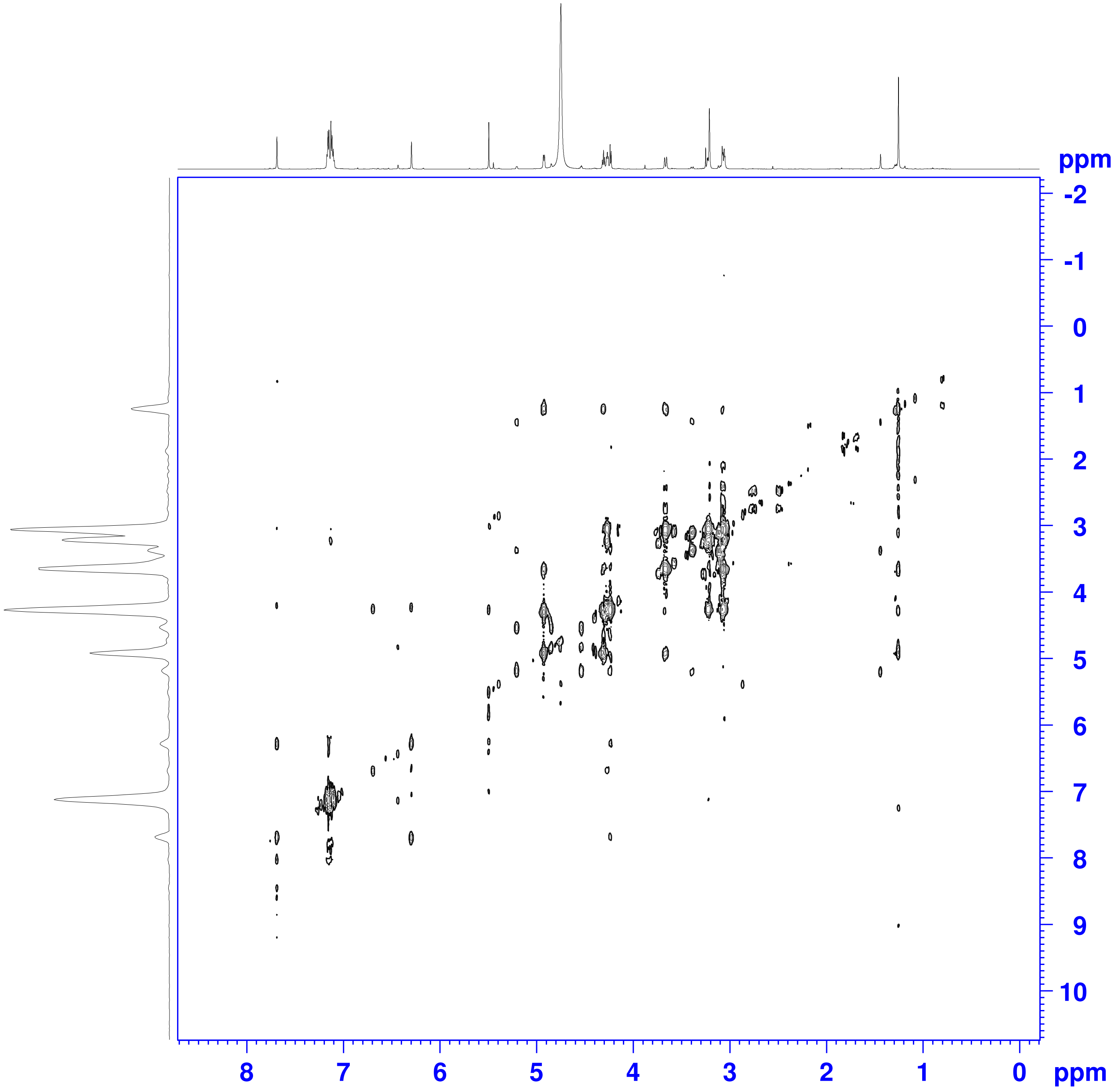


```
Current Data Parameters
NAME      Sep24-2024_700_NB
EXPNO                    81
PROCNO                    1

F2 - Acquisition Parameters
Date_              20240924
Time                  13.08 h
INSTRUM          Avance Neo
PROBHD    Z168794_0004 (
PULPROG         cosygpmfqf
TD                     2048
SOLVENT                MeOD
NS                        4
DS                       16
SWH                6250.000 Hz
FIDRES             6.103516 Hz
AQ                0.1638400 sec
RG                      101
DW                   80.000 usec
DE                    10.00 usec
TE                    303.0 K
D0               0.00000300 sec
D1               1.94675195 sec
D13              0.00000400 sec
D16              0.00020000 sec
IN0              0.00011000 sec
TDav                      1
SFO1            700.2830619 MHz
NUC1                     1H
P1                     7.98 usec
PLW1            13.90999985 W
GPNAM[1]          SMSQ10.100
GPZ1                  16.00 %
GPNAM[2]          SMSQ10.100
GPZ2                  12.00 %
GPNAM[3]          SMSQ10.100
GPZ3                  40.00 %
P16                 1000.00 usec

======== F1 INDIRECT DIMENSION ========
td1       128
sw_F1     12.981763

F1 - Acquisition parameters
TD                      128
SFO1            700.2831 MHz
FIDRES        142.045456 Hz
SW                   12.982 ppm
FnMODE                   QF

F2 - Processing parameters
SI                     1024
SF              700.2800837 MHz
WDW                    SINE
SSB                       0
LB                        0 Hz
GB                        0
PC                     1.40

F1 - Processing parameters
SI                     1024
MC2                      QF
SF              700.2800837 MHz
WDW                    SINE
SSB                       0
LB                        0 Hz
GB                        0
```
