# Supplementary material for: Dryinones: Structure Elucidation of Red Colorants from Submerged Cultures of Pleurotus dryinus
Source: J Nat Prod. 2025 Nov 3;88(11):2602–9. doi: 10.1021/acs.jnatprod.5c00926 (PMC12670701; doi:10.1021/acs.jnatprod.5c00926)
Supplement: Supplementary file 1 [file np5c00926_si_001.zip › NMR Data Dryinone A (1)/JMOD/pdata/1/email_Sep24-2024_700_NB_87_1.pdf]

Broel PDR2 6a

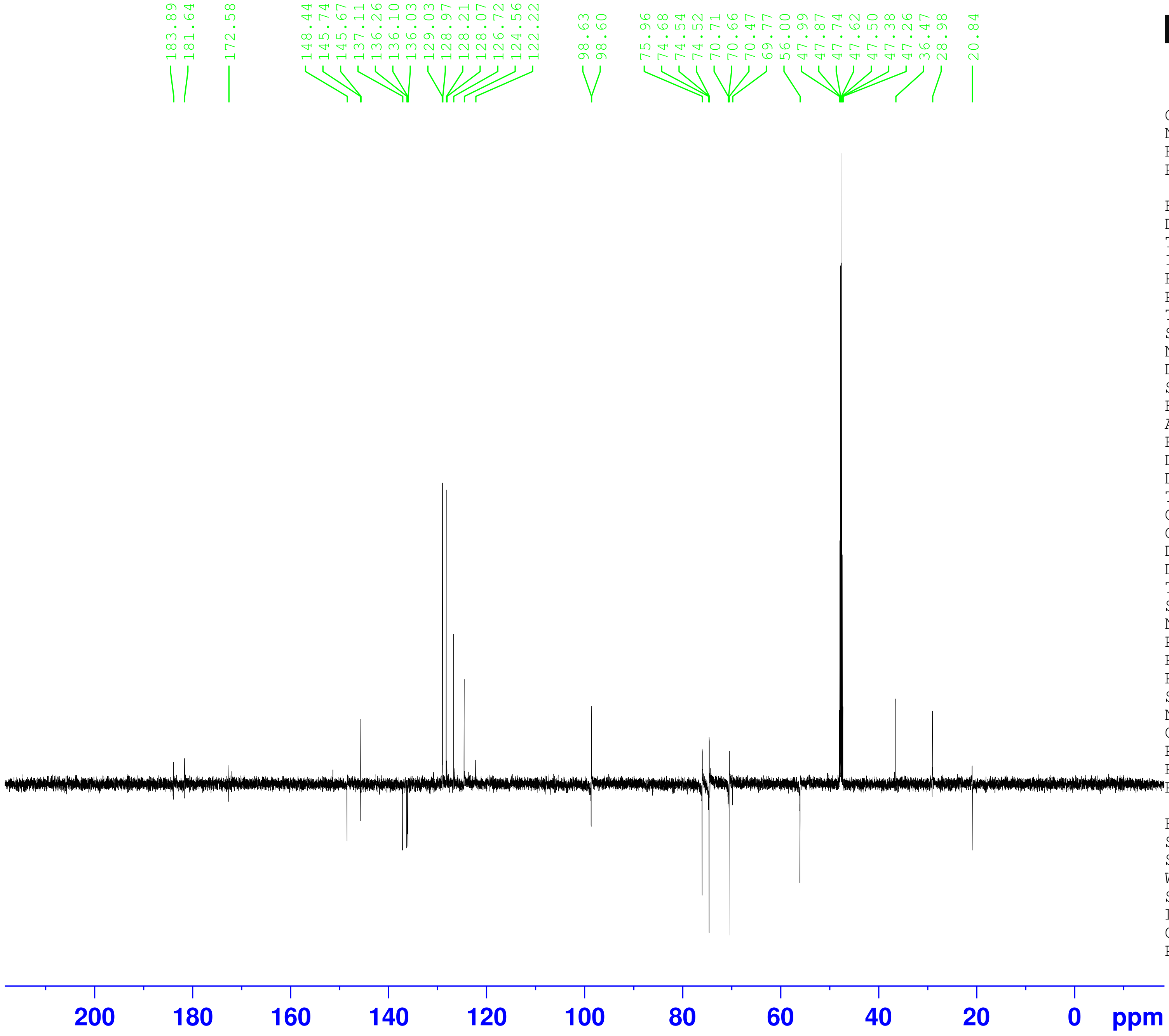


```
Current Data Parameters
NAME        Sep24-2024_700_NB
EXPNO                      87
PROCNO                      1

F2 - Acquisition Parameters
Date_                20240924
Time                    18.21 h
INSTRUM            Avance Neo
PROBHD      Z168794_0004 (
PULPROG                  jmod
TD                      65536
SOLVENT                  MeOD
NS                        256
DS                          4
SWH                 41666.668 Hz
FIDRES               1.271566 Hz
AQ                  0.7864320 sec
RG                        101
DW                     12.000 usec
DE                      18.00 usec
TE                      303.0 K
CNST2             145.0000000
CNST11              1.0000000
D1                 2.00000000 sec
D20                0.00689655 sec
TD0                         1
SFO1              176.1031546 MHz
NUC1                      13C
P1                      12.00 usec
P2                      24.00 usec
PLW1             110.76999664 W
SFO2              700.2828011 MHz
NUC2                       1H
CPDPRG[2              waltz65
PCPD2                   80.00 usec
PLW2              13.90999985 W
PLW12              0.13841000 W

F2 - Processing parameters
SI                      32768
SF                176.0855461 MHz
WDW                        EM
SSB                         0
LB                       1.00 Hz
GB                          0
PC                       1.40
```
