# Supplementary material for: Dryinones: Structure Elucidation of Red Colorants from Submerged Cultures of Pleurotus dryinus
Source: J Nat Prod. 2025 Nov 3;88(11):2602–9. doi: 10.1021/acs.jnatprod.5c00926 (PMC12670701; doi:10.1021/acs.jnatprod.5c00926)
Supplement: Supplementary file 2 [file np5c00926_si_002.zip › NMR Data Dryinone B (2)/1H 1H COSY/pdata/1/email_Oct08-2024_700_NBr_91_1.pdf]

Broel

PDR2_6b

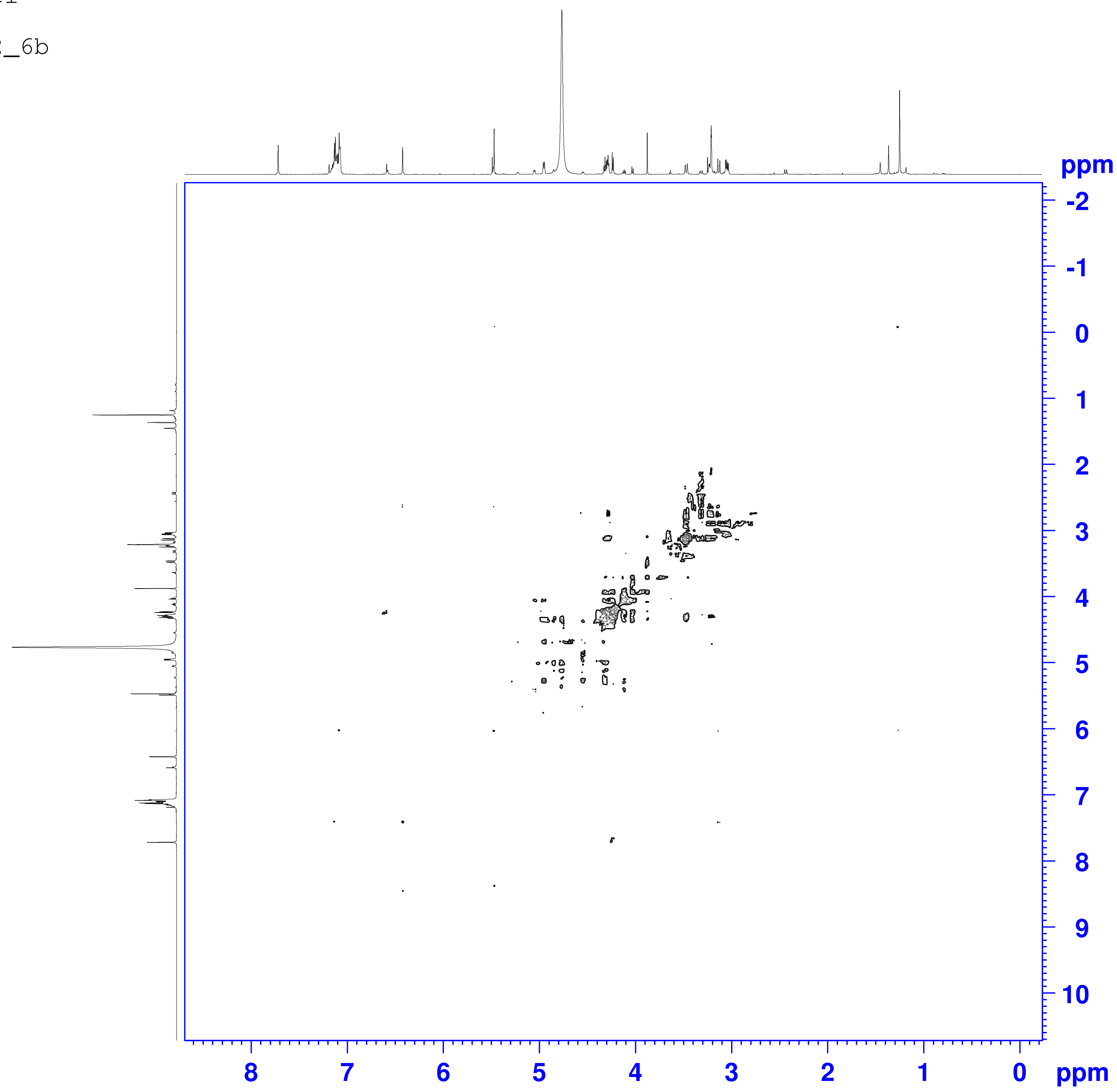


```
Current Data Parameters
NAME      Oct08-2024_700_NBr
EXPNO                     91
PROCNO                     1

F2 - Acquisition Parameters
Date_               20241008
Time                   13.06 h
INSTRUM           Avance Neo
PROBHD    Z168794_0004 (
PULPROG          cosygpppqf
TD                      2048
SOLVENT                 MeOD
NS                         1
DS                        16
SWH                 6250.000 Hz
FIDRES              6.103516 Hz
AQ                 0.1638400 sec
RG                       101
DW                    80.000 usec
DE                     10.00 usec
TE                     303.0 K
D0                0.00000300 sec
D1                1.94675195 sec
D11               0.03000000 sec
D12               0.00002000 sec
D13               0.00000400 sec
D16               0.00020000 sec
IN0               0.00011000 sec
TDav                       1
SFO1             700.2830445 MHz
NUC1                      1H
P0                      7.98 usec
P1                      7.98 usec
P17                  2500.00 usec
PLW1             13.90999985 W
PLW10             1.41729999 W
GPNAM[1]          SMSQ10.100
GPZ1                   10.00 %
P16                  1000.00 usec

======== F1 INDIRECT DIMENSION ========
td1       256
sw_F1     12.981764

F1 - Acquisition parameters
TD                       256
SFO1                 700.283 MHz
FIDRES             71.022728 Hz
SW                    12.982 ppm
FnMODE                    QF

F2 - Processing parameters
SI                      1024
SF               700.2800833 MHz
WDW                    QSINE
SSB                        0
LB                         0 Hz
GB                         0
PC                      1.40

F1 - Processing parameters
SI                      1024
MC2                       QF
SF               700.2800833 MHz
WDW                    QSINE
SSB                        0
LB                         0 Hz
GB                         0
```
