# Supplementary material for: Dryinones: Structure Elucidation of Red Colorants from Submerged Cultures of Pleurotus dryinus
Source: J Nat Prod. 2025 Nov 3;88(11):2602–9. doi: 10.1021/acs.jnatprod.5c00926 (PMC12670701; doi:10.1021/acs.jnatprod.5c00926)
Supplement: Supplementary file 2 [file np5c00926_si_002.zip › NMR Data Dryinone B (2)/1H 1H COSY 2/pdata/1/email_Oct08-2024_700_NBr_102_1.pdf]

Broel

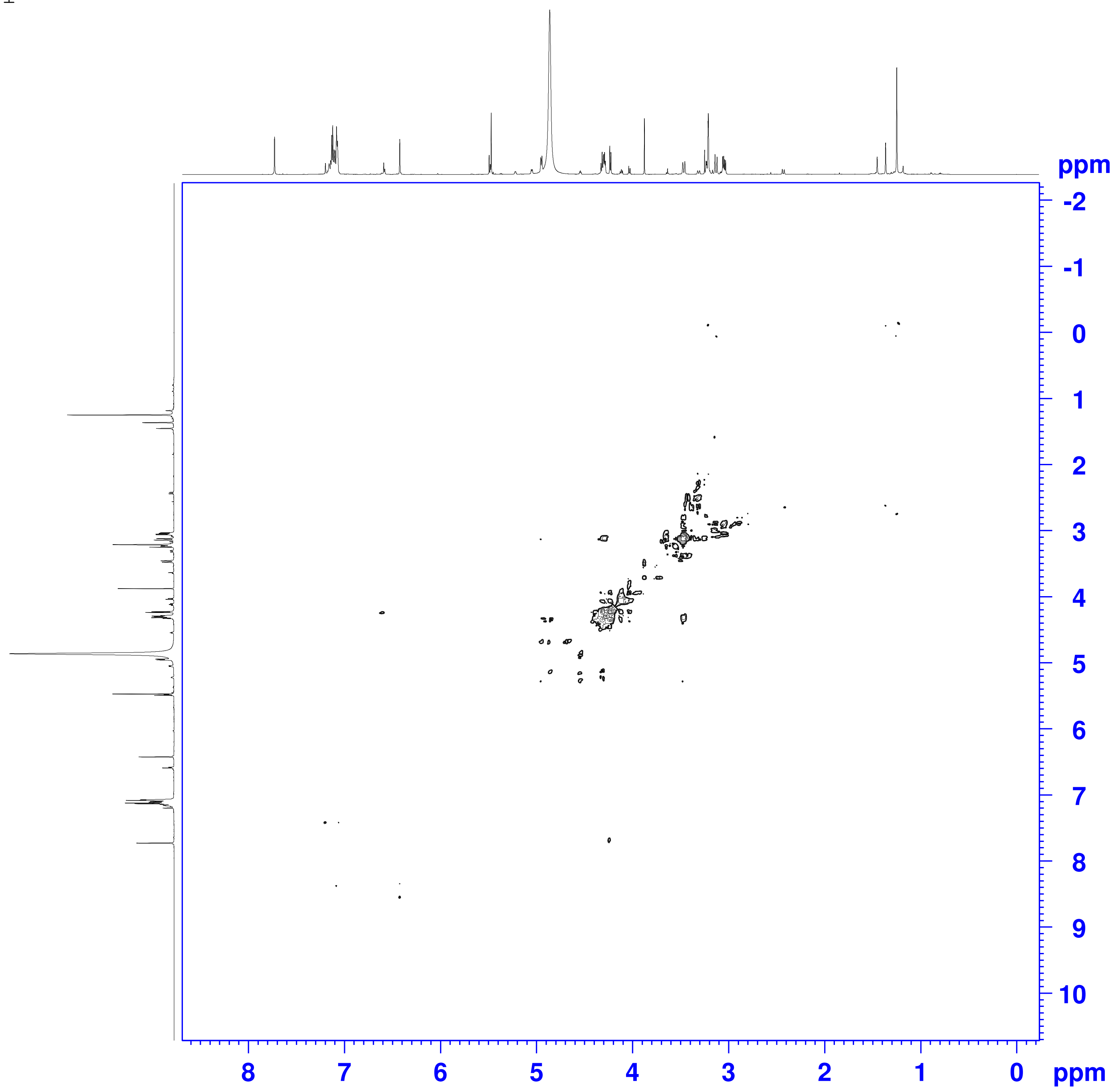

Current Data Parameters

NAME	Oct08-2024_700_NBr
EXPNO	102
PROCNO	1

F2 - Acquisition Parameters

Date_	20241008
Time	13.32 h
INSTRUM	Avance Neo
PROBHD	Z168794_0004 (
PULPROG	cosygpppqf
TD	2048
SOLVENT	MeOD
NS	1
DS	16
SWH	6250.000 Hz
FIDRES	6.103516 Hz
AQ	0.1638400 sec
RG	101
DW	80.000 usec
DE	10.00 usec
TE	293.0 K
D0	0.00000300 sec
D1	1.94675195 sec
D11	0.03000000 sec
D12	0.00002000 sec
D13	0.00000400 sec
D16	0.00020000 sec
IN0	0.00011000 sec
TDav	1
SFO1	700.2830437 MHz
NUC1	1H
P0	7.98 usec
P1	7.98 usec
P17	2500.00 usec
PLW1	13.90999985 W
PLW10	1.41729999 W
GPNAM[1]	SMSQ10.100
GPZ1	10.00 %
P16	1000.00 usec

======== F1 INDIRECT DIMENSION ========

td1	256
sw_F1	12.981764

F1 - Acquisition parameters

TD	256
SFO1	700.283 MHz
FIDRES	71.022728 Hz
SW	12.982 ppm
FnMODE	QF

F2 - Processing parameters

SI	1024
SF	700.2800839 MHz
WDW	QSINE
SSB	0
LB	0 Hz
GB	0
PC	1.40

F1 - Processing parameters

SI	1024
MC2	QF
SF	700.2800839 MHz
WDW	QSINE
SSB	0
LB	0 Hz
GB	0
